# Enzymatic analysis of WWP2 E3 ubiquitin ligase using protein microarrays identifies autophagy-related substrates

**DOI:** 10.1016/j.jbc.2022.101854

**Published:** 2022-03-21

**Authors:** Hanjie Jiang, Claire Y. Chiang, Zan Chen, Sara Nathan, Gabriel D’Agostino, Joao A. Paulo, Guang Song, Heng Zhu, Sandra B. Gabelli, Philip A. Cole

**Affiliations:** 1 Division of Genetics, Department of Medicine, Brigham and Women’s Hospital, Boston, Massachusetts, USA; 2 Department of Biological Chemistry and Molecular Pharmacology, Harvard Medical School, Boston, Massachusetts, USA; 3 Department of Pharmacology and Molecular Sciences, Johns Hopkins School of Medicine, Baltimore, Maryland, USA; 4 Department of Cell Biology, Harvard Medical School, Boston, Massachusetts, USA; 5 Department of Biophysics and Biophysical Chemistry, The Johns Hopkins School of Medicine, Baltimore, Maryland, USA; 6 Department of Oncology, The Johns Hopkins University School of Medicine, Baltimore, Maryland, USA; 7 Department of Medicine, The Johns Hopkins University School of Medicine, Baltimore, Maryland, USA

**Keywords:** ubiquitin ligase, enzyme, autophagy receptor, NEDD4, Lys modification, BSA, bovine serum albumin, CCCP, carbonyl cyanide chlorophenylhydrazone, MST, microscale thermophoresis, OPTN, optineurin, SQSTM1, sequestosome, TCEP, tris-carboxyethyl-phosphine, Ub, ubiquitin, UbV, ubiquitin variant

## Abstract

WWP2 is a HECT E3 ligase that targets protein Lys residues for ubiquitination and is comprised of an N-terminal C2 domain, four central WW domains, and a C-terminal catalytic HECT domain. The peptide segment between the middle WW domains, the 2,3-linker, is known to autoinhibit the catalytic domain, and this autoinhibition can be relieved by phosphorylation at Tyr369. Several protein substrates of WWP2 have been identified, including the tumor suppressor lipid phosphatase PTEN, but the full substrate landscape and biological functions of WWP2 remain to be elucidated. Here, we used protein microarray technology and the activated enzyme phosphomimetic mutant WWP2^Y369E^ to identify potential WWP2 substrates. We identified 31 substrate hits for WWP2^Y369E^ using protein microarrays, of which three were known autophagy receptors (NDP52, OPTN, and SQSTM1). These three hits were validated with *in vitro* and cell-based transfection assays and the Lys ubiquitination sites on these proteins were mapped by mass spectrometry. Among the mapped ubiquitin sites on these autophagy receptors, many had been previously identified in the endogenous proteins. Finally, we observed that WWP2 KO SH-SH5Y neuroblastoma cells using CRISPR-Cas9 showed a defect in mitophagy, which could be rescued by WWP2^Y369E^ transfection. These studies suggest that WWP2-mediated ubiquitination of the autophagy receptors NDP52, OPTN, and SQSTM1 may positively contribute to the regulation of autophagy

WWP2 is a HECT domain containing E3 ubiquitin (Ub) ligase that has been shown to target a range of protein substrates in physiological and pathophysiological processes (1, 2, 3, 4, 5, 6). WWP2 has an N-terminal C2 domain followed by four WW domains and culminates in a catalytic HECT domain. The N-terminal C2 domain is proposed to be involved in Ca^2+^/phospholipid binding, whereas the WW domains have been implicated in mediating protein–protein interactions with PPXY motif-containing proteins (7). Unlike the more numerous RING domain E3 ubiquitin ligases, the 28 HECT domain E3 ubiquitin ligases possess a catalytic Cys residue (8). The HECT active site Cys undergoes a trans-thioesterification reaction with the upstream E2 Cys-ubiquitin to generate an E3 covalent intermediate that in turn reacts with protein Lys residues (9, 10, 11).

Regulation of WWP2 is in part governed by a ∼30 amino acid linker, known as the 2,3-linker, that connects the second and third WW domains (12, 13, 14). In the ground state, the 2,3-linker forms extensive intramolecular interactions with the WWP2 HECT domain, occluding WWP2’s allosteric ubiquitin-binding site, which is required for enzyme activity, and freezing the HECT conformation in an inverted T shape (12). These 2,3-linker-HECT interactions therefore lead to autoinhibition of WWP2. Relief of this autoinhibition can be achieved by at least three mechanisms: Ub or engineered Ub variants binding to the allosteric ubiquitin-binding site, multi-WW domain engagement by proteins like Ndfip1 with multiple proline-rich (PPXY or LPXY) motifs, or phosphorylation of the 2,3-linker (12, 14, 15, 16, 17, 18, 19, 20).

There have been several ubiquitination substrates reported for WWP2, including the transcription factors OCT4 and EGR2 (21, 22), the catalytic subunit of RNA polymerase II (23, 24), the signaling protein I-SMAD7 (25), the RNA-editing enzyme ADAR2 (26), and the tumor suppressor lipid phosphatase PTEN (1, 27). WWP2 is most closely related to HECT paralogs WWP1 and Itch and to a more limited extent to HECT enzymes NEDD4 and NEDD4L, each of which have been linked to a range of key cellular substrates (5, 28, 29, 30). WWP1, NEDD4, NEDD4L, and Itch have been connected to the intracellular degradation and recycling process of autophagy (31, 32, 33, 34, 35). Autophagy involves an orderly set of events including the assembly of macromolecular complexes, sequestration and formation of double-membrane autophagosome vesicles, and eventually autophagosome fusion with the lysosome (36, 37, 38). Autophagy is critical for cellular homeostasis and provides for constitutive turnover of cytosolic components but is also used by cells in response to various stimuli such as starvation, infection, and stress (36, 39). Among the many proteins implicated in autophagy are a set of autophagy receptors that include SQSTM1 (Sequestosome or p62), Optineurin (OPTN), and NDP52 (also known as CALCOCO2) that can selectively bring cargo to the autophagosome through their interaction with the LC3 protein, which exists as a phospholipid conjugate with the autophagosome (40, 41, 42). Recently, NEDD4 has been suggested to catalyze the ubiquitination of SQSTM1 to promote autophagy (43, 44) but also has been described as an antiautophagy enzyme due to its ubiquitination of Beclin1 and TBK1 (45, 46). NEDD4L has been reported to inhibit autophagy by ubiquitinating ULK1 (47), whereas WWP1 has been stated to be proautophagy by targeting KLF5 for ubiquitination (48). In contrast to these other NEDD4 paralogs, the potential role of WWP2 in autophagy has not been investigated.

To learn more about WWP2, here, we have employed human protein microarrays as a platform to identify direct substrates of WWP2’s E3 ligase activity across the proteome. These protein microarrays, also known as protein chips, display more than 20,000 purified recombinant human proteins produced in yeast and spatially organized on a slide (49, 50). Using an activated form of WWP2 containing a phosphomimetic in the 2,3-linker (WWP2^Y369E^), we found dozens of potential WWP2 substrates, including NDP52, OPTN, and SQSTM1. We further perform biochemical and cellular analyses of these autophagy receptors that suggest that WWP2 can ubiquitinate autophagy receptors NDP52, OPTN, and SQSTM1 in a fashion that may be important in promoting autophagy, especially mitophagy.

## Results

### WWP2 ubiquitination of protein microarrays

**Figure 1:**
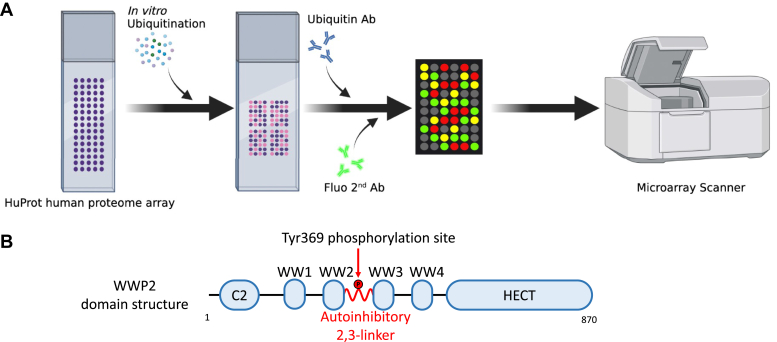
**WWP2 substrate identification with HuProt protein microarrays.***A*, the overall workflow of WWP2 substrate screening using HuProt human protein microarrays. The microarray ubiquitination reaction conditions are described in the Experimental procedures section. *B*, WWP2 protein domain architecture. The autoinhibitory 2,3-linker is shown as a *red helix*. The activating linker phosphorylation site at tyrosine 369 is highlighted.

**Figure 2:**
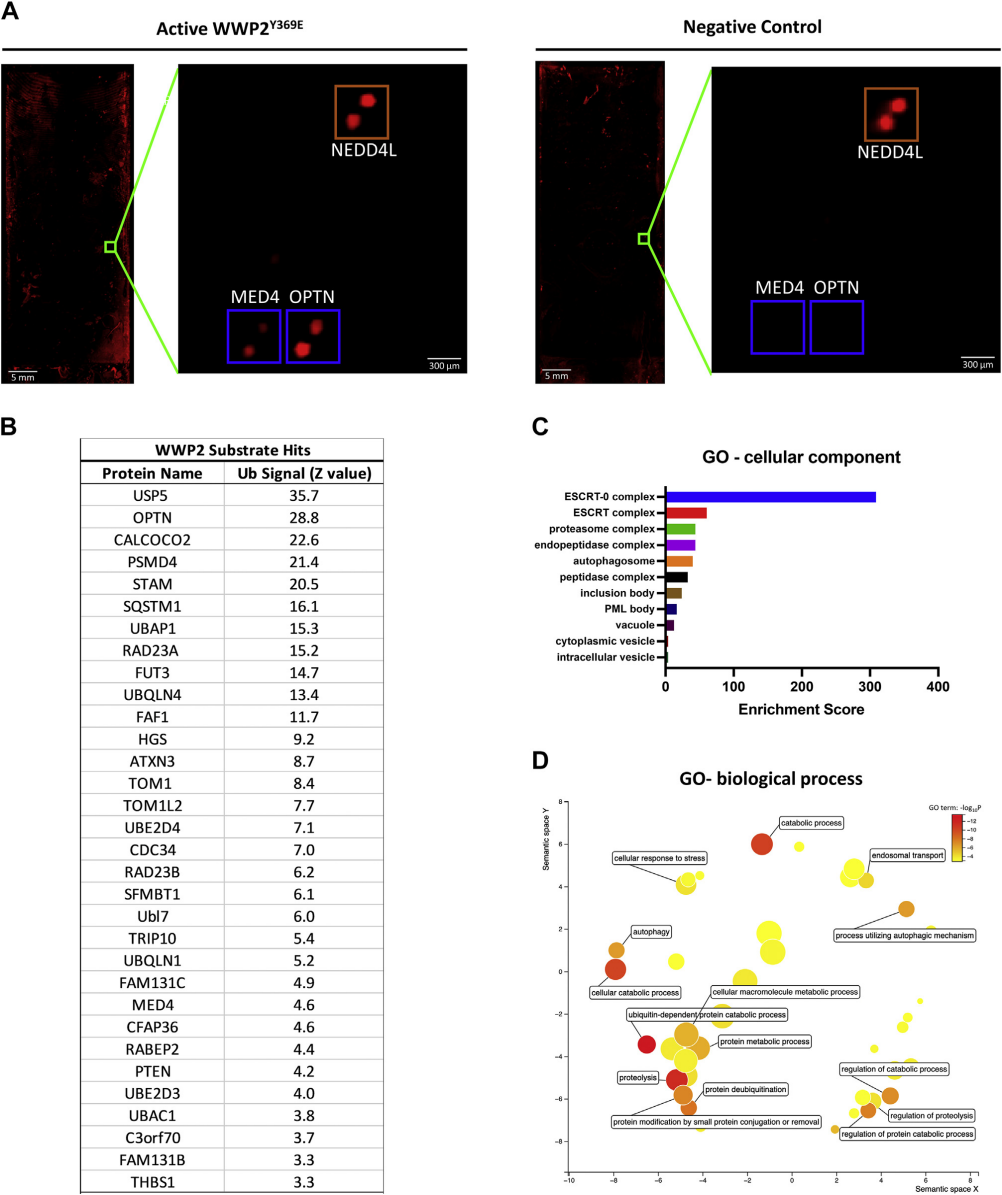
***In vitro* ubiquitination on protein microarrays identified WWP2 substrates.***A*, example of comparative zoomed-in images of representative WWP2 ubiquitination hits on the microarray chip. E3 ligase NEDD4L showed fluorescent signals on both control and active WWP2^Y369E^–treated protein microarrays. MED4 and OPTN only appeared on the active WWP2^Y369E^–treated protein microarrays. *B*, identified WWP2 E3 ligase hits using HuProt protein microarrays using linker phospho-mimetic form of WWP2. The hits are ranked by ubiquitination signal intensity/Z value. *C*, GO-cellular component enrichment analysis of WWP2 hits. The correlated components were ranked based on calculated enrichment scores using the GOrilla tool. *D*, GO-biological process enrichment analysis of WWP2 hits. The GO term (Log_10_*P*) was used for generating and visualizing the hit map using the REVIGO tool. *Note: The axes in the plot have no intrinsic meaning.* OPTN, optineurin.

To learn more about the ubiquitination substrate selectivity of WWP2 across the proteome, we pursued a human protein microarray approach. We employed HuProt human protein microarrays that display approximately 21,000 purified recombinant proteins, including over 81% of canonical expressed proteins, prepared from yeast (51). The ubiquitination reaction was reconstructed *in vitro* using recombinant E1, E2 (UbcH5b) enzymes alongside WWP2 and Ub-adapting conditions for the ubiquitination assays from previous studies using related protein microarrays (52, 53). Ubiquitination of proteins was imaged with a microarray scanner by treating the chips with an anti-Ub antibody followed by a fluorescent secondary antibody (Alexa Fluor 555) (Fig. 1*A*). To increase the potential for finding protein substrates, we used hyperactivated Y369E WWP2 (WWP2^Y369E^) E3 ligase (Fig. 1*B*) as well as, separately, WT WWP2 (WWP2^WT^) and compared the ubiquitination patterns to control chips that omitted the E3 ligase. Y369E mutation in WWP2 has been shown to be an effective phosphomimetic for Tyr phosphorylation of this position, relieving the autoinhibition of Ub ligase activity by loosening intramolecular HECT domain interactions with the 2,3-linker (12, 14). Positive hits were called based on a combination of statistical analysis (>3 SDs above the median spot fluorescence intensity across protein microarray) and visual inspection across duplicate experiments, compared with the signals for the corresponding spots on the replicate control chips (minus WWP2). Representative positive hits are displayed and illustrate the paired spots that show signals on the WWP2-treated chip but not corresponding positions on the control chip for a presumed WWP2 protein substrate (Fig. 2*A*, MED4 and OPTN are shown as examples of positive hits). In contrast, the NEDD4L was found to be ubiquitinated on both the control chips and the WWP2^Y369E^ chips, visualized as the paired bright spots, and was not counted as a WWP2^Y369E^ substrate hit (See Table S1). In this way, we found that there were 31 hits on the WWP2^Y369E^-treated chips and only three on the WWP2^WT^-related chips (Fig. 2*B*, Tables S2 and S3). The greater number of hits and enhanced ubiquitination signals on the WWP2^Y369E^ chips were reassuring given the known increased catalytic activity conferred by this phosphomimetic mutation.

**Figure 3:**
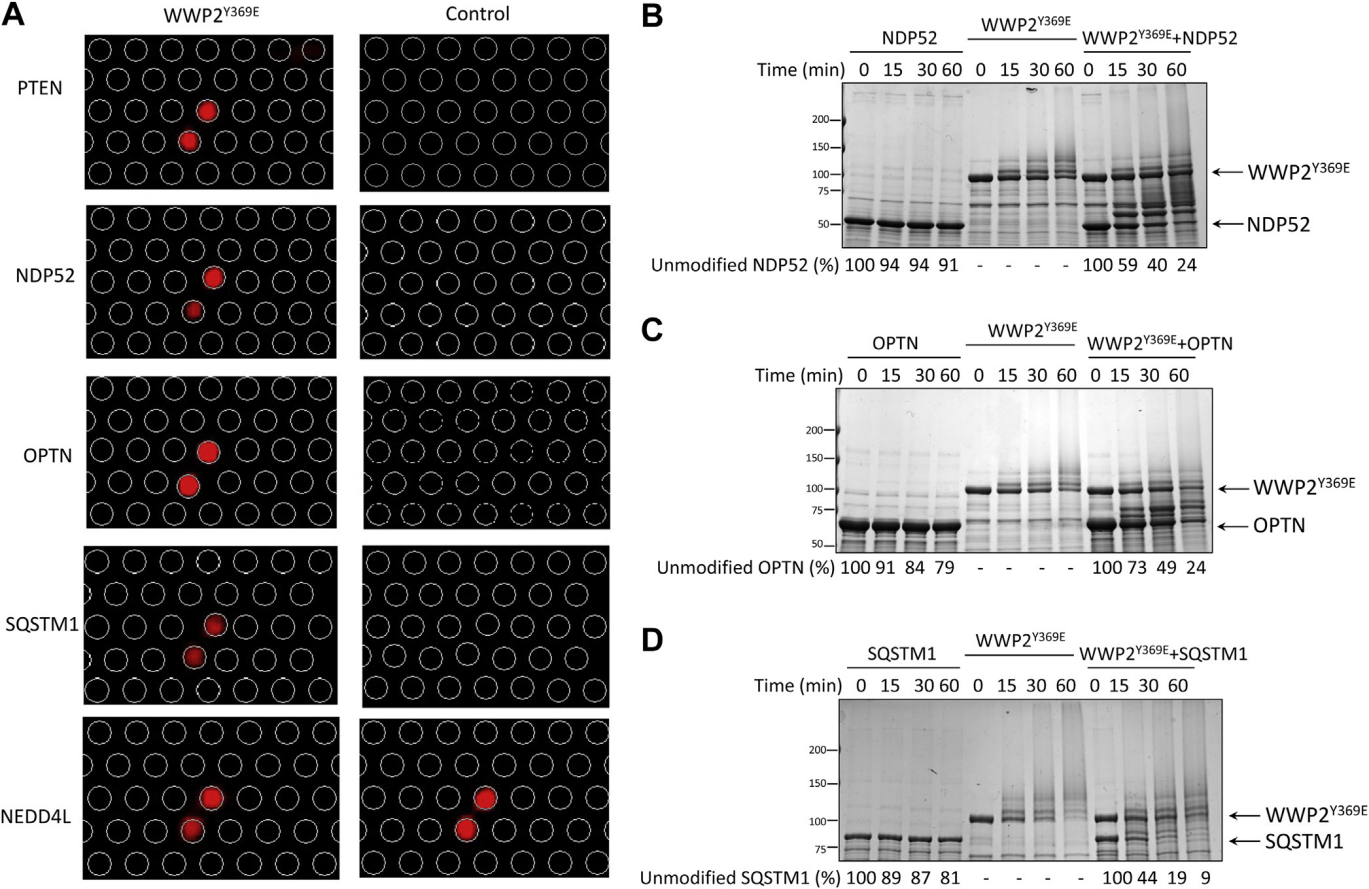
**Validation of NDP52, OPTN, and SQSTM1 as WWP2 substrates using *in vitro* ubiquitination assays.***A*, close-up views of selected hits studied in this research, including NDP52, OPTN, and SQSTM1. PTEN is shown as a positive control and NEDD4L is shown as a negative control. *B*, ubiquitination assay of WWP2 with NDP52. NDP52 lacks autoubiquitination activity when the E3 ligase WWP2 is absent. Ubiquitination of NDP52 protein is observed as progressive ladder bands or smearing when WWP2 is present. *C*, ubiquitination assay of WWP2 with OPTN. OPTN lacks autoubiquitination activity when the E3 ligase WWP2 is absent. Ubiquitination of OPTN protein is observed as progressive ladder bands or smearing when WWP2 was present. *D*, ubiquitination assay of WWP2 with SQSTM1. SQSTM1 lacks autoubiquitination activity when the E3 ligase WWP2 was absent. Ubiquitination of SQSTM1 protein is observed as progressive ladder bands or smearing when WWP2 is present. Gels are stained with Colloidal Blue. The depletion of the unmodified substrate proteins was determined by densitometry analysis and shown as a percentage compared to the samples at time zero. OPTN, optineurin; SQSTM1, sequestosome.

We performed GO enrichment analysis of the 31 identified hits from WWP2^Y369E^ chips based on human whole proteome database (http://geneontology.org/). The GO cellular component (Fig. 2*C*) and biological process enrichment (Fig. 2*D*) of these were visualized using the GOrilla and REVEGO tools (54, 55). The hits identified were preferentially associated with the following multiprotein complexes and processes: the ESCRT cellular trafficking complex, the proteasome complex, the endopeptidase complex, metabolic and catabolic pathways, as well as autophagy (Fig. 2, *C* and *D*). Of the 31 hits, one of the well-established WWP2 ubiquitin ligase substrates, PTEN, was observed (Fig. 2*B*). In addition, several known WWP2 interactors were identified (Fig. 2*B*), including USP5, TOM1, CDC34, and UbE2D3, of which the latter two are known to be E2 Ub–conjugating enzymes. Our attention was drawn to the presence of the three hits, NDP52, OPTN, and SQSTM1, as these are all well-established autophagy receptors (Fig. 3*A*). As discussed, the related WWP2 HECT E3 ligase family member, NEDD4, has been shown to positively regulate autophagy by directly ubiquitinating SQSTM1 (43, 44). We thus decided to further pursue the three identified autophagy receptors with biochemical and cellular assays as described below.

### Enzymatic analysis of WWP2 with NDP52, OPTN, and SQSTM1 purified proteins

We prepared from *E. coli* and purified recombinant full-length human NDP52, OPTN, and SQSTM1 proteins and examined their properties as WWP2 substrates using *in vitro* ubiquitination assays, employing similar conditions as those in the protein microarray screening. The ubiquitination samples were visualized with colloidal blue–stained SDS-PAGE gels that allow concomitant measurements of autoubiquitination and substrate ubiquitination. In these experiments, each of these autophagy receptors was observed to be efficiently ubiquitinated by WWP2^Y369E^ in a solution phase assay in a time-dependent fashion (Fig. 3, *B*–*D*). The rates for each of these substrates were faster than for the validated protein substrate PTEN. At 60 min, the nonubiquitinated protein levels of NDP52 were 24%, for OPTN, 24%, and for SQSTM1, 9%, compared to 73% of PTEN as we previously reported (14).

**Figure 4:**
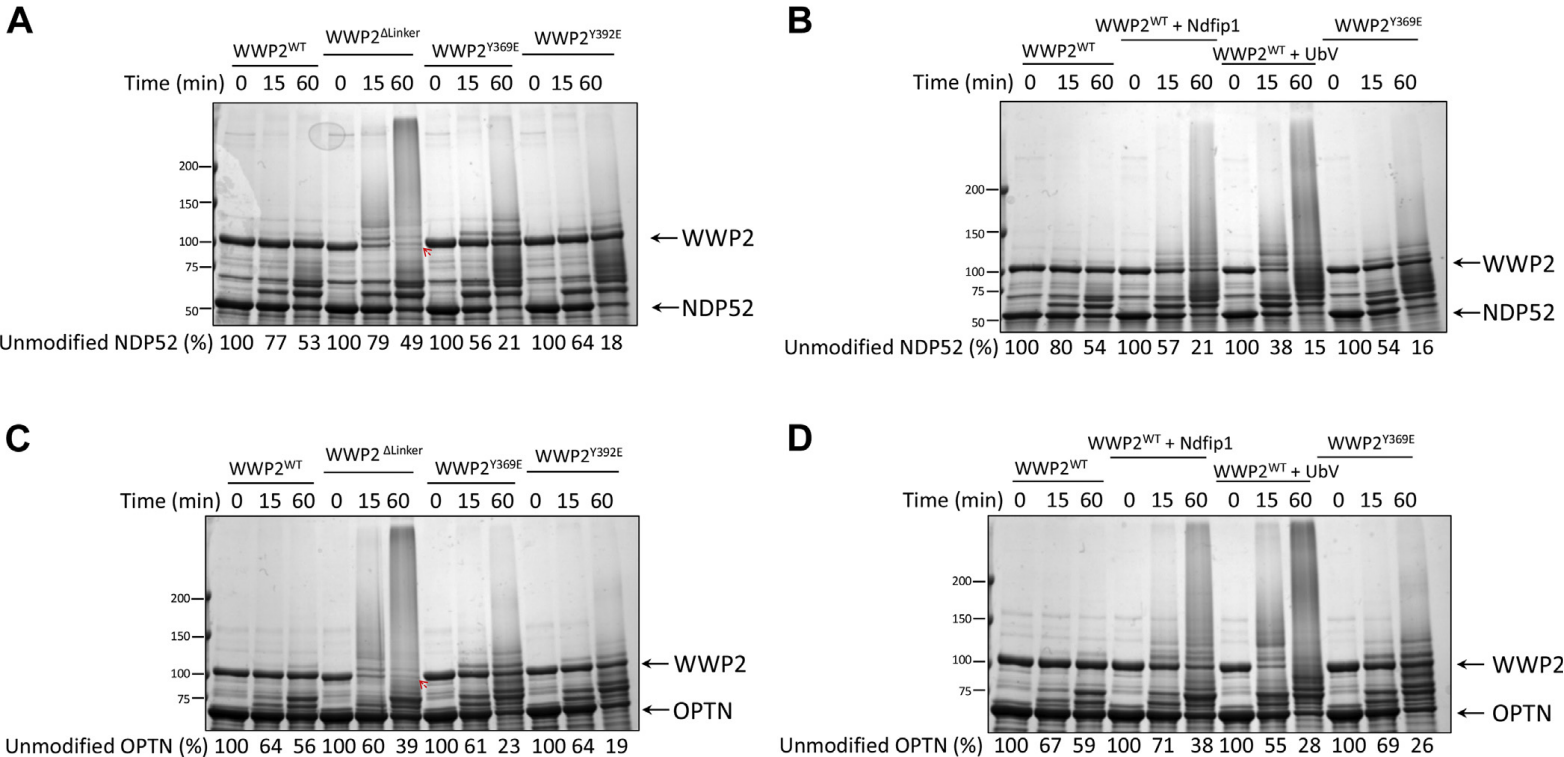
**Enzymatic analysis of NDP52 and OPTN as WWP2 substrates.***A*, *in vitro* ubiquitination assay of WWP2 with NDP52. Different forms of WWP2 E3 ligase were used for the ubiquitination of NDP52. The hyper autoubiquitination activity of WWP2^Δlinker^ was highlighted by the *red arrow*. *B*, ubiquitination assay of NDP52 in the presence of allosteric activators. WT WWP2 with either Ndfip1 (activator with the proline-rich motifs that interacts with WW domains and activates WWP2) or UbV (allosteric activator that binds to the ubiquitin exosite in the catalytic HECT domain) showed an increased extent of ubiquitination on NDP52. *C*, *in vitro* ubiquitination assay of WWP2 with OPTN. Different forms of WWP2 E3 ligase (wt; Δlinker; Y369E; Y392E) were used for the ubiquitination of OPTN. The hyper autoubiquitination activity of WWP2^Δlinker^ was highlighted by the *red arrow*. *D*, ubiquitination assay of OPTN in the presence of allosteric activators. WT WWP2 with either Ndfip1 or UbV showed an increased extent of ubiquitination on OPTN. Gels are stained with Colloidal Blue. The depletion of the unmodified substrate proteins was determined by densitometry analysis and shown as a percentage compared to the samples at time zero. Δlinker, hyperactive form with 2,3-linker removed; Y369E or Y392E, the active form of WWP2 with linker phosphorylation mimetics; OPTN, optineurin; UbV, ubiquitin variant.

As SQSTM1 has been enzymatically analyzed with WWP2 in our previous research (14), we focused on NDP52 and OPTN to assess the role of 2,3-linker phosphorylation and treatment with allosteric modulators Ndfip1 and ubiquitin (using the high affinity nonsubstrate ubiquitin variant, UbV (17)) to further characterize these autophagy receptor substrates. Compared to WWP2^WT^, both WWP2^Y369E^ and WWP2^Y392E^ (linker phosphorylation of Tyr392 could also activate WWP2 as previously reported (12)) forms showed more rapid ubiquitination of both NDP52 and OPTN (Fig. 4, *A* and *C*). Moreover, the addition of the multi-pronged WW domain binder Ndfip1, as well as UbV, accelerated the ubiquitination of NDP52 and OPTN by WWP2^WT^, consistent with their previously reported effects (12, 14, 17) as molecules that can displace the 2,3-linker from binding to the HECT domain (Fig. 4, *B* and *D*). Interestingly, 2,3-linker deletion did not increase NDP52 or OPTN substrate ubiquitination despite the dramatically stimulating effect on autoubiquitination (shown with red arrows in Fig. 4, *A* and *C*). This behavior has been seen previously with PTEN (12, 14).

### Binding and mass spectrometric analysis of WWP2 with the autophagy receptors

**Figure 5:**
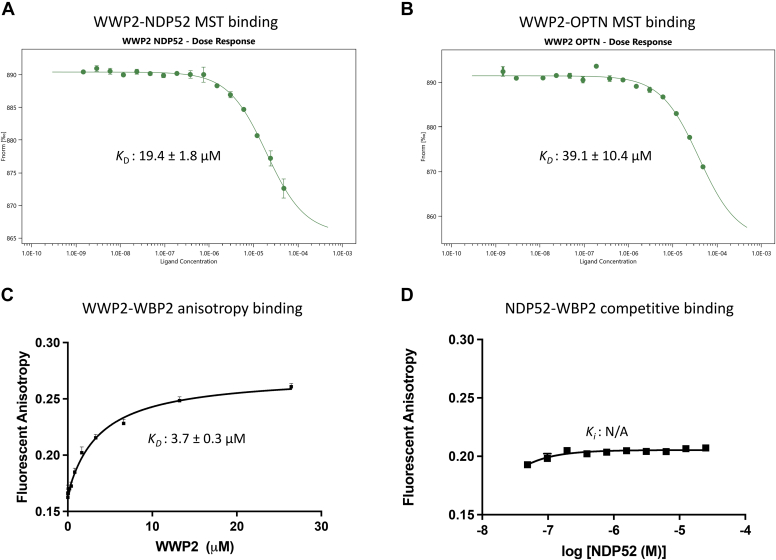
**MST and fluorescence anisotropy analysis for WWP2 and autophagy receptors.***A*, binding affinity measurement between NDP52 and WWP2 using MST. Cy5 N-terminal-labeled-WWP2 was used for the measurement. Apparent *K*_D_ is shown in the figure. *B*, binding affinity measurement between OPTN and WWP2 by MST. Apparent *K*_D_ is shown in the figure. *C*, binding affinity measurement of conventional substrate WBP2 and WWP2 by fluorescence anisotropy. Fluorescein N-terminal-labeled-WBP2 was used. *D*, competitive anisotropy binding experiment of NDP52 and WBP2. N-terminal fluorescently (FAM) labeled WBP2 was used. A constant concentration (5.7 μM) of WWP2 was used, and varying amounts of NDP52 were titrated in the assay. MST, microscale thermophoresis; OPTN, optineurin.

To characterize further the interaction between autophagy receptors and WWP2, we turned to microscale thermophoresis (MST) to determine the affinity of NDP52 and OPTN binding to WWP2 (56). MST measures the thermophoresis of molecules, which can correlate with the molecule size, charge, conformation, and hydration shell (57). As autophagy receptors are relatively large in molecular weight (NDP52, ∼52 kDa; OPTN, ∼66 kDa), MST provides relatively robust signals in their association with the ∼100 kDa WWP2 protein compared to fluorescence anisotropy. For this purpose, we site-specifically labeled the WWP2 N-terminus with a fluorescent probe (Cy5 NHS ester) as described previously (56, 58) and varied the concentration of the autophagy receptor proteins. Using this approach, the *K*_D_ of WWP2 binding to NDP52 was found to be 19 μM and for OPTN, 39 μM (Fig. 5, *A* and *B*). These values are in the same range as the previously reported PTEN interaction with WWP2 (*K*_D_ 20 μM) (58). The autophagy receptor–WWP2 interactions are 5 to 10 fold weaker than the binding of Ndfip1 to WWP2 (*K*_D_ 3.7 μM), measured by fluorescence anisotropy, which is known to engage three WW domains in WWP2 with three separate Pro-rich motifs of Ndfip1 (Fig. 5*C*). To examine if autophagy receptor and Ndfip1 binding to WWP2 were at similar or different binding sites, we determined the effect of the addition of NDP52 on the WWP2–Ndfip1 interaction. NDP52 was unable to displace Ndfip1 from WWP2, indicating that Ndfip1 and NDP52 binding to WWP2 are not mutually exclusive and NDP52’s binding to WWP2 likely does not involve WWP2’s WW domains (Fig. 5*D*).

**Figure 6:**
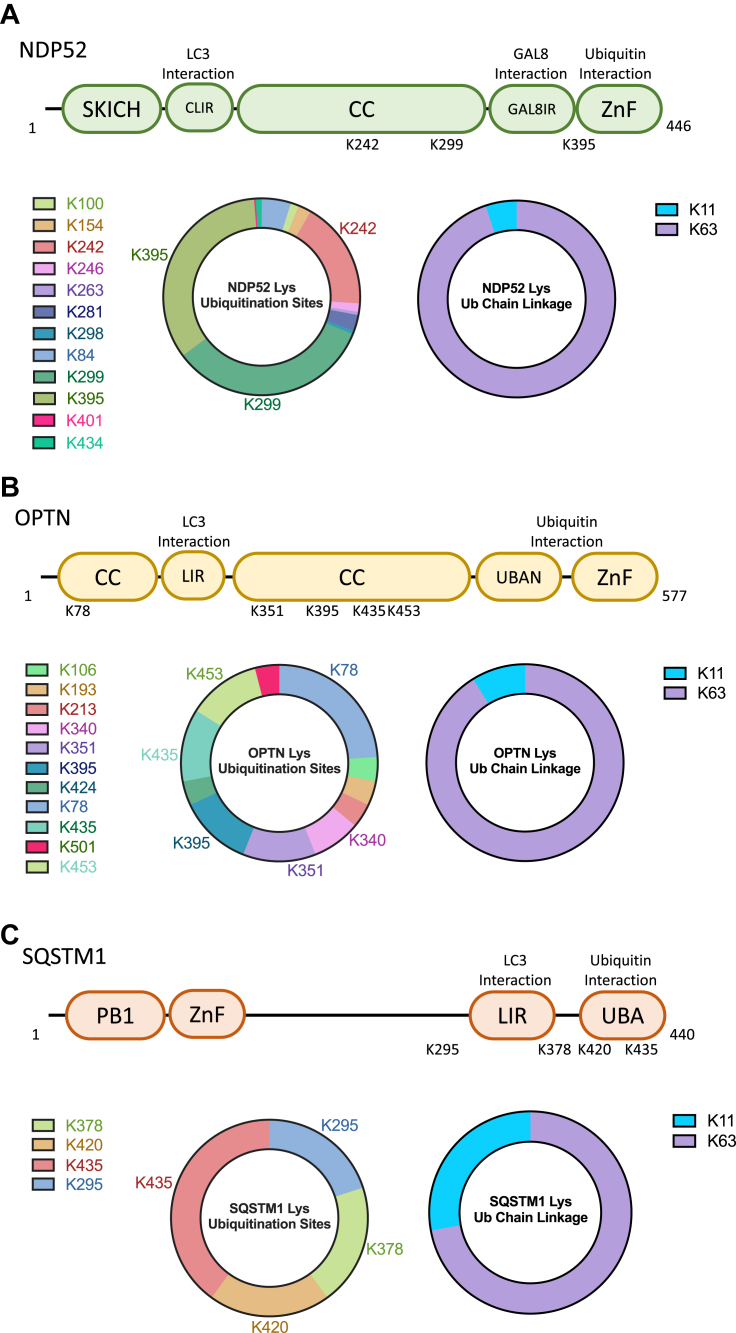
**Mapping the sites of ubiquitination in NDP52, OPTN, and SQSTM1 using tandem mass spectrometry.***A*, LC-MS/MS analysis of NDP52 ubiquitination by WWP2. The domain architecture of NDP52 is shown in *light green* in the cartoon. Three major lysine ubiquitination sites (K242, K299, K395) were identified. The major ubiquitin chain type was identified as K63 linkage. *B*, LC-MS/MS analysis of OPTN ubiquitination by WWP2. The domain architecture of OPTN is shown in *light yellow* in the cartoon. Six major lysine ubiquitination sites (K78, K340, K351, K395, K435, K453) were identified. The major ubiquitin chain type was identified as K63 linkage. *C*, LC-MS/MS analysis of SQSTM1 ubiquitination by WWP2 Y369E. The domain architecture of SQSTM1 is shown in *light orange* in the cartoon. Four lysine ubiquitination sites (K295, K378, K420, K435) were identified. The major ubiquitin chain type was identified as K63 linkage. OPTN, optineurin; SQSTM1, sequestosome.

Label-free tandem mass spectrometry was used to map the sites of ubiquitination in NDP52, OPTN, and SQSTM1 after WWP2^Y369E^ treatment (Fig. 6). A total of 12 Lys sites in NDP52 were found, with the three major sites identified as Lys242, Lys299, and Lys395 (Fig. 6*A*). These major NDP52 ubiquitination sites have been mapped by mass spectrometry in prior cellular studies (PhosphositePlus, www.phosphosite.org). In OPTN, there were 11 Lys sites that were ubiquitinated and six of these (Lys78, Lys340, Lys351, Lys395, Lys435, and Lys453) seemed to be more prevalent than the others (Fig. 6*B*). Previously published OPTN Ub sites include Lys78, Lys351, and Lys435 that were observed here (www.phosphosite.org). For SQSTM1, four sites were identified, Lys295, Lys378, Lys420, and Lys435 all on the C-terminal part of the protein (Fig. 6*C*). Each of these SQSTM1 sites has been reported previously (www.phosphosite.org) and Lys420 in SQSTM1’s UBA (ubiquitin association) domain was found to be functionally important (59). We also analyzed the Ub-Ub linkages in each of the WWP2 autophagy receptor reactions by mass spectrometry and found that Lys63 linkages predominated and Lys11 linkages were also observed (Fig. 6, *A*–*C*, Ub linkage panel). Lys63 Ub linkages have previously been shown to be the major polyubiquitination linkages in studies on WWP2 and other related NEDD4 family members (14, 60, 61). Taken together, WWP2’s Lys ubiquitination sites in the autophagy receptors show substantial concordance with known cellular ubiquitination sites in NDP52, OPTN, and SQSTM1 proteins, suggesting potential physiological significance to these WWP2-mediated Lys modifications.

### Cellular analysis of WWP2 and autophagy receptor in cotransfection experiments

**Figure 7:**
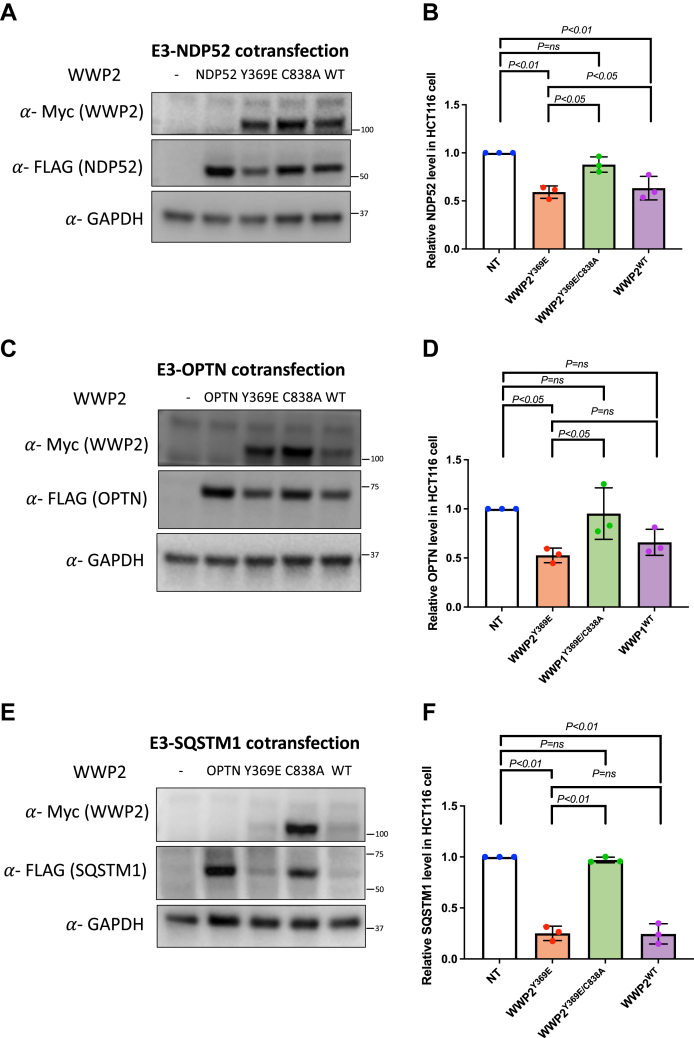
**Cellular analysis of WWP2 and autophagy receptors NDP52 and OPTN.***A*, immunoblotting of cells cotransfected with NDP52 and various forms of WWP2. *B*, statistical analysis of NDP52 and WWP2 cotransfection experiments (n = 3) using the one-way ANOVA model. *C*, immunoblotting of cells cotransfected with OPTN and various forms of WWP2. *D*, statistical analysis of OPTN and WWP2 cotransfection experiments (n = 3) using the one-way ANOVA model. *E*, immunoblotting of cells cotransfected with SQSTM1 and various forms of WWP2. *F*, statistical analysis of SQSTM1 and WWP2 cotransfection experiments (n = 3) using the one-way ANOVA model. NT, no transfection; Y369E, linker phosphorylation mimetic; C838A, contains the linker Y369E phosphorylation mimetic along with the catalytic Cys mutation; OPTN, optineurin; SQSTM1, sequestosome.

To examine the potential of WWP2 to ubiquitinate NDP52, OPTN, and SQSTM1 in cells, we cotransfected Myc-tagged WWP2 and the corresponding FLAG-tagged autophagy receptors in the HCT116 colorectal cancer cell line. We monitored the effects of WWP2^Y369E^, WWP2^Y369E/C838A^, and WWP2^WT^ on each of the autophagy receptor protein levels by Western Blot (Fig. 7). In these experiments, WWP2^Y369E^ transfection led to the reduced expression levels of NDP52, OPTN, and SQSTM1, whereas the catalytically dead mutant WWP2^Y369E/C838A^ showed no significant effect on the transfected autophagy receptor levels, indicative of WWP2 activity-dependent substrate degradation (Fig. 7, *A* and *B*, NDP52; Fig. 7, *C* and *D*, OPTN; Fig. 7, *E* and *F*, SQSTM1). WWP2^WT^ transfection generally showed a similar impact (NDP52, SQSTM1) or modestly weaker effect (OPTN) relative to WWP2^Y369E^ on the autophagy receptors suggesting that under these transfection conditions, WWP2 phosphorylation might not be critical for targeting these substrates.

**Figure 8:**
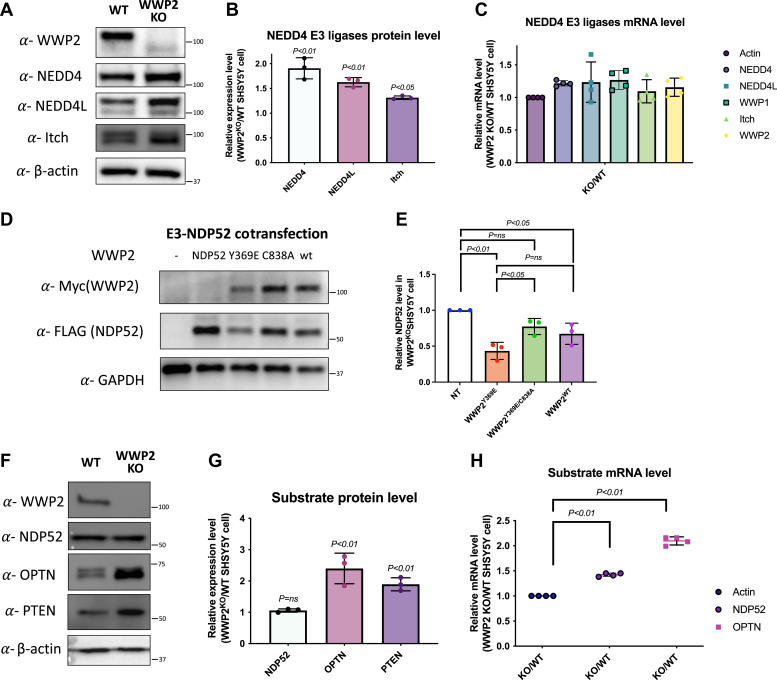
**Cellular study of WWP2 and autophagy receptors in CRISPR-Cas9 WWP2 KO SYSH5Y cells.***A*, immunoblotting analysis of the endogenous levels of WWP2 and other NEDD4 family E3 ligases comparing parental and WWP2^KO^ SHSY5Y cells. *B*, statistical analysis of NEDD4, NEDD4L, and Itch protein level in WWP2^KO^ cells (n = 3) using a paired *t* test. *C*, RT-qPCR analysis of the mRNA level of NEDD4 family E3 ligases, including NEDD4, NEDD4L, WWP1, ITCH, and WWP2. *D*, immunoblotting of cells cotransfected with NDP52 and various forms of WWP2 in WWP2^KO^ cells. *E*, statistical analysis of NDP52 and WWP2 cotransfection experiments in WWP2^KO^ cells (n = 3) using the one-way ANOVA model. *F*, immunoblotting analysis of the endogenous level of WWP2 and its substrates comparing parental and WWP2^KO^ SHSY5Y cells. *G*, statistical analysis of WWP2 substrates NDP52, OPTN, and PTEN protein level in WWP2^KO^ cells (n = 3) using paired *t* test. *H*, RT-qPCR analysis of mRNA level of autophagy receptors NDP52 and OPTN. OPTN, optineurin.

For additional cellular experiments, we used CRISPR-Cas9 to genetically delete WWP2 in neuroblastoma SH-SY5Y cells (62). These cells showed the absence of WWP2 by Western Blot, but interestingly, there were small increases in NEDD4, NEDDL, and Itch, perhaps as compensation for the loss of WWP2 (Fig. 8, *A* and *B*). We also analyzed mRNA levels of NEDD4, NEDD4L, WWP1, and Itch with RT-qPCR and found similar small increases. (Fig. 8*C*). Repeats of transient transfection with WWP2 and NDP52 in SH-SY5Y WWP2 KO cells revealed diminished NDP52 levels with WWP2^Y369E^ transfection but not catalytically inactive WWP2^Y369E/C838A^ transfection and an intermediate level in WWP2^WT^-transfected cells (Fig. 8, *D* and *E*). We looked further at the endogenous levels of PTEN, NDP52, and OPTN in parental SH-SY5Y cells compared with WWP2 KO SH-SY5Y cells (Fig. 8*F*). These studies showed that PTEN and OPTN protein levels were ∼2-fold elevated in WWP2 KO SH-SY5Y cells relative to the parental cells whereas NDP52 protein levels were unchanged (Fig. 8*G*). To understand further the NDP52 and OPTN protein results, we used RT-qPCR to quantify the mRNA transcript levels for these genes. It was observed that OPTN mRNA was ∼2-fold elevated in WWP2 KO SH-SY5Y cells relative to the parental cells, which presumably contributes to the ∼2-fold elevated protein level. There appeared to be only a small increase (∼40%) in NDP52 mRNA in the WWP2 KO SH-SY5Y cells (Fig. 8*H*). Therefore, it appears that WWP2 deletion from SH-SY5Y cells has little to no effect on regulating the endogenous protein levels of NDP52 and OPTN by targeted ubiquitination and degradation.

**Figure 9:**
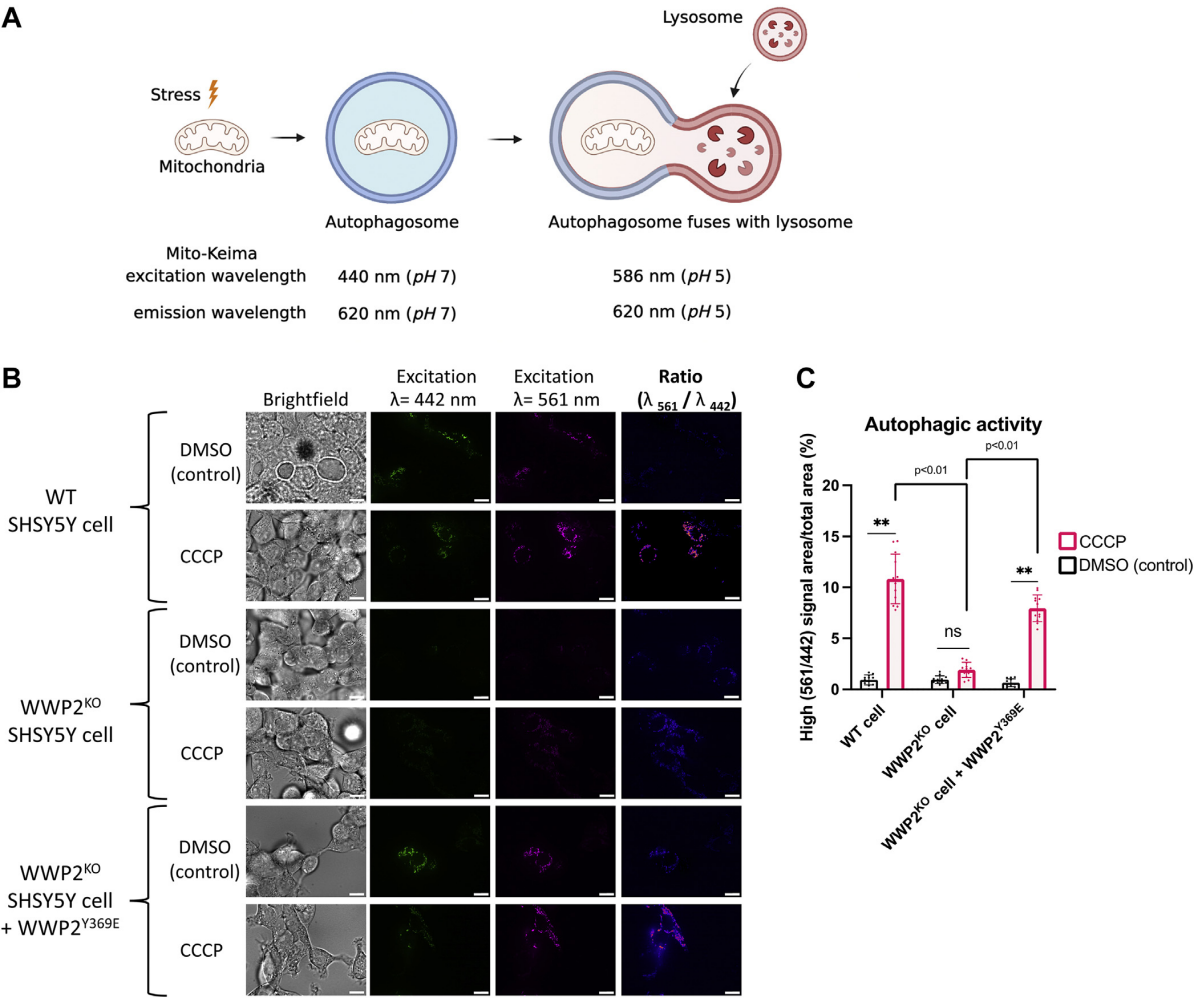
**Live-cell microscopy study of mitochondrial autophagy detection using Mito-Keima as the probe.***A*, schematic representation of mitophagy detection with the Mito-Keima probe. *B*, dual excitation ratiometric imaging using Mito-Keima signals in WT, WWP2^KO^ SHSY5Y, or WWP2^KO^ SHSY5Y cells transfected with WWP2^Y369E^. The brightfield, fluorescence excited at λ= 442 nm and fluorescence excited at λ= 586 nm images were taken, and representative images were shown. The ratiometric images were processed and were shown as the ratio of the 550 nm excitation wavelength signals (Mito-Keima in lysosome acidic environment) *versus* the 440 nm excitation wavelength signals (Mito-Keima in cytosol neutral environment). CCCP, a mitochondrial uncoupler, was used to induce mitophagy. The scale bar represents 10 μm. *C*, statistical analysis of mitochondrial autophagy activity based on Mito-Keima ratiometric signals in induced mitophagy. The high (561/445) signal area *versus* total area percentage was quantified. CCCP, carbonyl cyanide chlorophenylhydrazone.

We then investigated the propensity of WWP2 KO SH-SY5Y cells relative to the parental cells to undergo induced mitochondrial autophagy (mitophagy) in response to treatment with the small molecule protonophore, the mitochondrial decoupler carbonyl cyanide chlorophenylhydrazone (CCCP). We used the pH sensitive Mito-Keima fluorescent protein as a measure of mitophagy (63). The Mito-Keima reporter protein associates with the mitochondrial outer membrane and displays a distinct excitation wavelength in an acidic environment (pH 5, 586 nm), compared to a neutral environment (pH 7, 440 nm) (Fig. 9*A*). In parental SH-SY5Y cells, we found substantial mitophagy as evidenced by the high ratiometric 561 nm/442 nm fluorescent signal after 24 h of CCCP *versus* vehicle treatment (Fig. 9, *B* and *C*). However, in the WWP2 KO SH-SY5Y cells, the degree of mitophagy induced by CCCP was negligible (Fig. 9, *B* and *C*, WWP2^KO^ panel). Importantly, transient transfection of WWP2^Y369E^ into WWP2 KO SH-SY5Y cells could restore the mitophagy response to CCCP (Fig. 9, *B* and *C*). These results suggest that the presence of WWP2 contributes to the mitophagy response in these cells.

## Discussion

Identifying protein substrates of ubiquitin E3 ligases in genetic/cellular experiments is challenging because of the complex milieu of the cell, the hundreds of E3 ligases present, and the potential for many indirect effects associated with loss- or gain-of-function of a particular enzyme (64, 65). Protein microarray technology offers an attractive alternative to standard cellular experiments because its application can rapidly and directly assess proteome wide enzyme-substrate relationships, even for low abundance proteins (66, 67). Moreover, as HECT E3 ligases are self-contained catalysts compared to RING E3 ligases which serve adaptor functions, HECT E3s are particularly amenable to such *in vitro* experiments (30, 68, 69). Despite this, protein microarrays have seen limited use in HECT substrate detection (53, 70), perhaps because their basal activities can be low. Indeed, we found that WWP2^WT^ was nearly inert in ubiquitinating substrates on the protein microarrays. However, the use of the hyperactive phosphomimetic WWP2^Y369E^ simulated physiological conditions and allowed us to identify 31 possible ubiquitinated substrates, including the well-validated tumor suppressor PTEN.

Our attention was drawn to the three autophagy receptors, NDP52, OPTN, and SQSTM1, which represented about 10% of the positive hits in our WWP2^Y369E^ ubiquitination screen. Further biochemical analysis indicated that they were superior solution phase WWP2 substrates compared to the established substrate PTEN and subject to the same 2,3-linker and allosteric activator molecules that can enhance PTEN ubiquitination (12, 14). To our knowledge, only SQSTM1 of these autophagy receptors has been reported to be a substrate of the NEDD4 family of enzymes (43, 44).

Mass spectrometry led to the identification of numerous ubiquitination sites of WWP2^Y369E^ among NDP52, OPTN, and SQSTM1. These ubiquitination sites were observed to be relatively widespread across each protein, suggesting that conformational plasticity in the E3-substrate interactions is involved in Lys modification. A relatively large subset of these ubiquitination sites has been mapped in previously reported proteomics studies on the endogenous autophagy receptors isolated from cells, although the function of these ubiquitination events are largely unexplored. Lys193 of OPTN has been reported to be ubiquitinated by the E3 ligase HACE1 and this is thought to promote OPTN’s interaction with SQSTM1 (71). Our data suggest that Lys193 of OPTN may also be ubiquitinated by WWP2, perhaps indicating an alternative mechanism for this autophagy-related function. In addition, Lys7 of SQSTM1 has been reported to be ubiquitinated by the E3 ligase TRIM21, which impairs its oligomerization and suppresses its sequestration function (72). SQSTM1 Lys7 has also been found to be ubiquitinated by NEDD4, which is proposed to regulate SQSTM1’s conformation (44). Also, our mass spectrometry data suggest that K63-linked polyubiquitination is the major polyubiquitination chain type for NDP52, OPTN, and SQSTM1. In contrast to K48-linked polyubiquitination, which leads to protein proteasomal degradation, K63-linked polyubiquitination has been reported to be more important in nonproteolytic cellular processes, such as endocytosis, protein trafficking, and the innate immune response (73).

The WWP2 knockout experiments in SH-SY5Y cells reveal a role for this enzyme in mitophagy, a form of autophagy that is widely believed to depend on the NDP52, OPTN, and SQSTM1 proteins (41, 74). Although the co-overexpression of WWP2^Y369E^ and these autophagy receptors suggests that ubiquitination of these proteins can reduce their cellular levels, possibly through polyubiquitination-mediated protein degradation, such changes were not seen with endogenous NDP52 in the WWP2 KO cells. RT-qPCR results with OPTN suggest that its level is increased transcriptionally by chronic WWP2 depletion instead of by an acute posttranslational change expected for a direct ubiquitination effect. Indeed, using Mito-Keima as a probe for induced mitophagy, we found that WWP2 seems to be critical for the mitochondrial autophagic signaling in cells.

Moreover, transient transfection of WWP2^Y369E^ could restore the normal mitophagy induction in WWP2^KO^ cells. NDP52 and OPTN have been demonstrated to be important receptors for PINK1 and parkin-mediated mitophagy (75). These autophagy receptors can be recruited to mitochondria and associate with LC3-coated phagophores for autophagosome formation and their fusion with lysosomes (76, 77). Interestingly, we observed in our previous study (12) that activated WWP2 colocalizes with LAMP-1, a lysosome marker. This colocalization is abolished by mutation of the catalytic Cys in WWP2. These findings and our general understanding of NDP52, OPTN, and SQSTM1 in autophagy suggest that WWP2-mediated K63-linked ubiquitination of the autophagy receptors under physiological conditions would be unlikely to lead to the degradation of these autophagy receptors. Rather, as proposed for NEDD4-mediated ubiquitination of SQSTM1 (43, 44), WWP2-mediated ubiquitination of NDP52, OPTN, or SQSTM1 might lead to the conformational changes in these autophagy receptors or modulate protein–protein interactions for the recruitment of downstream effectors that positively contribute to the mitophagy process. Future studies should be aimed at uncovering the detailed molecular mechanisms about how WWP2 regulates these autophagy receptor functions.

## Experimental procedures

### Plasmids and cloning

The proteins expressed were based on human sequences unless otherwise noted. The pGEX6-p-2 WWP2 plasmid was gifted by Dr Wenyi Wei at Beth Israel Deaconess Medical Center. pDEST-GST human NDP52, OPTN, and p62 were provided by Dr Wade Harper at Harvard Medical School. pFlag-CMV2-WBP2 was provided by Dr Marius Sudol (Addgene plasmid # 27478). Ndfip1 cDNA, WBP2 cDNA, and UbV (ubv P2.3) cDNA were synthesized by Integrated DNA Technologies. The pET3a ubiquitin plasmid was obtained from Dr Cynthia Wolberger at Johns Hopkins University. The mKeima-Red-Mito-7 plasmid was made available to us from Dr Michael Davidson (Addgene plasmid # 56018). Mutations and truncations of WWP2 were cloned and introduced by Quik-Change (Agilent protocol) or restriction enzyme-based cloning. Vector swapping was performed using Gibson Assembly Master Mix (New England Biolabs). Constructs that were used for recombinant protein expression or cell transfection were confirmed by DNA sequencing of the full ORFs. DNA primers were obtained from Integrated DNA or QuintaraBio.

### Recombinant protein expression and purification

pGEX 6p-2 WWP2WT and mutant forms, pDEST-NDP52, OPTN, SQSTM1, pGEX 6p-2 ubiquitin variant were transformed in BL-21 (DE3)Codon^+^ or BL21 Rosetta (DE3)pLysS competent cells for *E. coli* recombinant protein expression. The transformed cells were cultured from fresh LB plates into LB medium at 37 °C to optimal cell density at A_600_ = 0.6. 0.25 to 0.5 mM IPTG was added to induce protein expression at 16 °C for 20 h. The cells were then harvested by centrifugation and resuspended in a lysis buffer containing 25 mM Hepes (hydroxyethyl-piperazine-ethylsulfonate) *pH* 7.8, 250 mM NaCl, 1 mM tris-carboxyethyl-phosphine (TCEP), 1 mM PMSF, and 1X Pierce EDTA-free protease Inhibitor cocktail (#A32965, Thermo Fisher). Resuspended cells were lysed by french press, and the supernatant was loaded on to a glutathione agarose column for GST-tag protein binding. The resultant resin-bound mixture was washed with 25 mM Hepes *pH* 7.8, 250 mM NaCl, 1 mM TCEP, and 0.1% Triton X-100, and then the desired GST-tagged protein was eluted with 12 ml lysis buffer with 50 mM reduced glutathione. The eluted protein were then treated with TEV or Prescission protease (GE) overnight to remove the GST tag in a dialysis cassette against buffer of 25 mM Hepes *pH* 7.8, 250 mM NaCl, 1 mM TCEP. The cleavage mixture was reloaded on to a glutathione agarose column to remove the cleaved GST tag. For WWP2 and UbV, these proteins were further purified with a Superdex 200 increase 10/300 Gl or Superdex 75 10/300 Gl size exclusion column (Cytiva/GE). SQSTM1 was purified as the GST-tagged form with a MonoQ 5/50 Gl anion exchange column after glutathione column purification as removing the GST tag dramatically destabilized the protein.

pET28b-Ndfip1 (aa. 1–114, N-terminal region) and pET28b-WBP2 (aa. 149–261) were transformed to Rosetta pLysS *E. coli* cells and cultured as described previously (14). In brief, the protein expression was done similarly as described above for WWP2 protein. The cells were harvested by centrifugation and resuspended in a lysis buffer containing 50 mM Hepes *pH* 7.8, 500 mM NaCl, 1 mM TCEP, 1 mM PMSF, and 1X Pierce EDTA-free protease Inhibitor cocktail (#A32965, Thermo Fisher). Resuspended cells were lysed by french press, and the supernatant was loaded onto a Ni^+^ NTA fast flow resin for Histidine-tag protein binding. Followed by washing with lysis buffer, the desired His-tagged protein was eluted with imidazole and further purified with a Superdex 75 10/300 Gl (Cytiva/GE) size exclusion column. The fractions were analyzed by SDS-PAGE gel and fractions containing the Ndfip1 or WBP2 protein were combined, concentrated, and flash-frozen to be used in the biochemical assays.

### Protein microarray substrate screening

HuProt (v3.2, Aug 20th, 2018) protein microarrays were directly transferred from a −80 °C freezer to a buffer PBS buffer containing 0.1% Tween 20 and 5% bovine serum albumin (BSA) for blocking at room temperature for 1 h on an orbital shaker. After blocking, the microarrays were rinsed three times with 5 ml reaction buffer containing 40 mM Tris–HCl *pH* 7.5, 50 mM NaCl, 5 mM MgCl_2_, 0.5 mM TCEP for 5 min each time. The reaction buffer was carefully removed between the washes using a 5 ml pipette. The ubiquitination reaction was first prepared in an Eppendorf tube using reaction buffer with 1 μM E1, 5 μM E2, 1 μM E3 (wt-WWP2 or Y369E phospho-mimetic WWP2, no E3 in the negative control group), and 100 μM wt ubiquitin. ATP (5 mM final concentration) was added to the ubiquitination reaction to initiate the reaction, and the reaction solution was immediately transferred onto the prewashed protein microarrays to ubiquitinate proteins on the microarray and covered with glass coverslip (Thermo Fisher, #25X601-2-4789). The ubiquitination reaction was performed at 30 °C on HuProt protein microarrays for 90 min in a temperature-controlled incubator without shaking. After 90 min, the coverslip was removed and a quenching buffer of 40 mM Tris–HCl *pH* 7.5, 500 mM NaCl, 0.5 mM TCEP, and 100 mM EDTA was used to wash the microarrays for 10 min three times at room temperature on an orbital shaker. The microarrays were further washed for 10 min three times with TBST with 1% SDS, and then washed four times for 15 min each with PBS at room temperature. Then, the microarrays were incubated with anti-ubiquitin antibody (1:500, #sc-8017, Santa Cruz) in a PBS buffer containing 5% BSA at 4 °C overnight. After primary antibody incubation, the antibody solution was removed and the microarrays were washed with TBST four times for 15 min each. A secondary anti-mouse Alexa 555 Fluor antibody (1:5000, #21422, Thermo Fisher) was used to incubate the microarrays for 1.5 h at room temperature. The microarrays were washed with TBST for 15 min four times and then highly purified water three times for 15 min each. The washing solution was removed from the microarray, and then, brief centrifugation at 1000 rpm for 2 min was performed to remove most of the liquid. The microarrays were then allowed to air dry, avoiding light. The microarrays were then scanned using a GenePix scanner, (4200) and the fluorescence intensities were quantified using a grid file for HuProt (v3.2) and GenePix Pro software.

### Identification of ubiquitination substrate using HuProt microarray

Using the GenePix scanner, the signal intensity (R_ij_) of a given protein spot on HuProt microarray was generated using the median foreground signal (F_ij_) minus the median background signal (B_ij_). The average R_ij_ from duplicate spots was used and defined as the protein probe R_p_. The Z-score (Z_p_) of each protein probe R_p_ in on-microarray ubiquitination was computed with the distribution of R_p_ using the equation below,

$$Z_{p}=\frac{R_{p}-N}{SD}$$

where SD and N represent the SD and the mean of the noise distribution on the protein microarrays, separately.

A cutoff of Z ≥ 3 was used to determine the positive hits from each protein microarray. Duplicates of each protein microarray were run, and positive hits were based on reproducible appearance on both hits and further verified and validated with the visual inspection of the chips. The hits that also appear on the control microarrays (lacking WWP2) were removed from further analysis as they are assumed to be preubiquitinated or underwent self-ubiquitination (with the E2) without requiring the presence of WWP2 protein. The GO enrichment analysis of the 31 identified hits from WWP2^Y369E^ chips was performed by referencing the human whole proteome database (http://geneontology.org/). The GO cellular component and biological process enrichment of these were visualized using the GOrilla and REVIGO tools. For GO cellular component analysis, enrichment scores were calculated with GOrilla (http://cbl-gorilla.cs.technion.ac.il/) and the graph was plotted with GraphPad Prism 9. For GO biological process analysis, the enrichment was done by GOrilla and visualized using the REVIGO online tool (http://revigo.irb.hr/).

### *In vitro* ubiquitination

The *in vitro* ubiquitination reactions were performed in microcentrifuge tubes at a final volume of 20 μl as described previously (14). The reaction buffer contains 40 mM Tris–HCl pH 7.5, 50 mM NaCl, 0.5 mM TCEP and 5 mM MgCl_2_ and 5 mM ATP. Ubiquitin (50 μM), E1 enzyme (50 nM), E2 enzyme(UbcH5b, 1 μM), and 1 μM WWP2 (WT or Y369E or Y392E) were used in the assays. Protein substrates (NDP52, OPTN, or SQSTM1) were present at a final concentration of 5 μM for NDP52 and OPTN, and 2 μM for SQSTM1. In brief, initially, the reaction components were added and mixed well except for the E1 protein was left out. The ubiquitination reactions were initiated by adding E1 protein to the system and the reactions were carried out at 30 °C. At the indicated time points (15, 30, 60 min), the reactions were quenched by adding SDS-loading dye containing BME as the reducing agent. Then, the samples were separated by SDS-PAGE gel and visualized using a Colloidal Blue Staining kit (Thermo Fisher). The gel images were analyzed using a G:Box mini 6 imaging system (Syngene). The depletion of the unmodified substrate proteins was determined by densitometry analysis with Image J (version 1.53a) and presented as a percentage compared to the time zero sample.

### N-terminal fluorescent labeling of WWP2 and WBP2

WWP2 expression was the same as mentioned in the previous section, but the purification for N terminal Cys WWP2 was slightly different (58). Briefly, after binding with glutathione agarose and washing with 25 mM Hepes pH 7.8, 250 mM NaCl, and 0.1% Triton X-100, the GST-tagged WWP2 was eluted with 50 mM reduced glutathione in buffer containing 25 mM Hepes pH 7.8, 250 mM NaCl, and 1 mM TCEP. The eluted fractions were treated with TEV protease to remove GST and expose the N-terminal Cys residue and then, the samples were dialyzed twice into buffer containing 25 mM Hepes pH 7.8, 250 mM NaCl, and 1 mM TCEP at 4 °C. The mixture was then passed through Ni^+^ NTA resin and glutathione agarose to remove the TEV protease and free GST-tag, respectively. The N-Cys WWP2 was further dialyzed in to a buffer with 100 mM Hepes pH 7.0, 150 mM NaCl, and 1 mM TCEP overnight at 4 °C and labeled with Cy5 thioester, which was generated by incubating Cy5 NHS ester ((#43320, Lumiprobe) with 500 mM MESNa, at room temperature for 24 h. After the labeling, the protein was dialyzed in to 50 mM Hepes pH 7.8, 150 mM NaCl, 1 mM TCEP for 16 h at 4 °C to remove excess dye. Next, the Cy5 fluorescent-labeled WWP2 was further purified by size-exclusion chromatography with a Superdex 200 increased 10/300 Gl column (Cytiva/GE). Purified fractions were combined, concentrated, and stored at −80 °C. WBP2 labeling was done with a similar procedure as that used for WWP2 but labeled with fluorescein (5/6-carboxyfluorescein) NHS ester (#46410, Thermo Fisher) instead of Cy5. The final FPLC purification of fluorescein-labeled WBP2 was conducted using a Superdex 75 10/300 Gl column (Cytiva/GE). The WBP2 fractions were collected, combined, concentrated, and stored at −80 °C.

### MST procedure

As described previously (56), MST was used to evaluate the binding affinity *K*_D_ between WWP2 and autophagy receptors NDP52 and OPTN. In brief, the N-terminal Cy5-labeled WWP2 at a final concentration of 10 nM was used in the MST assays, with a buffer containing 25 mM Hepes *pH* 7.8, 250 mM NaCl, 1 mM TCEP, 0.5 mg/ml BSA, and 0.05% Tween-20. NDP52 or OPTN ligands were prepared using a two-fold serial dilution with the highest concentration of NDP52 at 48 μM or OPTN at 60 μM. WWP2 and its substrate were mixed and incubated at room temperature for 10 min for equilibration before transferring to MST capillaries. The MST assays were performed with a Monolith NT.115 (NanoTemper) using the Pico-Red mode. The laser power was set at 20% and MST power was set to Medium. The assays were repeated at least twice on different occasions and the *K*_D_ was calculated using Mo.analysis (v3.2) software with a quadratic equation binding *K*_D_ model.

### Fluorescence anisotropy

Fluorescein-labeled WBP2 at 100 nM was mixed with an indicated series of WWP2 protein and incubated at room temperature for 20 min to allow for equilibration. Then, the binding samples were transferred to 96-well plate (#267342, Corning) and steady-state fluorescence anisotropy was measured using Cytation 5 plate reader (BioTek) at 23 °C with the BioTek fluorescein filter set (excitation wavelength at 485 nm and emission wavelength at 528 nm). Fluorescence anisotropy data were measured to high accuracy at least 3 times. Using GraphPad Prism 9, the binding data was plotted and *K*_D_ values were calculated based on the quadratic equation binding fit model:

Y = Y_0_ –[(Y_0_ – Y_max_)/(2∗Fixed)]∗[b-sqrt(bˆ2-4∗x∗Fixed)], where b = *K*_D_ + x + Fixed (Fixed = 0.1). At least, two independent replicates were carried out on independent occasions with measured *K*_D_ values in good agreement (within 30%).

The NDP52-WBP2 competitive binding assay was conducted following similar procedures. Instead of varying the WWP2 concentration, however, a fixed concentration of WWP2 (5.7 μM, 1.5-fold of the WBP2-WWP2 *K*_D_ value) and varying concentrations of NDP52 were incubated with fluorescent-labeled WBP2. The fluorescence anisotropy values were measured and recorded as described above. The same quadratic binding fit model was used to evaluate the NDP52 competitive binding, but the apparent *K*_D_ was too high to be calculated.

### LC-MS/MS analysis for lysine ubiquitination sites on autophagy receptors

*In vitro* ubiquitinations were performed as described above for 60 min, and the samples were quenched with SDS loading dye and separated using SDS-PAGE. The gel bands were first visualized using colloidal blue staining, and the gel portions including ubiquitinated substrate bands or the related ‘smears’ were cut and collected. The cut bands were further cropped into 1 mm X 1 mm pieces, followed by performing dehydration with methanol for 5 min. To remove the gel stain, the excised gel pieces were first washed with 30% methanol in water for 5 min, then washed with water for 10 min, two times each, and then with 100 mM ammonium bicarbonate in 30% acetonitrile for 10 min, three times each. After washing, the gel pieces were dried using a SpeedVac and treated with reducing agent TCEP in 100 mM NH_4_HCO_3_ for 60 min at 55 °C, followed by treated with 50 mM 2-chloroacetamide in 100 mM NH_4_HCO_3_ for 45 min at room temperature avoiding light. The gel pieces were washed again with 100 mM NH_4_HCO_3_ for 15 min and dehydrated with acetonitrile. After dehydration, the gel pieces were completely dried using a SpeedVac and rehydrated with 50 mM NH_4_HCO_3_ solution containing 20 ng/μl trypsin for digestion overnight at 37 °C. After digestion, the mixtures were collected and the gel pieces were washed with 50 mM NH_4_HCO_3_, acetonitrile, 5% formic acid in 50% acetonitrile (x2). All the washing solutions were collected and combined with the digestion solution and dried by a SpeedVac. The samples were reconstituted in 50 μl of 0.2% TFA and desalted using C18 STAGE tips (78).

Liquid chromatography was performed with a 75 μm × 15 cm Acclaim PepMap 100 separating column on a Dionex Ultimate 3000 RSLCnano system (Thermo Scientific). The mobile phase was 0.1% formic acid in water (A) and 0.1% formic acid in 95% acetonitrile (B) with a flow rate of 300 nl/min. MS analysis was performed on an LTQ Orbitrap Velos Pro mass spectrometer (Thermo Scientific). The spray voltage was set at 2.2 kV and the Orbitrap spectra were collected from m/z 400 to 1800 at a resolution of 30,000, followed by data-dependent HCD MS/MS, which has a resolution of 7500 with a collision energy of 35% and an activation time of 0.1 ms, of the 10 most abundant ions using 2.0 Da isolation width. Charge-state screening was enabled to reject the generation MS/MS spectra from unassigned and singly charged precursor ions. A dynamic exclusion time of 40 s was used to discriminate against previously selected ions. An in-house software pipeline (Feb, 2014) was used to search the data against the Uniprot human proteome (88,591 entries). Two missed and/or nonspecific cleavages were permitted during the search. Database search parameters were as follows: enzyme, trypsin; precursor mass tolerance, 10 ppm; fragment ion tolerance, 0.03 Da; static modification, Cys carbamidomethylation; variable modifications, Met oxidation, Lys ubiquitination (di-Gly modification) and acetylation, and Ser/Thr/Tyr phosphorylation. Reversed sequences of all proteins were appended to the search database for the target-decoy false discovery rate analysis (79, 80). The data were filtered using a 0.02 false discovery rate threshold and a maximum peptide rank of 1. All MS/MS spectra assigned to modified NDP52, OPTN, SQSTM1, or ubiquitin peptides were manually inspected. The relative abundances of the ubiquitin chain linkages were determined using area under the curve or spectral counts.

### Cell culture and cellular transfection experiments

The HCT116 colon cancer cell line was obtained from ATCC and cultured with McCoy’s 5A medium supplemented with 10% fetal bovine serum, L-Glutamine and penicillin/streptomycin in a 37 °C incubator with 5% CO_2_. SH-SY5Y neuroblastoma cells was obtained from Dr Mark Grimes at the University of Montana and cultured with DMEM medium with 10% fetal bovine serum, L-Glutamine and penicillin/streptomycin in a 37 °C incubator with 5% CO_2_. For transient transfection experiments, the HCT116 or SH-SY5Y cells were seeded in a 6-well plate and transfected with plasmid using Lipofectamine 3000 reagent (Thermo Fisher) at 90% confluence. 0.6 μg OPTN or 0.6 μg NDP52 or 0.2 μg SQSTM1 plasmids was transfected with or without different forms of 0.6 μg WWP2 (wt, Y369E, or Y369E + C838S) and 0.5 μg ubiquitin. After 24 to 48 h of transfection, the cells were lysed with RIPA buffer (Cell signaling) containing 1X Pierce protein inhibitor cocktail tablets (EDTA free, Thermo Fisher). The supernatants were collected and the total protein concentrations were measured using with a BCA protein assay kit (#23225, Thermo Fisher).

### CRISPR-Cas9 knockout of WWP2 gene in SH-SY5Y cells

The CRISPR-Cas9 knockout procedure was performed according to protocols previously reported (81). Briefly, guide RNAs were designed using Benchling (https://benchling.com), and two gRNA were selected with high on-target and off-target scores, targeting Exon 2 (5′-TCTGCCAGCTCTAGCCGGGC-3′) and Exon 3 (5′-ACCTCGAATTAACTCCTACG-3′) were used. The constructs pSpCas9(BB)-2A-Puro-v2 containing gRNA sequence together with sgRNA and Cas9 protein were cloned. SH-SY5Y neuroblastoma cells were cultured in 6-well plates until 70 to 90% confluency and electroporation-mediated transfection of two plasmids (0.75 μg) was conducted with a Lonza cell line Nucleofector kit V(10 RCT, #VACA1003). Twenty four hours after transfection, the cells were cultured in the presence of 1 μg/ml puromycin for 48 h for selection. Then, the cells were seeded into 96-well plates using serial dilutions to obtain single colony clones. After 3 weeks of growth, single colony clones were expanded and collected. The WWP2 KO colonies were collected and verified by Western blot using WWP2 antibody (#A302–935A, Bethyl Laboratories).

### Western blotting

The cell lysate samples were mixed with SDS loading dye and boiled for 5 min on a metal bath. Twenty micrograms of total protein was loaded and resolved using SDS-PAGE gels. The proteins were transferred to a nitrocellulose membrane using an iBlot dry blotting system (Thermo Fisher), and the membrane was blocked with 5% BSA in PBST buffer for 1 h before the incubation with different primary antibodies at 4 °C overnight. The antibodies used in this paper are listed below. Anti-ubiquitin mouse monoclonal antibody (P4D1, #sc-8017), anti-β-actin (#sc-47778) mouse mAb, anti-PTEN (A2B1, #sc-7974) mouse mAb were purchased from Santa Cruz. Goat anti-mouse Alexa Fluor 555 antibody (#21422) was from Thermo Fisher. Anti-Myc tag rabbit polyclonal antibody (#60003-2-lg) was purchased from Proteintech. Anti-Flag (D6W5B, #14793) rabbit mAb, anti-GAPDH (14C10, #2118S) rabbit mAb, anti-NDP52 (#9036) rabbit pAb were obtained from Cell Signaling. Anti-OPTN (#ab23666) rabbit pAb was purchased from Abcam. Anti-WWP2 (# A302–935A) rabbit pAb was obtained from Bethyl Laboratories. After overnight incubation, the membranes were washed with PBST and probed with horseradish peroxidase–conjugated secondary antibodies. The bands were detected by chemiluminescence using the clarity western ECL substrate (#1705060, Bio-rad) and imaged with a Syngene G:Box mini 6 imager (GeneSyns). Western blots were repeated at least three times, and the protein bands were quantified using ImageJ, normalized to GAPDH internal controls, and the error bar represents the standard error of the mean. The statistical significances and *p*-value were calculated using Graphpad Prism using either a paired *t* test or a one-way ANOVA model.

### RT-qPCR RNA level detection

Cells were washed twice with prechilled PBS and harvested with TRIzol reagent (Invitrogen). RNA samples were isolated using phenol-chloroform extraction and followed by ethanol precipitation. RT-qPCR was conducted with qScript SYBR One-step RT-PCR master mix (QuantaBio) on a Biorad CFX C1000 thermocycler with the primers listed below. The Biorad CFX manager (version 3.1) was used to analyze the data. RNA fold changes for autophagy receptors and other NEDD4 family E3 ligases were normalized to actin. The sequence of primers were ordered from Integrated DNA (USA) and are listed as follows:

NDP52: Forward: 5′-CCAGAAGCAGAACTCAGACAT-3′,

Reverse: 5′-TCTGTCTCCCAATAGTCCTTCT-3′;

OPTN: Forward: 5′-GCGAGAACAAGTGACTCTGAC-3′,

Reverse: 5′-GCAGAACCTCTCCACACTTG-3′;

SQSTM1: Forward: 5′-CTGCCTTGTACCCACATCTC-3′,

Reverse: 5′-CCGATGTCATAGTTCTTGGTCTG-3′;

β-Actin: Forward: 5′-ACAGAGCCTCGCCTTTG-3′,

Reverse: 5′-CCTTGCACATGCCGGAG-3′;

NEDD4: Forward: 5′-AAATTCAGCCGTGAGCCA-3′,

Reverse: 5′-GGTAATCCAGATGAAGTAGGCA-3′;

NEDD4L: Forward: 5′-GACCATCTGCATCTAGACCTG-3′,

Reverse: 5′-CATCCGCTACGTACAATGAAAT-3′;

Itch: Forward: 5′-GACCAGAACCTCTACCTCCT-3′,

Reverse: 5′-CTGCCATTGTTCATAGTTCCG-3′;

WWP1: Forward: 5′-GCAGCTCATCTCCAACCATAG-3′,

Reverse: 5′-CTATTCCATTCGTGCCTTCAAC-3′;

WWP2: Forward: 5′-TGGAAGGCGGAAGTAGGA-3′,

Reverse: 5′-GTGAAGCTGGTGGAAGAGAAG-3′;

### Live-cell microscopy for monitoring induced mitophagy

SH-SY5Y wt or WWP2^KO^ cells were grown on No. 1.5 coverslips (MatTek) to around 90% confluence and transfected with 0.5 μg of Mito-Keima-Red (for the E3 rescue group, with 0.6 μg WWP2^Y369E^) using Lipofectamine 3000. After 48 h of transfection, the cells were treated with DMSO or 30 μM CCCP (#C2729, Sigma Aldrich) to induce mitophagy for 24 h. Then, the cells were then mounted in a stage-top heated chamber warmed to 37 °C with 5% CO2 for live-cell imaging. DMEM media without phenol red were used during the image acquisition process to minimize background noise. All images were collected with an inverted Nikon Ti inverted fluorescence microscope equipped with Yokagawa CSU-X1 spinning disk confocal with Perfect Focus System. Mito-Keima fluorescence was excited with LMM-5 laser merge module with solid state laser at the wavelength of 445 nm (60 mW) or 561 nm (95 mW) and collected the emission fluorescence at 620 nm wavelength with 100 ms exposure time across the experiments. Images were acquired with Hamamatsu ORCA-R_2_ cooled CCD confocal camera with MetaMorph software. 13 z-series optical sections were collected with a step-size of 0.25 μm for a total 3 μm. The exposure time was set to 100 ms. Brightness, contrast, and gamma were adjusted for compared image sets.

Calculation of mitophagy based on the Mito-Keima signal was performed on a pixel-to-pixel basis. The fluorescence ratiometric images of 561/620 nm (represented in Margenta) *versus* 445/620 nm (represented in Green) were calculated and the particles were quantified using Fiji (vision 2.1.0/1.53c) with programmed micro to assure all images were processed the same. The autophagic signals were quantified as the percentage of high (561/445) signal area *versus* total area. The signal intensities were plotted and analyzed using GraphPad Prism 9. The significance and *p*-value were calculated using a Paired *t* test or one-way ANOVA model.

## Data availability

The mass spectrometry proteomics data have been deposited to the ProteomeXchange Consortium *via* the PRIDE (82) partner repository with the dataset identifier PXD031703 and 10.6019/PXD031703.

## Supporting information

This article contains supporting information that demonstrates all the assigned ubiquitinated peptide sequences and individual MS/MS spectra.

## Conflict of interest

The authors declare the following competing financial interest(s): Heng Zhu is a cofounder and equity holder in the company CDI, which fabricated the protein microarrays used here. Philip A. Cole has been a consultant for Scorpion Therapeutics. All other authors declare that they have no conflicts of interest with the contents of this article.
