# Supplementary material for: Enzymatic analysis of WWP2 E3 ubiquitin ligase using protein microarrays identifies autophagy-related substrates
Source: J Biol Chem. 2022 Mar 21;298(5):101854. doi: 10.1016/j.jbc.2022.101854 (PMC9034101; doi:10.1016/j.jbc.2022.101854)
Supplement: Supplementary material [file mmc2.pdf]

NDP52/CALCOCO2 K84

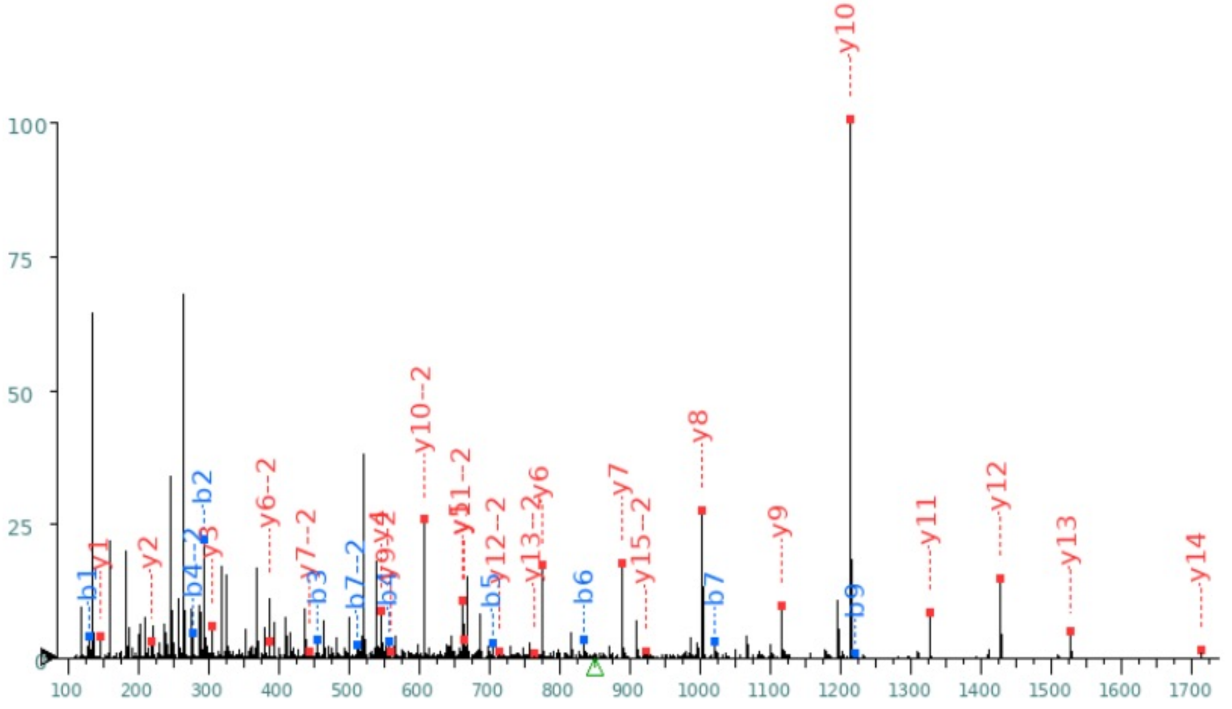

Sequence

EYYTFMWVTLPIDLNNK#SAK

Predicted Fragmentation Pattern

+1

Seq	#	b: Δ Error	b	y	y: Δ Error	+1
E	1	115.296	**130.050**	---	---	20
Y	2	-2.838	**293.113**	2418.216	---	19
Y	3	-9.862	**456.177**	2255.153	---	18
T	4	5.436	**557.224**	2092.090	---	17
F	5	-4.443	**704.293**	1991.042	---	16
M	6	-9.759	**835.333**	1843.973	---	15
W	7	-3.169	**1021.412**	**1712.933**	-2.787	14
V	8	---	1120.481	**1526.854**	-2.748	13
T	9	-0.754	**1221.529**	**1427.785**	-1.191	12
L	10	---	1334.613	**1326.738**	-1.964	11
P	11	---	1431.665	**1213.654**	-1.176	10
I	12	---	1544.749	**1116.601**	-1.689	9
D	13	---	1659.776	**1003.517**	6.167	8
L	14	---	1772.860	**888.490**	-2.347	7
N	15	---	1886.903	**775.406**	-2.193	6
N	16	---	2000.946	**661.363**	4.011	5
K#	17	---	2243.084	**547.320**	-1.585	4
S	18	---	2330.116	**305.182**	-2.203	3
A	19	---	2401.153	**218.150**	-1.402	2
K	20	---	---	**147.113**	-1.634	1

+2

Seq	#	b: Δ Error	b	y	y: Δ Error	+1
E	1	---	65.529	---	---	20
Y	2	---	147.060	1209.612	---	19
Y	3	---	228.592	1128.080	---	18
T	4	-1.047	**279.116**	1046.548	---	17
F	5	---	352.650	996.025	---	16
M	6	---	418.170	**922.490**	-14.382	15
W	7	13.000	**511.210**	856.970	---	14
V	8	---	560.744	**763.930**	0.960	13
T	9	---	611.268	**714.396**	-17.560	12
L	10	---	667.810	**663.872**	-2.937	11
P	11	---	716.336	**607.330**	-2.241	10
I	12	---	772.878	**558.804**	-1.317	9
D	13	---	830.392	502.262	---	8
L	14	---	886.934	**444.749**	-0.506	7
N	15	---	943.955	**388.206**	-4.015	6
N	16	---	1000.977	331.185	---	5
K#	17	---	1122.046	274.164	---	4
S	18	---	1165.562	153.095	---	3
A	19	---	1201.080	109.579	---	2
K	20	---	---	74.060	---	1

NDP52/CALCOCO2 K100

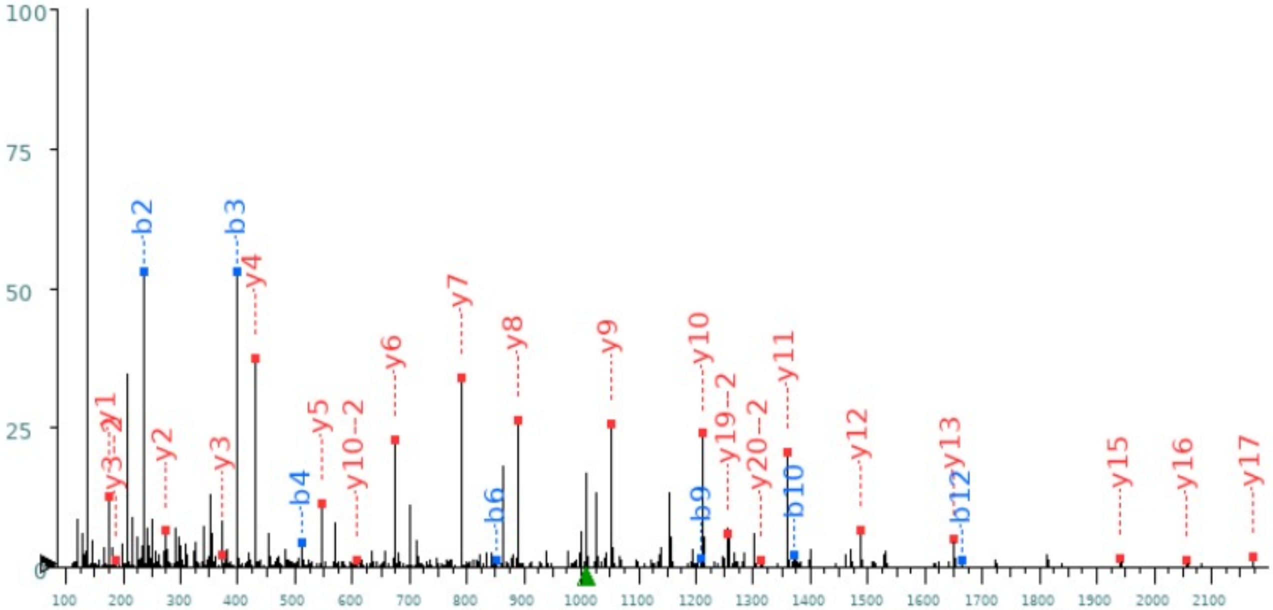

Sequence

AYYLPK#DDEYYQFCYVDEDGVVR

Predicted Fragmentation Pattern

+1

Seq	#	b: Δ Error	b	y	y: Δ Error	+1
A	1	---	72.044	---	---	23
Y	2	1.465	235.108	2950.288	---	22
Y	3	0.317	398.171	2787.224	---	21
L	4	3.195	511.255	2624.161	---	20
P	5	---	608.308	2511.077	---	19
K#	6	2.195	850.446	2414.024	---	18
D	7	---	965.473	2171.886	-3.939	17
D	8	---	1080.500	2056.859	-1.981	16
E	9	-6.567	1209.542	1941.832	-1.739	15
Y	10	-2.388	1372.606	1812.790	---	14
Y	11	---	1535.669	1649.726	3.482	13
Q	12	3.485	1663.727	1486.663	1.219	12
F	13	---	1810.796	1358.605	2.579	11
C	14	---	1970.827	1211.536	2.232	10
Y	15	---	2133.890	1051.505	0.955	9
V	16	---	2232.958	888.442	3.093	8
D	17	---	2347.985	789.374	1.695	7
E	18	---	2477.028	674.347	0.666	6
D	19	---	2592.055	545.304	1.030	5
G	20	---	2649.076	430.277	1.579	4
V	21	---	2748.145	373.256	-4.939	3
V	22	---	2847.213	274.187	1.711	2
R	23	---	---	175.119	2.033	1

+2

Seq	#	b: Δ Error	b	y	y: Δ Error	+1
A	1	---	36.526	---	---	23
Y	2	---	118.057	1475.647	---	22
Y	3	---	199.589	1394.116	---	21
L	4	---	256.131	1312.584	4.734	20
P	5	---	304.658	1256.042	9.547	19
K#	6	---	425.727	1207.516	---	18
D	7	---	483.240	1086.447	---	17
D	8	---	540.753	1028.933	---	16
E	9	---	605.275	971.420	---	15
Y	10	---	686.806	906.899	---	14
Y	11	---	768.338	825.367	---	13
Q	12	---	832.367	743.835	---	12
F	13	---	905.902	679.806	---	11
C	14	---	985.917	606.272	-1.857	10
Y	15	---	1067.449	526.256	---	9
V	16	---	1116.983	444.725	---	8
D	17	---	1174.496	395.190	---	7
E	18	---	1239.018	337.677	---	6
D	19	---	1296.531	273.156	---	5
G	20	---	1325.042	215.642	---	4
V	21	---	1374.576	187.132	70.867	3
V	22	---	1424.110	137.597	---	2
R	23	---	---	88.063	---	1

NDP52/CALCOCO2 K154

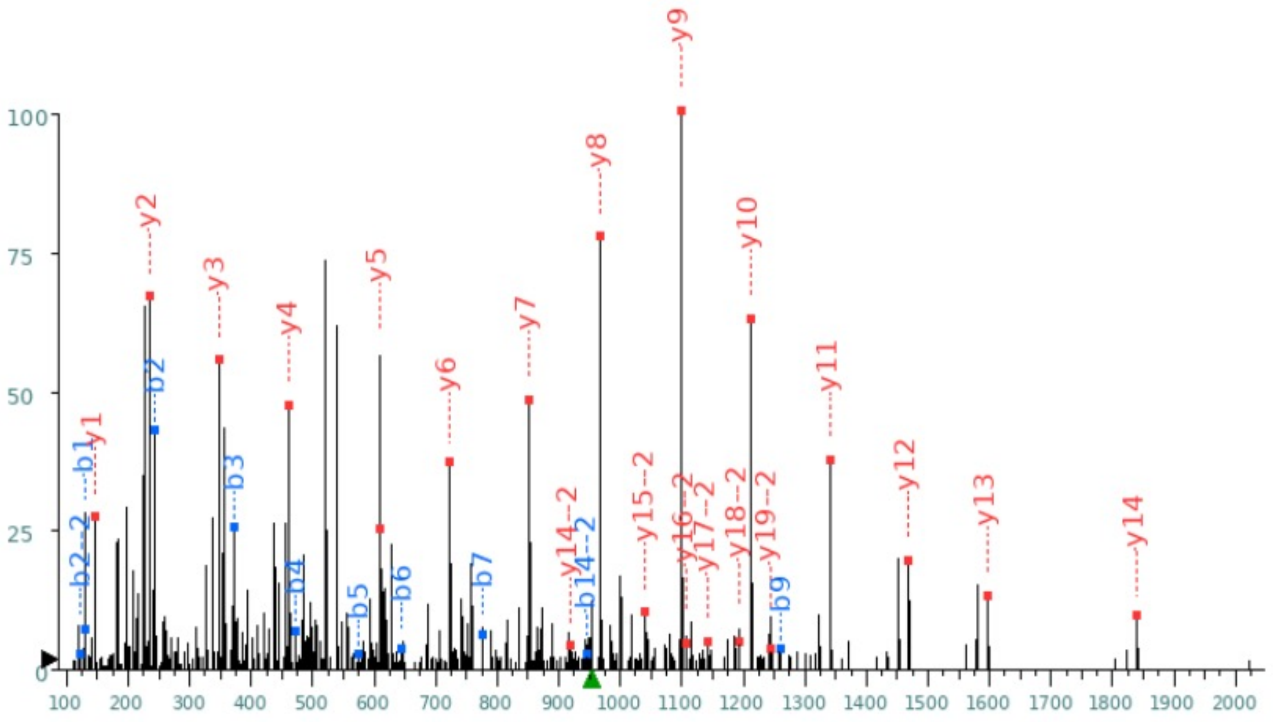

Sequence

QNETTAMK#K#QQELMDENFDLSK

Predicted Fragmentation Pattern

+1

Seq	#	b: Δ Error	b	y	y: Δ Error	+1
Q	1	4.046	**129.066**	---	---	22
N	2	2.569	**243.109**	2728.255	---	21
E	3	0.884	**372.151**	2614.212	---	20
T	4	1.125	**473.199**	2485.170	---	19
T	5	-3.767	**574.247**	2384.122	---	18
A	6	5.154	**645.284**	2283.074	---	17
M	7	18.019	**776.324**	2212.037	---	16
K#	8	---	1018.462	2080.997	---	15
K#	9	1.249	**1260.600**	**1838.859**	-7.511	14
Q	10	---	1388.659	**1596.721**	1.869	13
Q	11	---	1516.717	**1468.662**	-0.555	12
E	12	---	1645.760	**1340.604**	2.384	11
L	13	---	1758.844	**1211.561**	2.530	10
M	14	---	1889.884	**1098.477**	2.418	9
D	15	---	2004.911	**967.437**	0.998	8
E	16	---	2133.954	**852.410**	3.097	7
N	17	---	2247.997	**723.367**	3.974	6
F	18	---	2395.065	**609.324**	18.173	5
D	19	---	2510.092	**462.256**	2.023	4
L	20	---	2623.176	**347.229**	0.923	3
S	21	---	2710.208	**234.145**	1.188	2
K	22	---	---	**147.113**	1.685	1

+2

Seq	#	b: Δ Error	b	y	y: Δ Error	+1
Q	1	---	65.037	---	---	22
N	2	-28.498	**122.058**	1364.631	---	21
E	3	---	186.579	1307.610	---	20
T	4	---	237.103	**1243.089**	4.802	19
T	5	---	287.627	**1192.565**	12.098	18
A	6	---	323.146	**1142.041**	-0.910	17
M	7	---	388.666	**1106.522**	-0.386	16
K#	8	---	509.735	**1041.002**	-7.232	15
K#	9	---	630.804	**919.933**	1.609	14
Q	10	---	694.833	798.864	---	13
Q	11	---	758.862	734.835	---	12
E	12	---	823.384	670.806	---	11
L	13	---	879.926	606.284	---	10
M	14	-4.433	**945.446**	549.742	---	9
D	15	---	1002.959	484.222	---	8
E	16	---	1067.481	426.709	---	7
N	17	---	1124.502	362.187	---	6
F	18	---	1198.036	305.166	---	5
D	19	---	1255.550	231.632	---	4
L	20	---	1312.092	174.118	---	3
S	21	---	1355.608	117.576	---	2
K	22	---	---	74.060	---	1

NDP52/CALCOCO2 K242

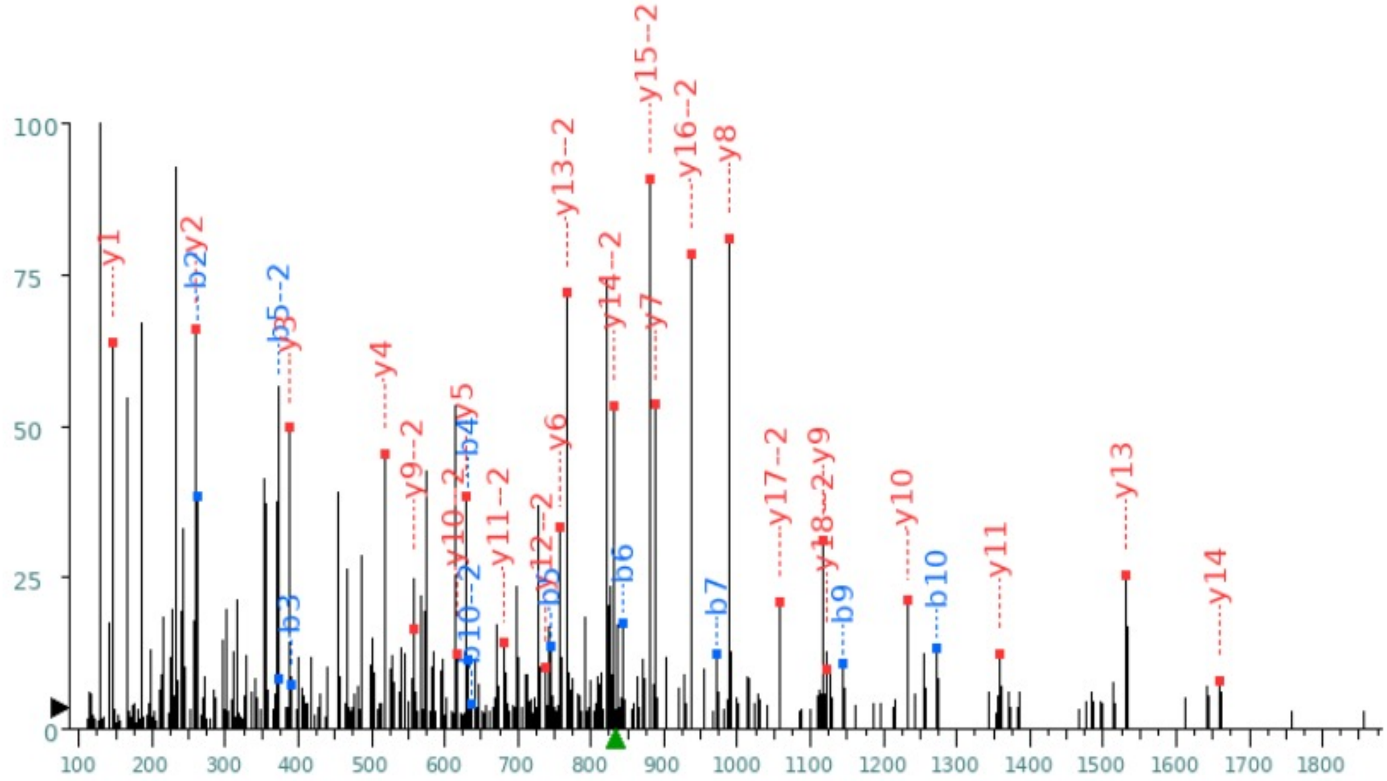

Sequence

EMEK#LVQGDQDKTEQLEQLK

Predicted Fragmentation Pattern

+1

Seq	#	b: Δ Error	b	y	y: Δ Error	+1
E	1	---	130.050	---	---	20
M	2	1.436	**261.090**	2374.192	---	19
E	3	2.785	**390.133**	2243.151	---	18
K#	4	-5.927	**632.271**	2114.109	---	17
L	5	1.662	**745.355**	1871.971	---	16
V	6	-0.838	**844.423**	1758.887	---	15
Q	7	-10.251	**972.482**	**1659.818**	0.478	14
G	8	---	1029.503	**1531.760**	-3.318	13
D	9	-11.115	**1144.530**	1474.738	---	12
Q	10	-9.408	**1272.589**	**1359.711**	3.763	11
D	11	---	1387.616	**1231.653**	1.664	10
K	12	---	1515.711	**1116.626**	3.444	9
T	13	---	1616.758	**988.531**	1.042	8
E	14	---	1745.801	**887.483**	1.860	7
Q	15	---	1873.860	**758.441**	5.866	6
L	16	---	1986.944	**630.382**	0.254	5
E	17	---	2115.986	**517.298**	3.177	4
Q	18	---	2244.045	**388.255**	-0.114	3
L	19	---	2357.129	**260.197**	0.236	2
K	20	---	---	**147.113**	0.751	1

+2

Seq	#	b: Δ Error	b	y	y: Δ Error	+1
E	1	---	65.529	---	---	20
M	2	---	131.049	1187.600	---	19
E	3	---	195.570	**1122.079**	12.961	18
K#	4	---	316.639	**1057.558**	4.455	17
L	5	24.049	**373.181**	**936.489**	0.248	16
V	6	---	422.715	**879.947**	0.725	15
Q	7	---	486.745	**830.413**	1.169	14
G	8	---	515.255	**766.384**	1.415	13
D	9	---	572.769	**737.873**	1.042	12
Q	10	-10.992	**636.798**	**680.359**	4.962	11
D	11	---	694.312	**616.330**	5.762	10
K	12	---	758.359	**558.817**	-1.649	9
T	13	---	808.883	494.769	---	8
E	14	---	873.404	444.245	---	7
Q	15	---	937.433	379.724	---	6
L	16	---	993.975	315.695	---	5
E	17	---	1058.497	259.153	---	4
Q	18	---	1122.526	194.631	---	3
L	19	---	1179.068	130.602	---	2
K	20	---	---	74.060	---	1

NDP52/CALCOCO2 K246

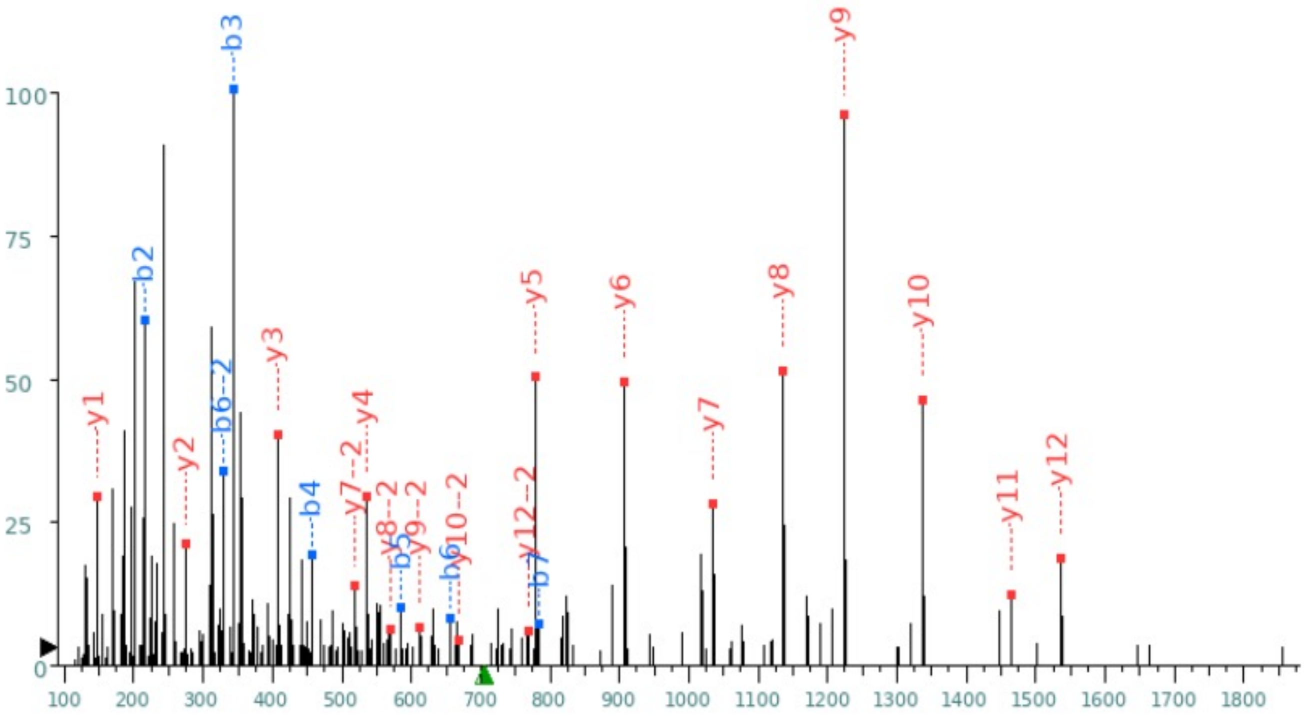

Sequence

VDQLQAQLSTQEK#EMEK

Predicted Fragmentation Pattern

+1

Seq	#	b: Δ Error	b	y	y: Δ Error	+1
V	1	---	100.076	---	---	17
D	2	3.678	215.103	2019.965	---	16
Q	3	2.175	343.161	1904.938	---	15
L	4	2.197	456.245	1776.880	---	14
Q	5	0.908	584.304	1663.796	---	13
A	6	2.758	655.341	1535.737	4.412	12
Q	7	5.367	783.400	1464.700	-1.288	11
L	8	---	896.484	1336.641	1.042	10
S	9	---	983.516	1223.557	3.299	9
T	10	---	1084.563	1136.525	1.873	8
Q	11	---	1212.622	1035.478	1.889	7
E	12	---	1341.664	907.419	2.137	6
K#	13	---	1583.802	778.376	5.303	5
E	14	---	1712.845	536.238	4.874	4
M	15	---	1843.885	407.196	-0.425	3
E	16	---	1972.928	276.155	2.755	2
K	17	---	---	147.113	3.344	1

+2

Seq	#	b: Δ Error	b	y	y: Δ Error	+1
V	1	---	50.541	---	---	17
D	2	---	108.055	1010.486	---	16
Q	3	---	172.084	952.973	---	15
L	4	---	228.626	888.943	---	14
Q	5	---	292.656	832.401	---	13
A	6	-37.346	328.174	768.372	4.520	12
Q	7	---	392.203	732.854	---	11
L	8	---	448.745	668.824	2.264	10
S	9	---	492.261	612.282	-1.749	9
T	10	---	542.785	568.766	8.889	8
Q	11	---	606.815	518.242	-17.028	7
E	12	---	671.336	454.213	---	6
K#	13	---	792.405	389.692	---	5
E	14	---	856.926	268.623	---	4
M	15	---	922.446	204.102	---	3
E	16	---	986.968	138.581	---	2
K	17	---	---	74.060	---	1

NDP52/CALCOCO2 K263

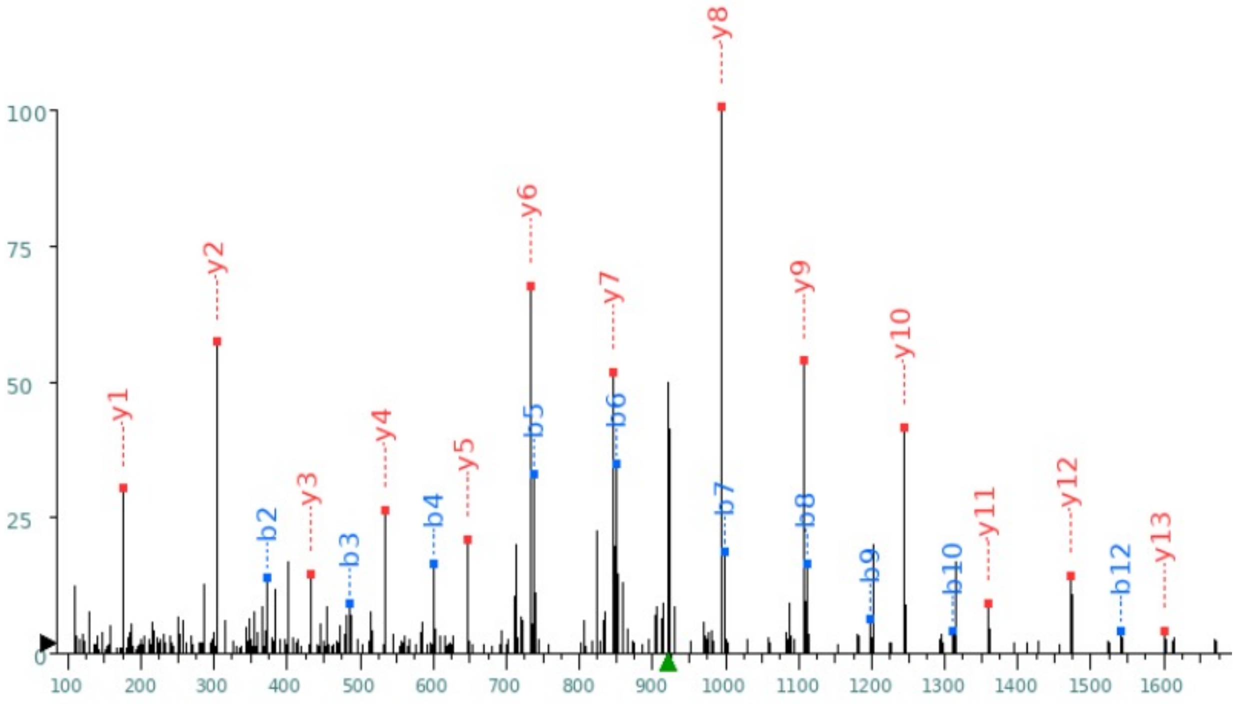

Sequence

K#ENDHLFLSLTEQR

Predicted Fragmentation Pattern

Seq	#	b: Δ Error	b	y	y: Δ Error	+1
K#	1	---	243.145	---	---	14
E	2	1.762	372.188	1601.792	-1.259	13
N	3	2.627	486.231	1472.749	5.338	12
D	4	3.146	601.258	1358.706	6.295	11
H	5	2.296	738.317	1243.679	3.414	10
L	6	3.045	851.401	1106.620	3.573	9
F	7	3.887	998.469	993.536	3.322	8
L	8	2.980	1111.553	846.468	3.099	7
S	9	-10.922	1198.585	733.384	3.602	6
L	10	-0.641	1311.669	646.352	4.536	5
T	11	---	1412.717	533.268	3.930	4
E	12	3.927	1541.759	432.220	2.472	3
Q	13	---	1669.818	303.178	1.882	2
R	14	---	---	175.119	3.601	1

NDP52/CALCOCO2 K281

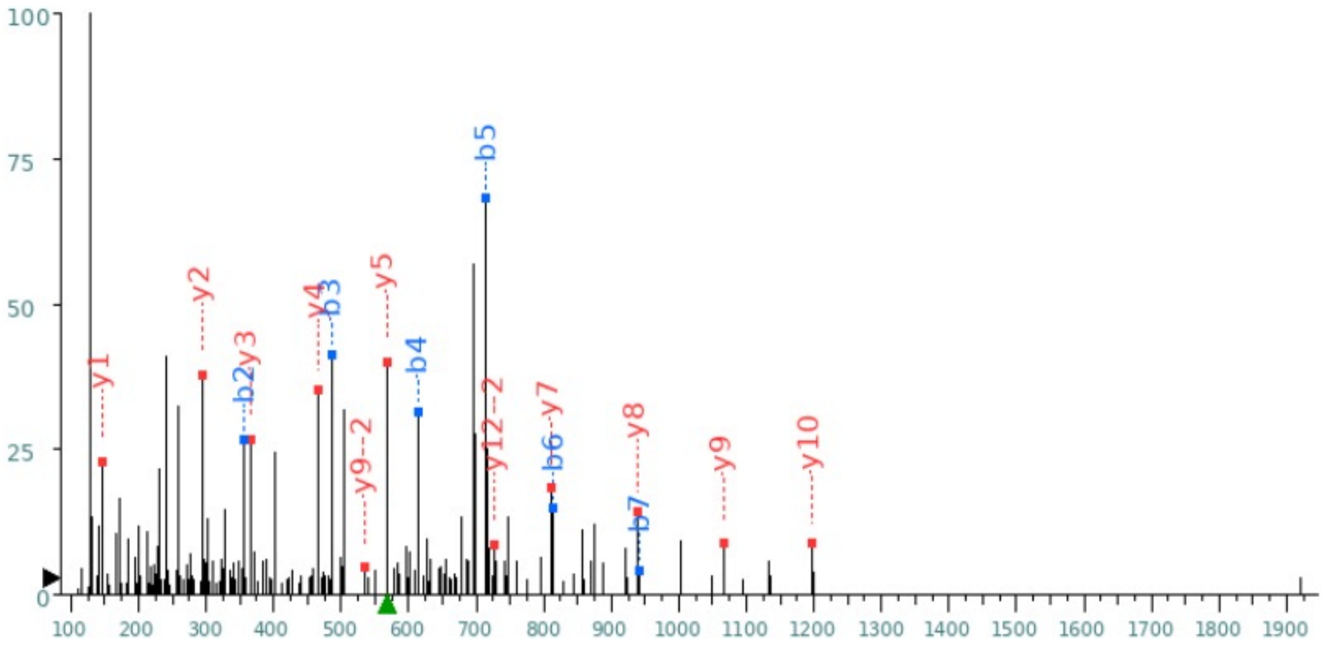

Sequence

K#LEQTVEQMKQNETTAM*K

Predicted Fragmentation Pattern

+1

Seq	#	b: Δ Error	b	y	y: Δ Error	+1
K#	1	---	243.145	---	---	18
L	2	5.104	**356.229**	2024.963	---	17
E	3	4.898	**485.272**	1911.879	---	16
Q	4	8.430	**613.330**	1782.836	---	15
T	5	2.866	**714.378**	1654.777	---	14
V	6	13.254	**813.446**	1553.730	---	13
E	7	-7.525	**942.489**	1454.661	---	12
Q	8	---	1070.548	1325.619	---	11
M	9	---	1201.588	**1197.560**	3.025	10
K	10	---	1329.683	**1066.520**	1.983	9
Q	11	---	1457.742	**938.425**	0.229	8
N	12	---	1571.785	**810.366**	-0.357	7
E	13	---	1700.827	696.323	---	6
T	14	---	1801.875	**567.281**	-2.751	5
T	15	---	1902.923	**466.233**	4.660	4
A	16	---	1973.960	**365.185**	1.547	3
M*	17	---	2120.995	**294.148**	1.832	2
K	18	---	---	**147.113**	1.996	1

+2

Seq	#	b: Δ Error	b	y	y: Δ Error	+1
K#	1	---	122.076	---	---	18
L	2	---	178.618	1012.985	---	17
E	3	---	243.140	956.443	---	16
Q	4	---	307.169	891.922	---	15
T	5	---	357.693	827.892	---	14
V	6	---	407.227	777.369	---	13
E	7	---	471.748	**727.834**	3.881	12
Q	8	---	535.777	663.313	---	11
M	9	---	601.298	599.284	---	10
K	10	---	665.345	**533.764**	-0.032	9
Q	11	---	729.374	469.716	---	8
N	12	---	786.396	405.687	---	7
E	13	---	850.917	348.665	---	6
T	14	---	901.441	284.144	---	5
T	15	---	951.965	233.620	---	4
A	16	---	987.483	183.096	---	3
M*	17	---	1061.001	147.578	---	2
K	18	---	---	74.060	---	1

+3

Seq	#	b: Δ Error	b	y	y: Δ Error	+1
K#	1	---	81.720	---	---	18
L	2	---	119.415	675.659	---	17
E	3	---	162.429	637.964	---	16
Q	4	---	205.115	594.950	---	15
T	5	---	238.798	552.264	---	14
V	6	---	271.820	518.581	---	13
E	7	---	314.835	485.559	---	12
Q	8	---	357.521	442.544	---	11
M	9	---	401.201	399.858	---	10
K	10	---	443.899	356.178	---	9
Q	11	---	486.585	313.480	---	8
N	12	---	524.600	270.794	---	7
E	13	---	567.614	232.779	---	6
T	14	---	601.296	189.765	---	5
T	15	---	634.979	156.083	---	4
A	16	---	658.658	122.400	---	3
M*	17	---	707.670	98.721	---	2
K	18	---	---	49.709	---	1

NDP52/CALCOCO2 K298,K299

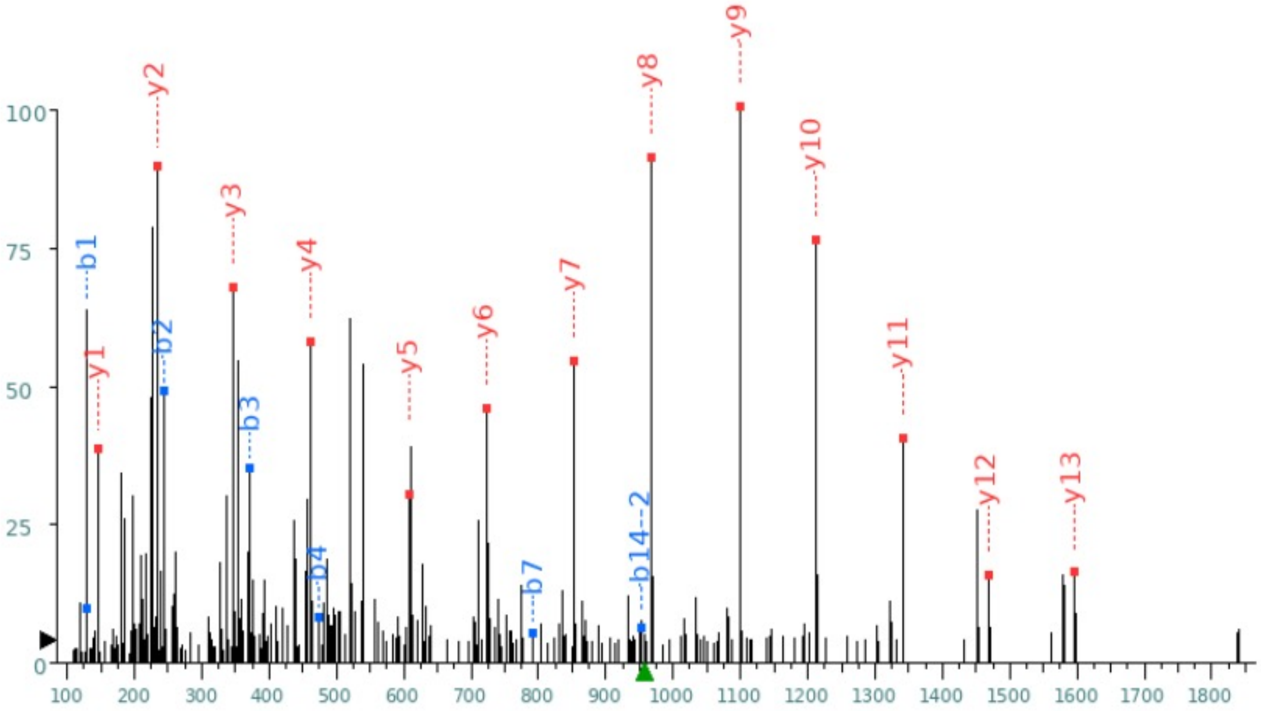

Sequence

QNETTAM*K#K#QQELMDENFDLSK

Predicted Fragmentation Pattern

+1

Seq	#	b: Δ Error	b	y	y: Δ Error	+1
Q	1	4.283	**129.066**	---	---	22
N	2	5.268	**243.109**	2744.250	---	21
E	3	2.278	**372.151**	2630.207	---	20
T	4	6.349	**473.199**	2501.165	---	19
T	5	---	574.247	2400.117	---	18
A	6	---	645.284	2299.069	---	17
M*	7	13.520	**792.319**	2228.032	---	16
K#	8	---	1034.457	2080.997	---	15
K#	9	---	1276.595	1838.859	---	14
Q	10	---	1404.654	**1596.721**	1.411	13
Q	11	---	1532.712	**1468.662**	1.606	12
E	12	---	1661.755	**1340.604**	4.661	11
L	13	---	1774.839	**1211.561**	2.328	10
M	14	---	1905.879	**1098.477**	4.085	9
D	15	---	2020.906	**967.437**	3.332	8
E	16	---	2149.949	**852.410**	1.880	7
N	17	---	2263.992	**723.367**	3.046	6
F	18	---	2411.060	**609.324**	14.967	5
D	19	---	2526.087	**462.256**	5.390	4
L	20	---	2639.171	**347.229**	3.648	3
S	21	---	2726.203	**234.145**	2.882	2
K	22	---	---	**147.113**	2.929	1

+2

Seq	#	b: Δ Error	b	y	y: Δ Error	+1
Q	1	---	65.037	---	---	22
N	2	---	122.058	1372.629	---	21
E	3	---	186.579	1315.607	---	20
T	4	---	237.103	1251.086	---	19
T	5	---	287.627	1200.562	---	18
A	6	---	323.146	1150.038	---	17
M*	7	---	396.663	1114.520	---	16
K#	8	---	517.732	1041.002	---	15
K#	9	---	638.801	919.933	---	14
Q	10	---	702.830	798.864	---	13
Q	11	---	766.860	734.835	---	12
E	12	---	831.381	670.806	---	11
L	13	---	887.923	606.284	---	10
M	14	-0.001	**953.443**	549.742	---	9
D	15	---	1010.957	484.222	---	8
E	16	---	1075.478	426.709	---	7
N	17	---	1132.500	362.187	---	6
F	18	---	1206.034	305.166	---	5
D	19	---	1263.547	231.632	---	4
L	20	---	1320.089	174.118	---	3
S	21	---	1363.605	117.576	---	2
K	22	---	---	74.060	---	1

NDP52/CALCOCO2 K395

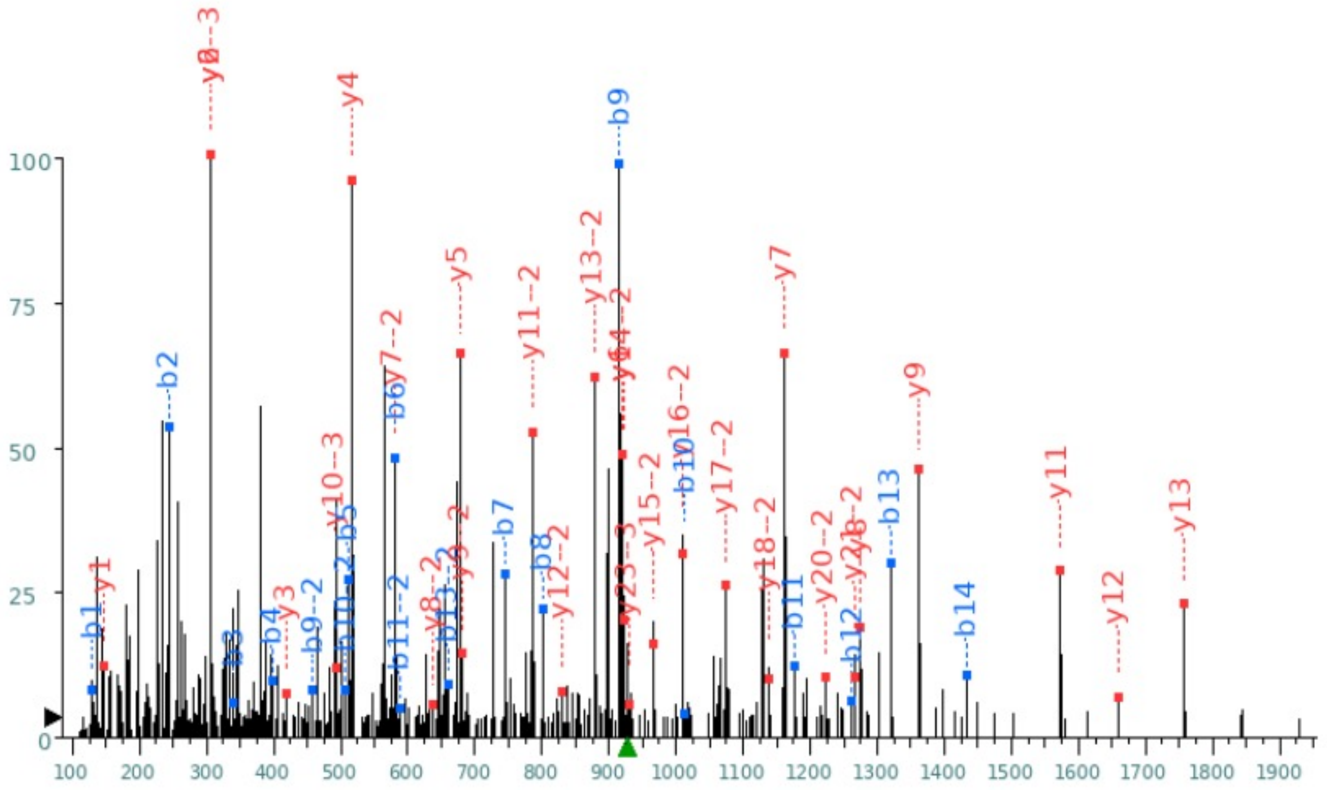

Sequence

QNPGLAYGNPYSGIQESSSPSPLSIK#K#CPICK

Predicted Fragmentation Pattern

+1

Seq	#	b: Δ Error	b	y	y: Δ Error	+1
Q	1	5.583	129.066	---	---	32
N	2	2.820	243.109	3577.741	---	31
P	3	2.019	340.162	3463.698	---	30
G	4	2.088	397.183	3366.646	---	29
L	5	2.008	510.267	3309.624	---	28
A	6	1.282	581.304	3196.540	---	27
Y	7	5.382	744.368	3125.503	---	26
G	8	2.131	801.389	2962.440	---	25
N	9	1.644	915.432	2905.418	---	24
P	10	1.819	1012.485	2791.375	---	23
Y	11	4.444	1175.548	2694.323	---	22
S	12	2.458	1262.580	2531.259	---	21
G	13	2.460	1319.601	2444.227	---	20
I	14	8.941	1432.686	2387.206	---	19
Q	15	---	1560.744	2274.122	---	18
E	16	---	1689.787	2146.063	---	17
S	17	---	1776.819	2017.021	---	16
S	18	---	1863.851	1929.988	---	15
S	19	---	1950.883	1842.956	---	14
P	20	---	2047.936	1755.924	0.983	13
S	21	---	2134.968	1658.872	-5.344	12
P	22	---	2232.020	1571.840	4.718	11
L	23	---	2345.104	1474.787	---	10
S	24	---	2432.136	1361.703	4.271	9
I	25	---	2545.221	1274.671	6.418	8
K#	26	---	2787.358	1161.587	3.434	7
K#	27	---	3029.496	919.449	2.227	6
C	28	---	3189.527	677.311	0.698	5
P	29	---	3286.580	517.280	2.936	4
I	30	---	3399.664	420.228	5.137	3
C	31	---	3559.694	307.143	1.822	2
K	32	---	---	147.113	3.552	1

+2

Seq	#	b: Δ Error	b	y	y: Δ Error	+1
Q	1	---	65.037	---	---	32
N	2	---	122.058	1789.374	---	31
P	3	---	170.584	1732.353	---	30
G	4	---	199.095	1683.826	---	29
L	5	---	255.637	1655.316	---	28
A	6	---	291.156	1598.774	---	27
Y	7	---	372.687	1563.255	---	26
G	8	---	401.198	1481.723	---	25
N	9	1.162	458.220	1453.213	---	24
P	10	3.250	506.746	1396.191	---	23
Y	11	4.481	588.278	1347.665	---	22
S	12	---	631.794	1266.133	3.893	21
G	13	-17.287	660.304	1222.617	10.540	20
I	14	---	716.846	1194.107	---	19
Q	15	---	780.876	1137.564	-3.133	18
E	16	---	845.397	1073.535	4.405	17
S	17	---	888.913	1009.014	1.415	16
S	18	---	932.429	965.498	7.256	15
S	19	---	975.945	921.982	5.174	14
P	20	---	1024.471	878.466	5.664	13
S	21	---	1067.987	829.939	1.675	12
P	22	---	1116.514	786.423	3.970	11
L	23	---	1173.056	737.897	---	10
S	24	---	1216.572	681.355	0.631	9
I	25	---	1273.114	637.839	0.710	8
K#	26	---	1394.183	581.297	13.657	7
K#	27	---	1515.252	460.228	---	6
C	28	---	1595.267	339.159	---	5
P	29	---	1643.793	259.144	---	4
I	30	---	1700.336	210.617	---	3
C	31	---	1780.351	154.075	---	2
K	32	---	---	74.060	---	1

+3

Seq	#	b: Δ Error	b	y	y: Δ Error	+1
Q	1	---	43.693	---	---	32
N	2	---	81.708	1193.252	---	31
P	3	---	114.059	1155.238	---	30
G	4	---	133.066	1122.887	---	29
L	5	---	170.761	1103.880	---	28
A	6	---	194.440	1066.185	---	27
Y	7	---	248.794	1042.506	---	26
G	8	---	267.801	988.151	---	25
N	9	---	305.815	969.144	---	24
P	10	---	338.166	931.130	9.029	23
Y	11	---	392.521	898.779	---	22
S	12	---	421.532	844.425	---	21
G	13	---	440.539	815.414	---	20
I	14	---	478.233	796.407	---	19
Q	15	---	520.920	758.712	---	18
E	16	---	563.934	716.026	---	17
S	17	---	592.944	673.012	---	16
S	18	---	621.955	644.001	---	15
S	19	---	650.966	614.990	---	14
P	20	---	683.317	585.980	---	13
S	21	---	712.327	553.629	---	12
P	22	---	744.678	524.618	---	11
L	23	---	782.373	492.267	-15.785	10
S	24	---	811.384	454.572	---	9
I	25	---	849.078	425.562	---	8
K#	26	---	929.791	387.867	---	7
K#	27	---	1010.504	307.154	-34.006	6
C	28	---	1063.847	226.442	---	5
P	29	---	1096.198	173.098	---	4
I	30	---	1133.893	140.747	---	3
C	31	---	1187.236	103.053	---	2
K	32	---	---	49.709	---	1

NDP52/CALCOCO2 K401

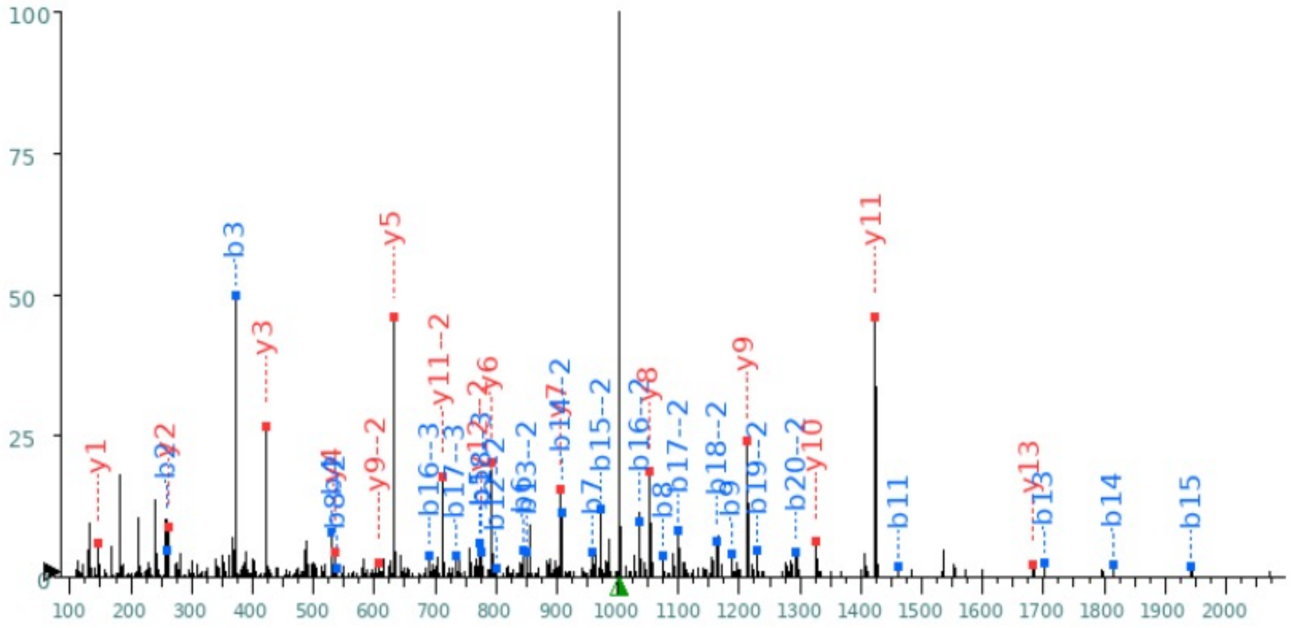

Sequence

CPICK#ADDICDHTLEQQQMQPLCFNCPICDK

Predicted Fragmentation Pattern

+1

Seq	#	b: Δ Error	b	y	y: Δ Error	+1
C	1	---	161.038	---	---	31
P	2	-13.914	**258.091**	3848.663	---	30
I	3	1.457	**371.175**	3751.610	---	29
C	4	1.462	**531.205**	3638.526	---	28
K#	5	-3.747	**773.343**	3478.495	---	27
A	6	-0.473	**844.380**	3236.357	---	26
D	7	2.864	**959.407**	3165.320	---	25
D	8	-1.330	**1074.434**	3050.293	---	24
I	9	-0.551	**1187.518**	2935.266	---	23
C	10	---	1347.549	2822.182	---	22
D	11	2.074	**1462.576**	2662.152	---	21
H	12	---	1599.635	2547.125	---	20
T	13	-1.029	**1700.683**	2410.066	---	19
L	14	0.674	**1813.767**	2309.018	---	18
E	15	3.273	**1942.809**	2195.934	---	17
Q	16	---	2070.868	2066.891	---	16
Q	17	---	2198.926	1938.833	---	15
Q	18	---	2326.985	1810.774	---	14
M	19	---	2458.025	**1682.716**	-2.353	13
Q	20	---	2586.084	1551.675	---	12
P	21	---	2683.137	**1423.617**	1.379	11
L	22	---	2796.221	**1326.564**	2.238	10
C	23	---	2956.251	**1213.480**	0.702	9
F	24	---	3103.320	**1053.449**	0.932	8
N	25	---	3217.363	**906.381**	-0.674	7
C	26	---	3377.393	**792.338**	-7.140	6
P	27	---	3474.446	**632.307**	-1.791	5
I	28	---	3587.530	**535.254**	-1.490	4
C	29	---	3747.561	**422.170**	2.998	3
D	30	---	3862.588	**262.140**	1.485	2
K	31	---	---	**147.113**	2.203	1

+2

Seq	#	b: Δ Error	b	y	y: Δ Error	+1
C	1	---	81.023	---	---	31
P	2	---	129.549	1924.835	---	30
I	3	---	186.091	1876.309	---	29
C	4	---	266.106	1819.767	---	28
K#	5	---	387.175	1739.751	---	27
A	6	---	422.694	1618.682	---	26
D	7	---	480.207	1583.164	---	25
D	8	-0.036	**537.721**	1525.650	---	24
I	9	---	594.263	1468.137	---	23
C	10	---	674.278	1411.595	---	22
D	11	---	731.792	1331.579	---	21
H	12	-5.823	**800.321**	1274.066	---	20
T	13	7.178	**850.845**	1205.537	---	19
L	14	-3.067	**907.387**	1155.013	---	18
E	15	2.228	**971.908**	1098.471	---	17
Q	16	2.334	**1035.938**	1033.949	---	16
Q	17	0.319	**1099.967**	969.920	---	15
Q	18	-0.426	**1163.996**	905.891	---	14
M	19	-0.088	**1229.516**	841.862	---	13
Q	20	1.244	**1293.546**	**776.341**	-10.251	12
P	21	---	1342.072	**712.312**	0.898	11
L	22	---	1398.614	663.786	---	10
C	23	---	1478.629	**607.244**	6.671	9
F	24	---	1552.164	527.228	---	8
N	25	---	1609.185	453.694	---	7
C	26	---	1689.200	396.673	---	6
P	27	---	1737.727	316.657	---	5
I	28	---	1794.269	268.131	---	4
C	29	---	1874.284	211.589	---	3
D	30	---	1931.798	131.574	---	2
K	31	---	---	74.060	---	1

+3

Seq	#	b: Δ Error	b	y	y: Δ Error	+1
C	1	---	54.351	---	---	31
P	2	---	86.702	1283.559	---	30
I	3	---	124.396	1251.208	---	29
C	4	---	177.740	1213.513	---	28
K#	5	---	258.453	1160.170	---	27
A	6	---	282.132	1079.457	---	26
D	7	---	320.474	1055.778	---	25
D	8	---	358.816	1017.436	---	24
I	9	---	396.511	979.094	---	23
C	10	---	449.855	941.399	---	22
D	11	---	488.197	888.055	---	21
H	12	---	533.883	849.713	---	20
T	13	---	567.566	804.027	---	19
L	14	---	605.260	770.344	---	18
E	15	---	648.275	732.650	---	17
Q	16	0.060	**690.961**	689.635	---	16
Q	17	0.507	**733.647**	646.949	---	15
Q	18	0.198	**776.333**	604.263	---	14
M	19	---	820.013	561.577	---	13
Q	20	---	862.700	517.897	---	12
P	21	---	895.050	475.210	---	11
L	22	---	932.745	442.859	---	10
C	23	---	986.089	405.165	---	9
F	24	---	1035.111	351.821	---	8
N	25	---	1073.126	302.798	---	7
C	26	---	1126.469	264.784	---	6
P	27	---	1158.820	211.441	---	5
I	28	---	1196.515	179.090	---	4
C	29	---	1249.858	141.395	---	3
D	30	---	1288.201	88.051	---	2
K	31	---	---	49.709	---	1

NDP52/CALCOCO2 K434

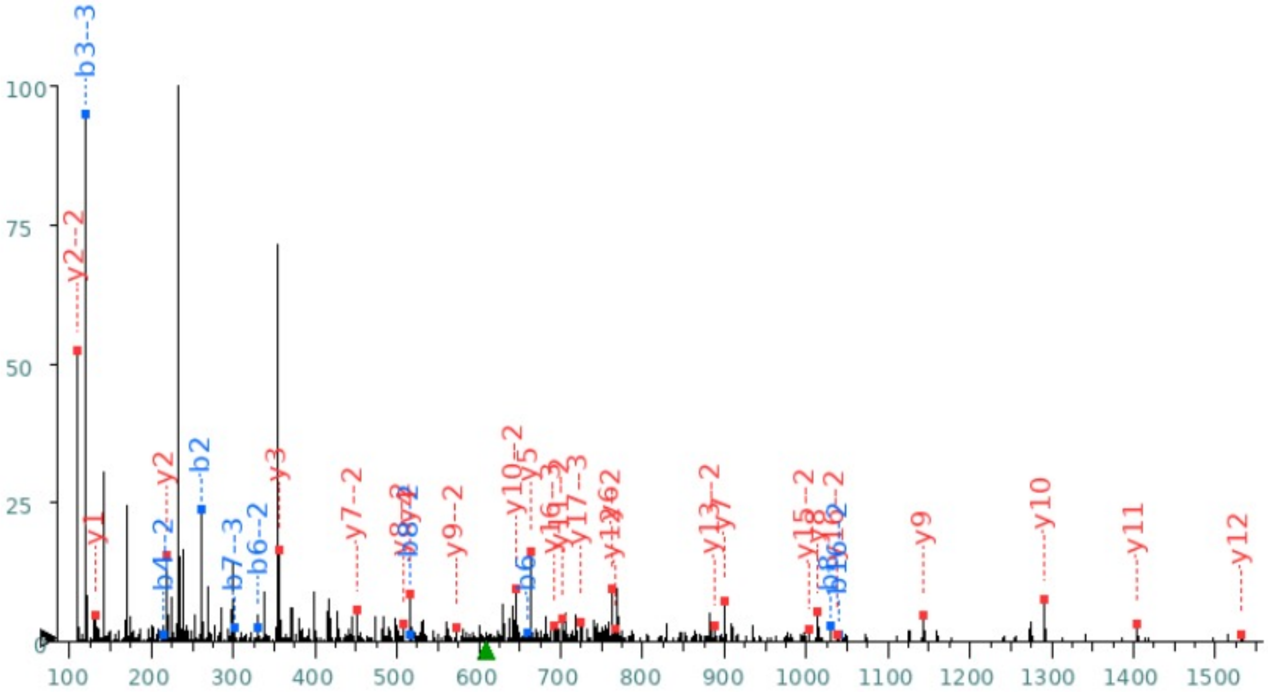

Sequence

IFPATEK#QIFEDHVFCHSL

Predicted Fragmentation Pattern

+1

Seq	#	b: Δ Error	b	y	y: Δ Error	+1
I	1	---	114.091	---	---	19
F	2	-0.913	261.160	2319.086	---	18
P	3	---	358.213	2172.018	---	17
A	4	---	429.250	2074.965	---	16
T	5	---	530.297	2003.928	---	15
E	6	18.979	659.340	1902.880	---	14
K#	7	---	901.478	1773.838	---	13
Q	8	-6.397	1029.536	1531.700	-2.229	12
I	9	---	1142.620	1403.641	-1.748	11
F	10	---	1289.689	1290.557	0.998	10
E	11	---	1418.731	1143.489	-2.242	9
D	12	---	1533.758	1014.446	1.254	8
H	13	---	1670.817	899.419	0.222	7
V	14	---	1769.886	762.360	0.599	6
F	15	---	1916.954	663.292	-0.425	5
C	16	---	2076.985	516.223	1.688	4
H	17	---	2214.044	356.193	0.242	3
S	18	---	2301.076	219.134	-0.382	2
L	19	---	---	132.102	0.753	1

+2

Seq	#	b: Δ Error	b	y	y: Δ Error	+1
I	1	---	57.549	---	---	19
F	2	---	131.084	1160.047	---	18
P	3	---	179.610	1086.513	---	17
A	4	46.999	215.128	1037.986	-0.689	16
T	5	---	265.652	1002.468	9.152	15
E	6	25.770	330.174	951.944	---	14
K#	7	---	451.243	887.422	-3.726	13
Q	8	-25.415	515.272	766.354	2.105	12
I	9	---	571.814	702.324	-3.538	11
F	10	---	645.348	645.782	-0.950	10
E	11	---	709.869	572.248	-3.051	9
D	12	---	767.383	507.727	-4.891	8
H	13	---	835.912	450.213	-1.894	7
V	14	---	885.446	381.684	---	6
F	15	---	958.981	332.150	---	5
C	16	-11.917	1038.996	258.615	---	4
H	17	---	1107.525	178.600	---	3
S	18	---	1151.041	110.071	8.849	2
L	19	---	---	66.555	---	1

+3

Seq	#	b: Δ Error	b	y	y: Δ Error	+1
I	1	---	38.702	---	---	19
F	2	---	87.725	773.700	---	18
P	3	44.104	120.076	724.677	0.867	17
A	4	---	143.755	692.327	13.822	16
T	5	---	177.437	668.648	---	15
E	6	---	220.451	634.965	---	14
K#	7	22.523	301.164	591.951	---	13
Q	8	---	343.850	511.238	---	12
I	9	---	381.545	468.552	---	11
F	10	---	430.568	430.857	---	10
E	11	---	473.582	381.834	---	9
D	12	---	511.924	338.820	---	8
H	13	---	557.611	300.478	---	7
V	14	---	590.633	254.792	---	6
F	15	---	639.656	221.769	---	5
C	16	---	693.000	172.746	---	4
H	17	---	738.686	119.402	---	3
S	18	---	767.697	73.716	---	2
L	19	---	---	44.705	---	1

NDP52- Ub K63

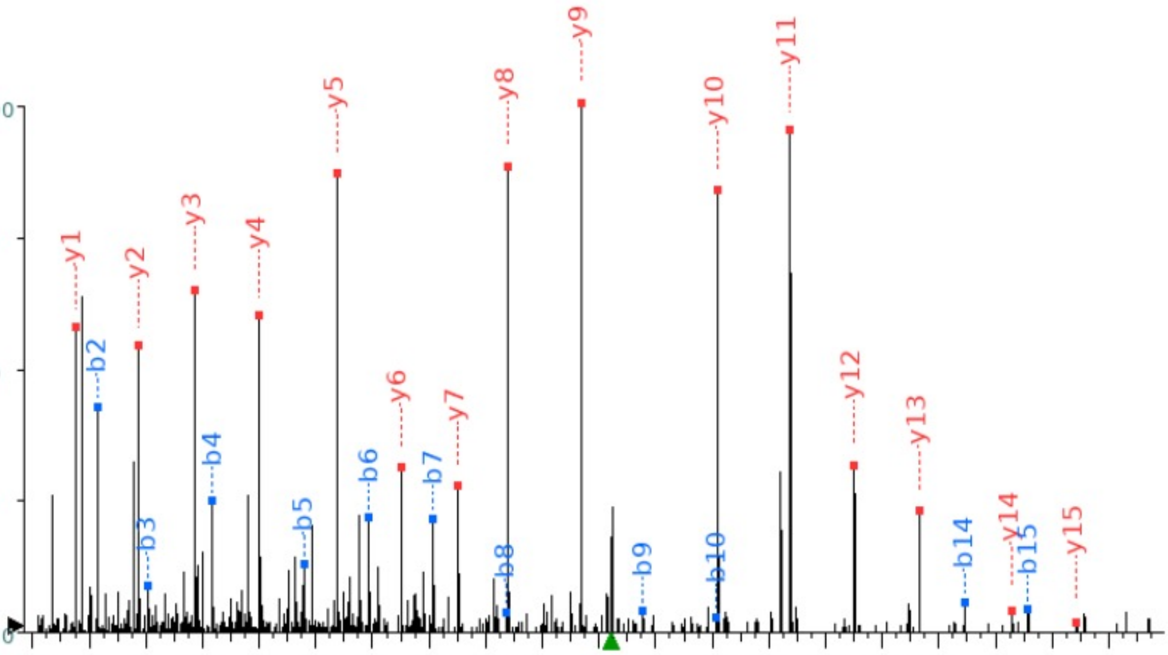

Sequence

TLSDYNIQK#ESTLHLVLR

Predicted Fragmentation Pattern

Seq	#	b: Δ Error	b	y	y: Δ Error	+1
T	1	---	102.055	---	---	18
L	2	0.871	215.139	2143.151	---	17
S	3	-0.188	302.171	2030.067	---	16
D	4	-0.054	417.198	1943.035	0.023	15
Y	5	-0.572	580.261	1828.008	3.278	14
N	6	0.548	694.304	1664.944	1.823	13
I	7	0.448	807.388	1550.901	1.459	12
Q	8	1.514	935.447	1437.817	1.629	11
K#	9	1.088	1177.585	1309.759	0.937	10
E	10	7.995	1306.627	1067.621	1.218	9
S	11	---	1393.659	938.578	2.351	8
T	12	---	1494.707	851.546	1.426	7
L	13	---	1607.791	750.498	0.412	6
H	14	-0.395	1744.850	637.414	0.131	5
L	15	-1.596	1857.934	500.355	0.437	4
V	16	---	1957.003	387.271	0.297	3
L	17	---	2070.087	288.203	0.588	2
R	18	---	---	175.119	1.074	1

NDP52-Ub K11

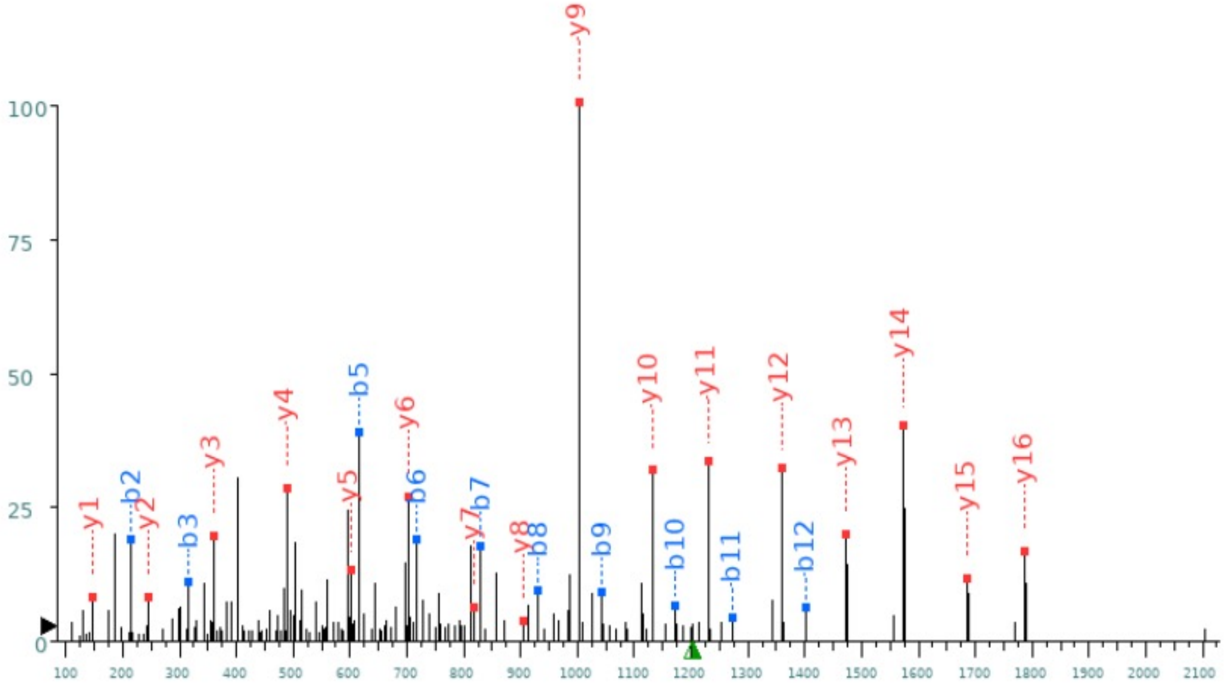

Sequence

TLTGK#TITLEVEPSDTIENVK

Predicted Fragmentation Pattern

Seq	#	b: Δ Error	b	y	y: Δ Error	+1
T	1	---	102.055	---	---	21
L	2	1.581	215.139	2301.218	---	20
T	3	0.416	316.187	2188.134	---	19
G	4	---	373.208	2087.087	---	18
K#	5	3.104	615.346	2030.065	---	17
T	6	-0.585	716.394	1787.927	3.314	16
I	7	0.135	829.478	1686.880	2.759	15
T	8	10.079	930.525	1573.796	1.069	14
L	9	14.116	1043.610	1472.748	4.506	13
E	10	4.763	1172.652	1359.664	1.438	12
V	11	-5.921	1271.721	1230.621	2.870	11
E	12	9.707	1400.763	1131.553	0.796	10
P	13	---	1497.816	1002.510	1.072	9
S	14	---	1584.848	905.457	2.703	8
D	15	---	1699.875	818.425	1.778	7
T	16	---	1800.923	703.398	-0.062	6
I	17	---	1914.007	602.351	4.706	5
E	18	---	2043.049	489.267	1.154	4
N	19	---	2157.092	360.224	1.456	3
V	20	---	2256.161	246.181	-1.012	2
K	21	---	---	147.113	1.788	1

OPTN K78

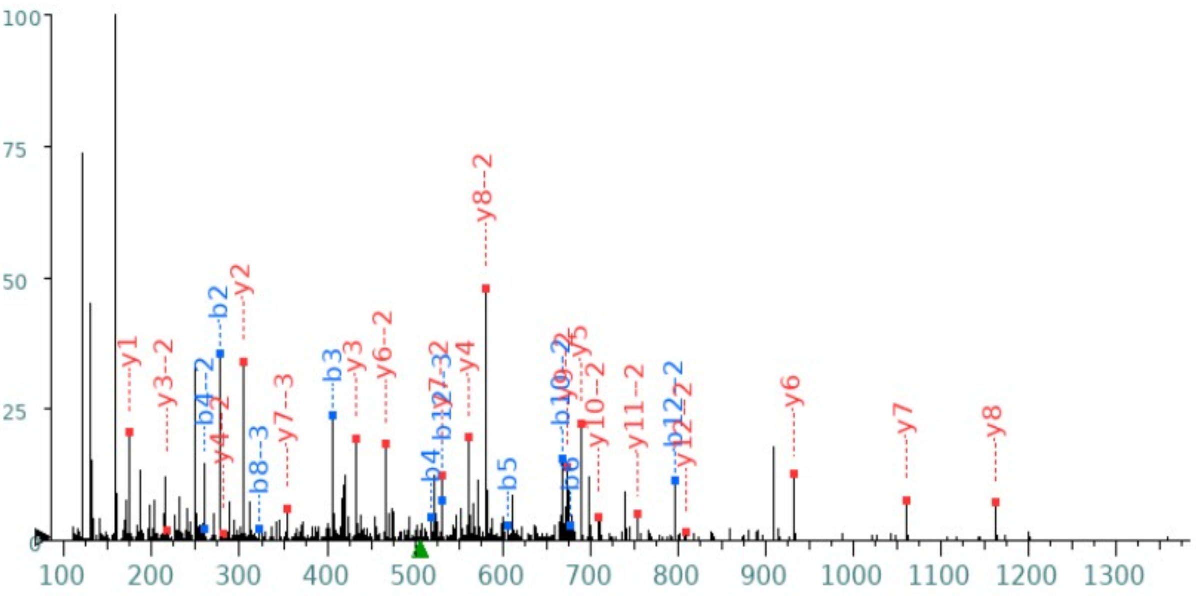

Sequence

FEELSAWTEK#QKEER

Predicted Fragmentation Pattern

+1

Seq	#	b: Δ Error	b	y	y: Δ Error	+1
F	1	---	148.076	---	---	15
E	2	0.011	277.118	1876.904	---	14
E	3	-1.472	406.161	1747.861	---	13
L	4	4.690	519.245	1618.818	---	12
S	5	-2.703	606.277	1505.734	---	11
A	6	5.774	677.314	1418.702	---	10
W	7	---	863.393	1347.665	---	9
T	8	---	964.441	1161.586	-2.124	8
E	9	---	1093.484	1060.538	1.309	7
K#	10	---	1335.622	931.496	1.152	6
Q	11	---	1463.680	689.358	-0.728	5
K	12	---	1591.775	561.299	2.014	4
E	13	---	1720.818	433.204	0.055	3
E	14	---	1849.860	304.162	0.449	2
R	15	---	---	175.119	0.203	1

+2

Seq	#	b: Δ Error	b	y	y: Δ Error	+1
F	1	---	74.541	---	---	15
E	2	---	139.063	938.955	---	14
E	3	---	203.584	874.434	---	13
L	4	46.889	260.126	809.913	-11.124	12
S	5	---	303.642	753.371	-1.617	11
A	6	---	339.161	709.855	-0.910	10
W	7	---	432.200	674.336	-0.502	9
T	8	---	482.724	581.297	-0.506	8
E	9	---	547.245	530.773	-0.832	7
K#	10	-18.302	668.314	466.251	0.286	6
Q	11	---	732.344	345.182	---	5
K	12	8.031	796.391	281.153	-3.657	4
E	13	---	860.912	217.106	-41.373	3
E	14	---	925.434	152.584	---	2
R	15	---	---	88.063	---	1

+3

Seq	#	b: Δ Error	b	y	y: Δ Error	+1
F	1	---	50.030	---	---	15
E	2	---	93.044	626.306	---	14
E	3	---	136.058	583.292	---	13
L	4	---	173.753	540.278	---	12
S	5	---	202.764	502.583	---	11
A	6	---	226.443	473.572	---	10
W	7	---	288.469	449.893	---	9
T	8	-5.366	322.152	387.867	---	8
E	9	---	365.166	354.184	-35.769	7
K#	10	---	445.879	311.170	---	6
Q	11	---	488.565	230.457	---	5
K	12	18.894	531.263	187.771	---	4
E	13	---	574.277	145.073	---	3
E	14	---	617.292	102.059	---	2
R	15	---	---	59.045	---	1

OPTN K106

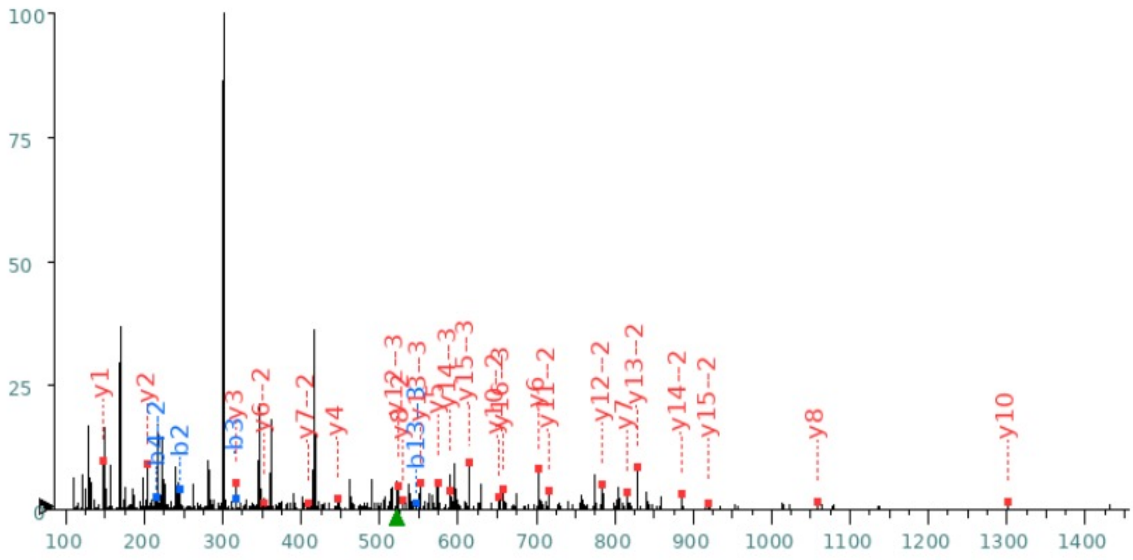

Sequence

LMALSHENEK‡LKEELGK

Predicted Fragmentation Pattern

+1

Seq	#	b: Δ Error	b	y	y: Δ Error	+1
L	1	---	114.091	---	---	17
M	2	3.657	245.132	1970.001	---	16
A	3	-1.619	316.169	1838.961	---	15
L	4	---	429.253	1767.924	---	14
S	5	---	516.285	1654.839	---	13
H	6	---	653.344	1567.807	---	12
E	7	---	782.387	1430.749	---	11
N	8	---	896.429	1301.706	0.919	10
E	9	---	1025.472	1187.663	---	9
K#	10	---	1267.610	1058.620	-5.029	8
L	11	---	1380.694	816.483	-0.899	7
K	12	---	1508.789	703.398	3.061	6
E	13	---	1637.832	575.304	-0.832	5
E	14	---	1766.874	446.261	-0.684	4
L	15	---	1879.958	317.218	-0.415	3
G	16	---	1936.980	204.134	0.269	2
K	17	---	---	147.113	0.233	1

+2

Seq	#	b: Δ Error	b	y	y: Δ Error	+1
L	1	---	57.549	---	---	17
M	2	---	123.070	985.504	---	16
A	3	---	158.588	919.984	-3.940	15
L	4	39.661	215.130	884.465	0.665	14
S	5	---	258.646	827.923	-2.485	13
H	6	---	327.176	784.407	4.410	12
E	7	---	391.697	715.878	-4.921	11
N	8	---	448.718	651.357	-15.443	10
E	9	---	513.240	594.335	---	9
K#	10	---	634.309	529.814	1.818	8
L	11	---	690.851	408.745	-1.660	7
K	12	---	754.898	352.203	-8.312	6
E	13	---	819.419	288.155	---	5
E	14	---	883.941	223.634	---	4
L	15	---	940.483	159.113	---	3
G	16	---	968.993	102.571	---	2
K	17	---	---	74.060	---	1

+3

Seq	#	b: Δ Error	b	y	y: Δ Error	+1
L	1	---	38.702	---	---	17
M	2	---	82.382	657.339	3.705	16
A	3	---	106.061	613.658	-0.331	15
L	4	---	143.756	589.979	-1.860	14
S	5	---	172.767	552.285	4.177	13
H	6	---	218.453	523.274	-0.150	12
E	7	---	261.467	477.588	---	11
N	8	---	299.481	434.574	---	10
E	9	---	342.496	396.559	---	9
K#	10	---	423.208	353.545	---	8
L	11	---	460.903	272.832	---	7
K	12	---	503.601	235.138	---	6
E	13	-2.375	546.615	192.439	---	5
E	14	---	589.630	149.425	---	4
L	15	---	627.324	106.411	---	3
G	16	---	646.331	68.716	---	2
K	17	---	---	49.709	---	1

OPTN K193

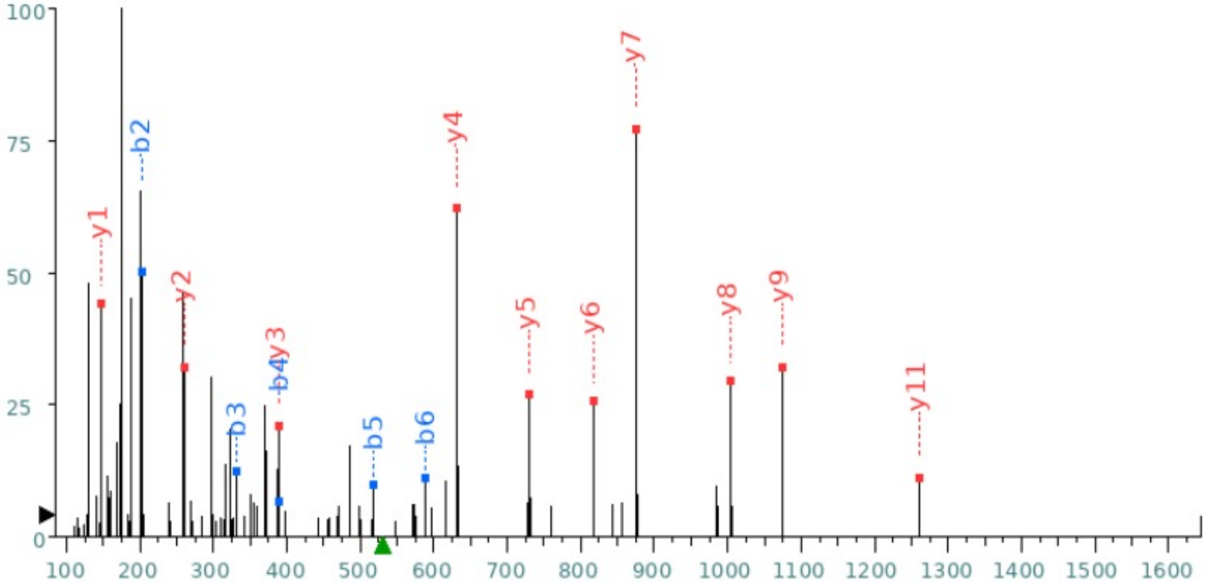

Sequence

MAEGEAEGSVK#EIK

Predicted Fragmentation Pattern

+1

Seq	#	b: Δ Error	b	y	y: Δ Error	+1
M	1	---	132.048	---	---	14
A	2	2.154	203.085	1460.723	---	13
E	3	5.802	332.127	1389.686	---	12
G	4	6.260	389.149	1260.643	-1.030	11
E	5	0.714	518.192	1203.622	---	10
A	6	5.179	589.229	1074.579	0.581	9
E	7	---	718.271	1003.542	-2.231	8
G	8	---	775.293	874.499	2.035	7
S	9	---	862.325	817.478	2.226	6
V	10	---	961.393	730.446	-1.624	5
K#	11	---	1203.531	631.377	0.915	4
E	12	---	1332.574	389.239	3.475	3
I	13	---	1445.658	260.197	1.761	2
K	14	---	---	147.113	2.203	1

+2

Seq	#	b: Δ Error	b	y	y: Δ Error	+1
M	1	---	66.528	---	---	14
A	2	---	102.046	730.865	---	13
E	3	---	166.567	695.346	---	12
G	4	---	195.078	630.825	---	11
E	5	---	259.599	602.314	---	10
A	6	---	295.118	537.793	---	9
E	7	---	359.639	502.275	---	8
G	8	---	388.150	437.753	---	7
S	9	---	431.666	409.243	---	6
V	10	---	481.200	365.727	---	5
K#	11	---	602.269	316.192	---	4
E	12	---	666.790	195.123	---	3
I	13	---	723.332	130.602	---	2
K	14	---	---	74.060	---	1

OPTN K213

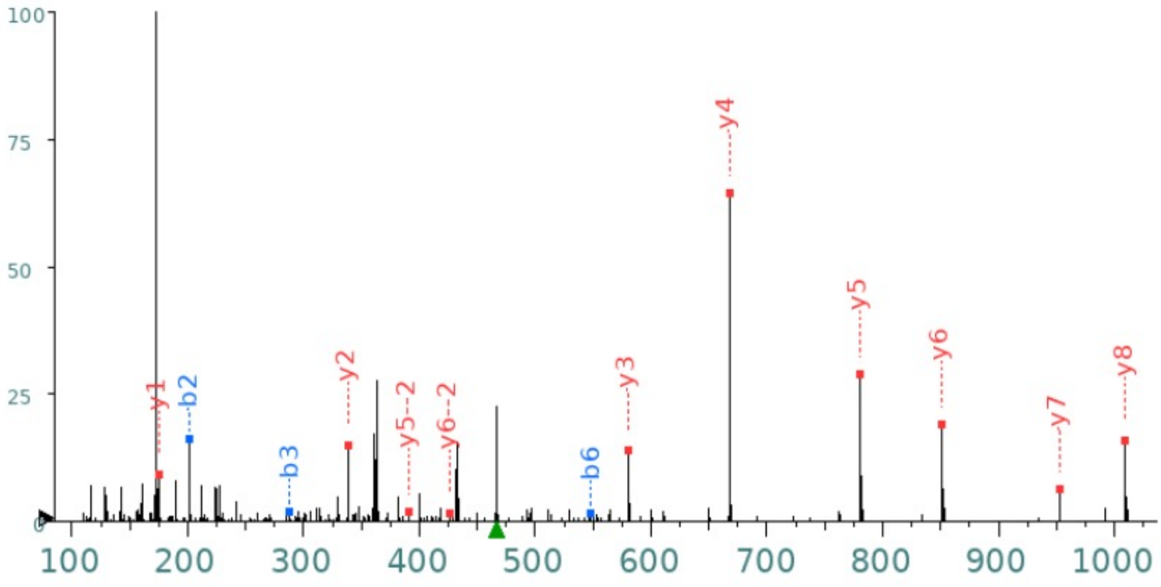

Sequence

TVSTGTALSK#YR

Predicted Fragmentation Pattern

+1

Seq	#	b: Δ Error	b	y	y: Δ Error	+1
T	1	---	102.055	---	---	12
V	2	2.422	**201.123**	1296.691	---	11
S	3	0.949	**288.155**	1197.622	---	10
T	4	---	389.203	1110.590	---	9
G	5	---	446.225	**1009.543**	1.351	8
T	6	21.077	**547.272**	**952.521**	3.910	7
A	7	---	618.309	**851.473**	2.808	6
L	8	---	731.393	**780.436**	1.506	5
S	9	---	818.425	**667.352**	1.331	4
K#	10	---	1060.563	**580.320**	0.979	3
Y	11	---	1223.627	**338.182**	2.257	2
R	12	---	---	**175.119**	2.033	1

+2

Seq	#	b: Δ Error	b	y	y: Δ Error	+1
T	1	---	51.531	---	---	12
V	2	---	101.065	648.849	---	11
S	3	---	144.581	599.315	---	10
T	4	---	195.105	555.799	---	9
G	5	---	223.616	505.275	---	8
T	6	---	274.140	476.764	---	7
A	7	---	309.658	**426.240**	-7.806	6
L	8	---	366.200	**390.722**	4.533	5
S	9	---	409.716	334.180	---	4
K#	10	---	530.785	290.664	---	3
Y	11	---	612.317	169.595	---	2
R	12	---	---	88.063	---	1

OPTN K340

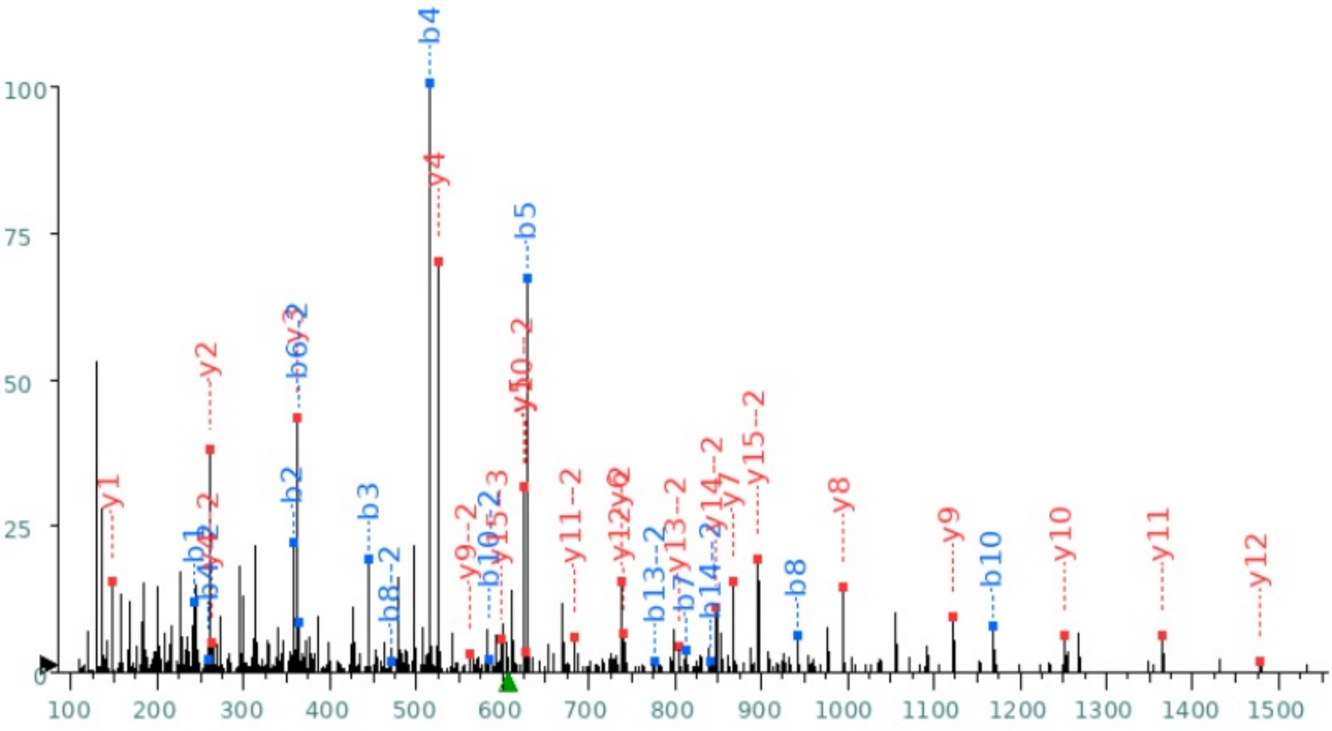

Sequence

K#NSAIPSELNEKQELVYTNK

Predicted Fragmentation Pattern

+1

Seq	#	b: Δ Error	b	y	y: Δ Error	+1
K#	1	-2.926	243.145	---	---	20
N	2	-1.919	357.188	2177.108	---	19
S	3	-1.028	444.220	2063.066	---	18
A	4	1.237	515.257	1976.033	---	17
I	5	1.956	628.341	1904.996	---	16
P	6	---	725.394	1791.912	---	15
S	7	-15.934	812.426	1694.860	---	14
E	8	2.986	941.469	1607.828	---	13
L	9	---	1054.553	1478.785	7.661	12
N	10	-9.274	1168.596	1365.701	3.081	11
E	11	---	1297.638	1251.658	4.694	10
K	12	---	1425.733	1122.615	1.745	9
Q	13	---	1553.792	994.520	1.471	8
E	14	---	1682.834	866.462	0.895	7
L	15	---	1795.918	737.419	-0.617	6
V	16	---	1894.987	624.335	0.670	5
Y	17	---	2058.050	525.267	2.179	4
T	18	---	2159.098	362.203	0.644	3
N	19	---	2273.141	261.156	1.166	2
K	20	---	---	147.113	1.166	1

+2

Seq	#	b: Δ Error	b	y	y: Δ Error	+1
K#	1	---	122.076	---	---	20
N	2	---	179.098	1089.058	---	19
S	3	---	222.614	1032.036	---	18
A	4	2.274	258.132	988.520	---	17
I	5	---	314.674	953.002	---	16
P	6	17.346	363.201	896.460	0.862	15
S	7	---	406.717	847.933	0.209	14
E	8	20.324	471.238	804.417	3.891	13
L	9	---	527.780	739.896	1.502	12
N	10	-8.182	584.801	683.354	-2.603	11
E	11	---	649.323	626.333	7.847	10
K	12	---	713.370	561.811	-0.060	9
Q	13	-9.038	777.400	497.764	---	8
E	14	3.404	841.921	433.735	---	7
L	15	---	898.463	369.213	---	6
V	16	---	947.997	312.671	---	5
Y	17	---	1029.529	263.137	6.209	4
T	18	---	1080.053	181.605	---	3
N	19	---	1137.074	131.082	---	2
K	20	---	---	74.060	---	1

+3

Seq	#	b: Δ Error	b	y	y: Δ Error	+1
K#	1	---	81.720	---	---	20
N	2	---	119.734	726.374	---	19
S	3	---	148.745	688.360	---	18
A	4	---	172.424	659.349	---	17
I	5	---	210.119	635.670	---	16
P	6	---	242.470	597.976	4.019	15
S	7	---	271.480	565.625	---	14
E	8	---	314.494	536.614	---	13
L	9	---	352.189	493.600	---	12
N	10	---	390.203	455.905	---	11
E	11	---	433.218	417.891	---	10
K	12	---	475.916	374.877	---	9
Q	13	---	518.602	332.178	---	8
E	14	---	561.616	289.492	---	7
L	15	---	599.311	246.478	---	6
V	16	---	632.334	208.783	---	5
Y	17	---	686.688	175.760	---	4
T	18	---	720.371	121.406	---	3
N	19	---	758.385	87.723	---	2
K	20	---	---	49.709	---	1

OPTN K351

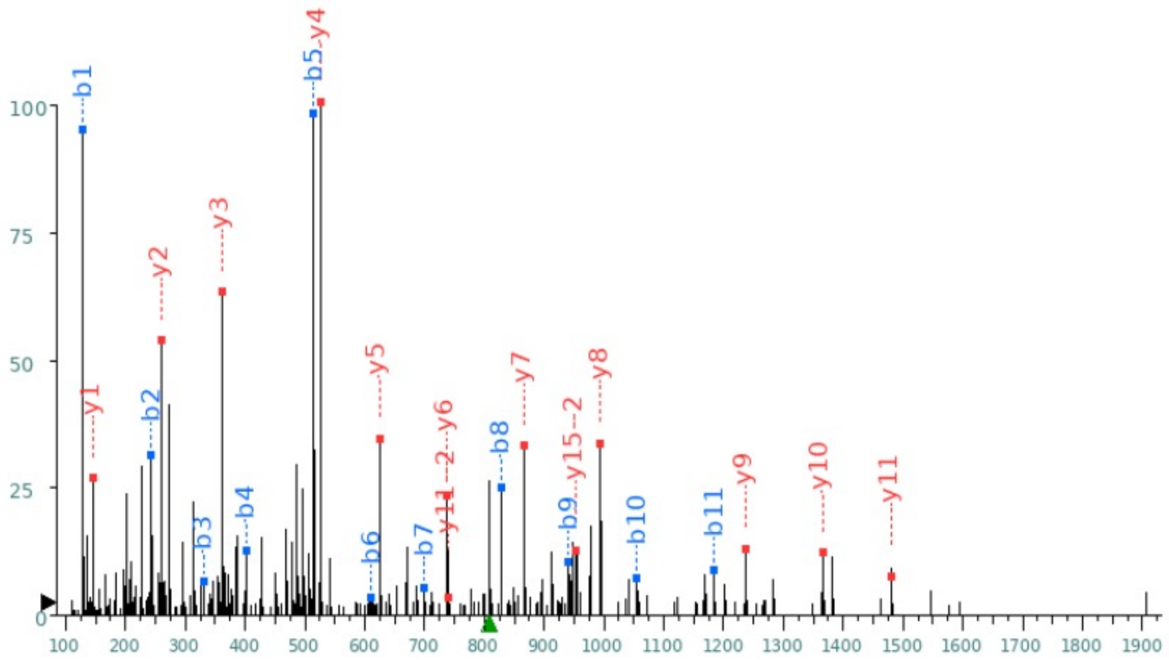

Sequence

KNSAIPSELNEK#QELVYTNK

Predicted Fragmentation Pattern

+1

Seq	#	b: Δ Error	b	y	y: Δ Error	+1
K	1	2.560	129.102	---	---	20
N	2	4.353	243.145	2291.151	---	19
S	3	-4.281	330.177	2177.108	---	18
A	4	1.638	401.214	2090.076	---	17
I	5	2.191	514.298	2019.039	---	16
P	6	3.193	611.351	1905.955	---	15
S	7	-13.439	698.383	1808.902	---	14
E	8	0.913	827.426	1721.870	---	13
L	9	-2.137	940.510	1592.828	---	12
N	10	-5.686	1054.553	1479.744	-6.121	11
E	11	10.206	1183.595	1365.701	-5.411	10
K#	12	---	1425.733	1236.658	6.948	9
Q	13	---	1553.792	994.520	2.944	8
E	14	---	1682.834	866.462	2.163	7
L	15	---	1795.918	737.419	8.571	6
V	16	---	1894.987	624.335	1.061	5
Y	17	---	2058.050	525.267	2.411	4
T	18	---	2159.098	362.203	0.307	3
N	19	---	2273.141	261.156	1.282	2
K	20	---	---	147.113	1.062	1

+2

Seq	#	b: Δ Error	b	y	y: Δ Error	+1
K	1	---	65.055	---	---	20
N	2	---	122.076	1146.079	---	19
S	3	---	165.592	1089.058	---	18
A	4	---	201.111	1045.542	---	17
I	5	---	257.653	1010.023	---	16
P	6	---	306.179	953.481	1.985	15
S	7	---	349.695	904.955	---	14
E	8	---	414.217	861.439	---	13
L	9	---	470.759	796.918	---	12
N	10	---	527.780	740.376	3.149	11
E	11	---	592.301	683.354	---	10
K#	12	---	713.370	618.833	---	9
Q	13	---	777.400	497.764	---	8
E	14	---	841.921	433.735	---	7
L	15	---	898.463	369.213	---	6
V	16	---	947.997	312.671	---	5
Y	17	---	1029.529	263.137	---	4
T	18	---	1080.053	181.605	---	3
N	19	---	1137.074	131.082	---	2
K	20	---	---	74.060	---	1

OPTN K395

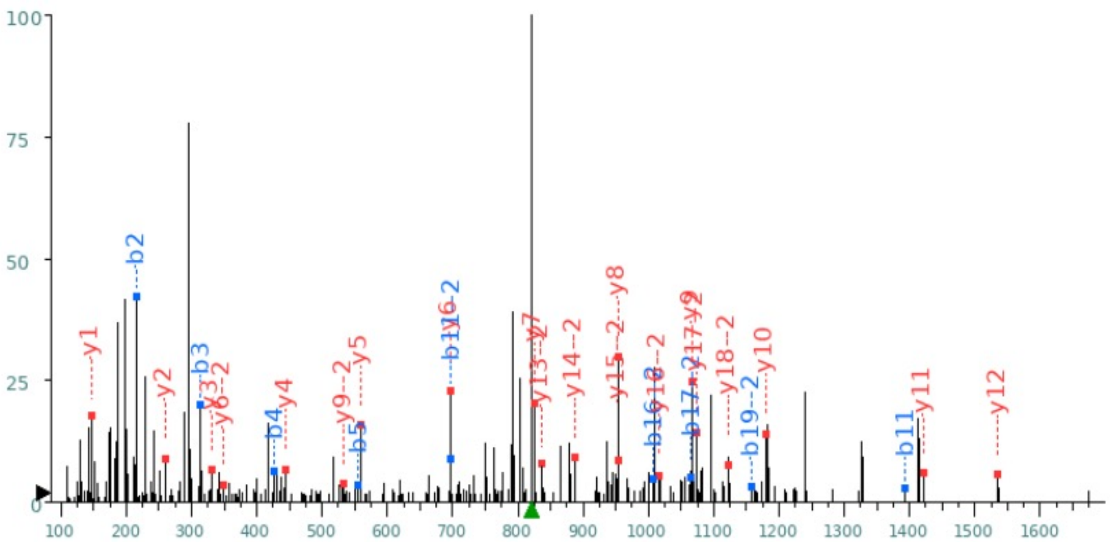

Sequence

LTVLQMTHNK#LLQEHNNALK

Predicted Fragmentation Pattern

+1

Seq	#	b: Δ Error	b	y	y: Δ Error	+1
L	1	---	114.091	---	---	20
T	2	1.155	**215.139**	2346.235	---	19
V	3	1.054	**314.207**	2245.187	---	18
L	4	-0.554	**427.291**	2146.119	---	17
Q	5	6.307	**555.350**	2033.035	---	16
M	6	---	686.391	1904.976	---	15
T	7	---	787.438	1773.935	---	14
H	8	---	924.497	1672.888	---	13
N	9	---	1038.540	**1535.829**	3.015	12
K#	10	---	1280.678	**1421.786**	-8.105	11
L	11	1.265	**1393.762**	**1179.648**	3.435	10
L	12	---	1506.846	**1066.564**	-0.704	9
Q	13	---	1634.905	**953.480**	4.033	8
E	14	---	1763.947	**825.421**	0.128	7
H	15	---	1901.006	**696.379**	2.592	6
N	16	---	2015.049	**559.320**	-1.005	5
N	17	---	2129.092	**445.277**	7.417	4
A	18	---	2200.129	**331.234**	-0.381	3
L	19	---	2313.213	**260.197**	1.878	2
K	20	---	---	**147.113**	0.855	1

+2

Seq	#	b: Δ Error	b	y	y: Δ Error	+1
L	1	---	57.549	---	---	20
T	2	---	108.073	1173.621	---	19
V	3	---	157.607	**1123.097**	4.159	18
L	4	---	214.149	**1073.563**	-0.286	17
Q	5	---	278.179	**1017.021**	-0.323	16
M	6	---	343.699	**952.992**	3.042	15
T	7	---	394.223	**887.471**	-1.228	14
H	8	---	462.752	**836.948**	7.637	13
N	9	---	519.774	768.418	---	12
K#	10	---	640.843	711.397	---	11
L	11	-1.764	**697.385**	590.328	---	10
L	12	---	753.927	**533.786**	-4.432	9
Q	13	---	817.956	477.244	---	8
E	14	---	882.477	413.214	---	7
H	15	---	951.007	**348.693**	2.833	6
N	16	-3.686	**1008.028**	280.164	---	5
N	17	3.809	**1065.050**	223.142	---	4
A	18	---	1100.568	166.121	---	3
L	19	-0.856	**1157.110**	130.602	---	2
K	20	---	---	74.060	---	1

OPTN K424

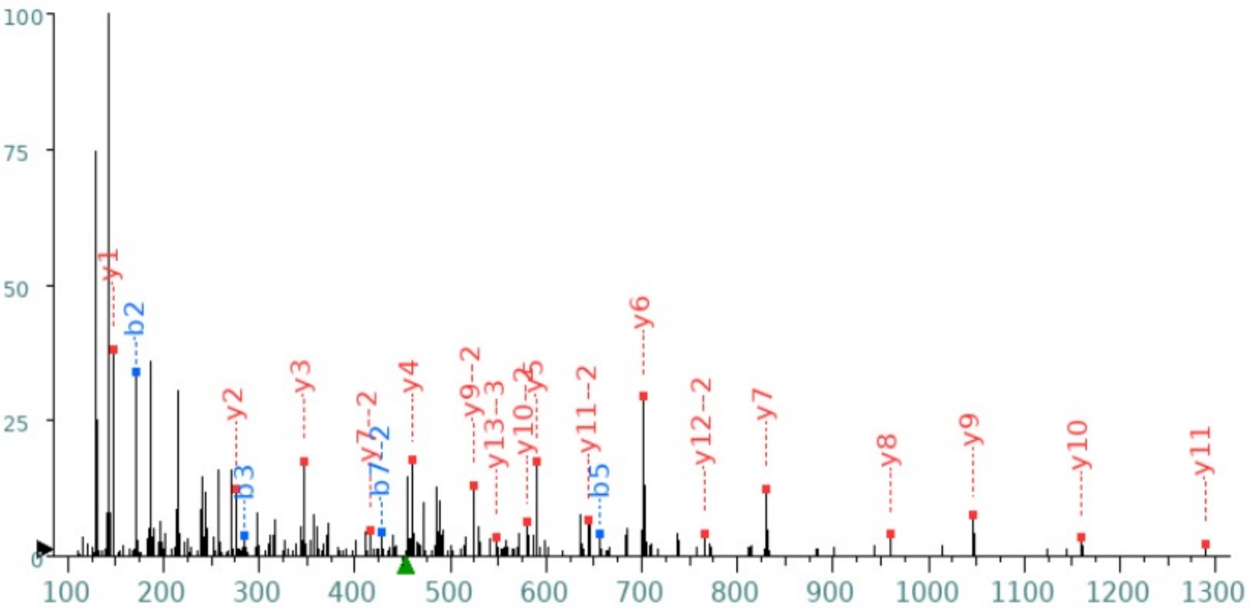

Sequence

AVLK#ELSEKLELAEK

Predicted Fragmentation Pattern

+1

Seq	#	b: Δ Error	b	y	y: Δ Error	+1
A	1	---	72.044	---	---	15
V	2	1.453	**171.113**	1742.990	---	14
L	3	3.226	**284.197**	1643.921	---	13
K#	4	---	526.335	1530.837	---	12
E	5	1.907	**655.377**	**1288.699**	-1.998	11
L	6	---	768.461	**1159.657**	3.456	10
S	7	---	855.493	**1046.573**	5.188	9
E	8	---	984.536	**959.541**	5.262	8
K	9	---	1112.631	**830.498**	2.834	7
L	10	---	1225.715	**702.403**	0.473	6
E	11	---	1354.758	**589.319**	2.770	5
L	12	---	1467.842	**460.277**	1.139	4
A	13	---	1538.879	**347.193**	0.597	3
E	14	---	1667.921	**276.155**	1.208	2
K	15	---	---	**147.113**	1.996	1

+2

Seq	#	b: Δ Error	b	y	y: Δ Error	+1
A	1	---	36.526	---	---	15
V	2	---	86.060	871.999	---	14
L	3	---	142.602	822.464	---	13
K#	4	---	263.671	**765.922**	10.734	12
E	5	---	328.192	**644.853**	2.868	11
L	6	---	384.734	**580.332**	7.701	10
S	7	7.284	**428.250**	**523.790**	-0.597	9
E	8	---	492.772	480.274	---	8
K	9	---	556.819	**415.753**	-1.002	7
L	10	---	613.361	351.705	---	6
E	11	---	677.882	295.163	---	5
L	12	---	734.424	230.642	---	4
A	13	---	769.943	174.100	---	3
E	14	---	834.464	138.581	---	2
K	15	---	---	74.060	---	1

+3

Seq	#	b: Δ Error	b	y	y: Δ Error	+1
A	1	---	24.686	---	---	15
V	2	---	57.709	581.668	---	14
L	3	---	95.404	**548.645**	2.444	13
K#	4	---	176.116	510.951	---	12
E	5	---	219.131	430.238	---	11
L	6	---	256.825	387.224	---	10
S	7	---	285.836	349.529	---	9
E	8	---	328.850	320.518	---	8
K	9	---	371.549	277.504	---	7
L	10	---	409.243	234.806	---	6
E	11	---	452.257	197.111	---	5
L	12	---	489.952	154.097	---	4
A	13	---	513.631	116.402	---	3
E	14	---	556.645	92.723	---	2
K	15	---	---	49.709	---	1

OPTN K435

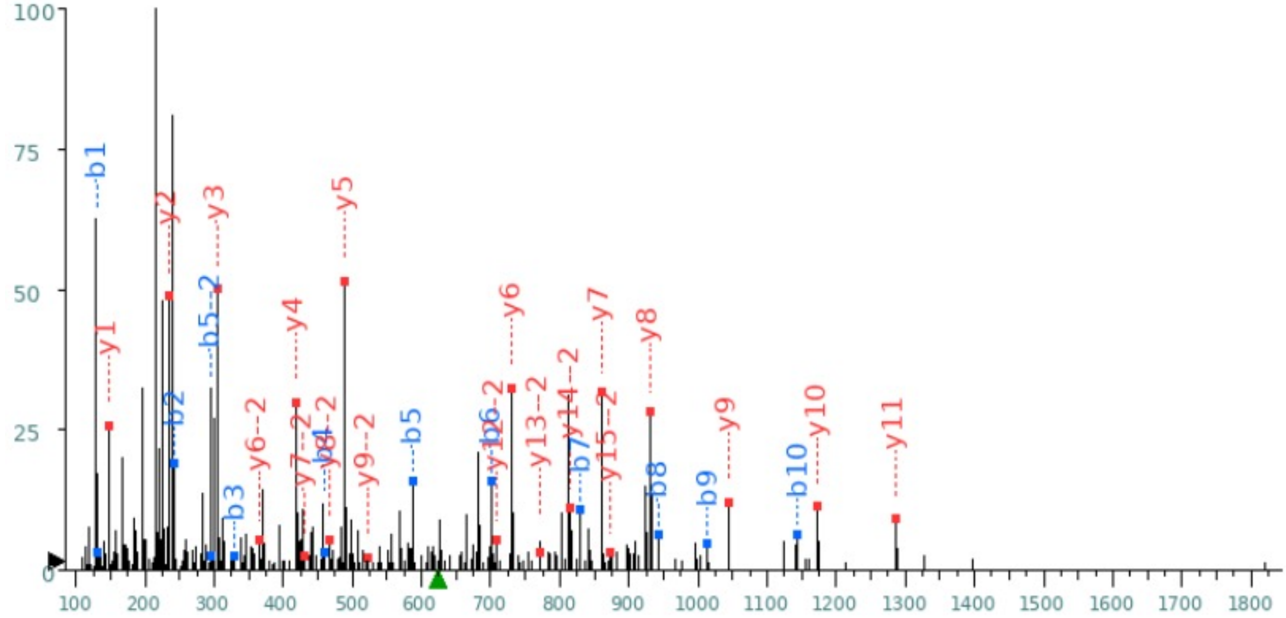

Sequence

ELSEKLELAEK#ALASK

Predicted Fragmentation Pattern

+1

Seq	#	b: Δ Error	b	y	y: Δ Error	+1
E	1	1.030	130.050	---	---	16
L	2	-1.283	243.134	1743.985	---	15
S	3	3.122	330.166	1630.901	---	14
E	4	7.116	459.209	1543.869	---	13
K	5	-1.541	587.304	1414.826	---	12
L	6	1.644	700.388	1286.731	1.045	11
E	7	-0.072	829.430	1173.647	4.958	10
L	8	5.033	942.514	1044.605	4.159	9
A	9	12.143	1013.551	931.521	2.653	8
E	10	7.414	1142.594	860.484	1.246	7
K#	11	---	1384.732	731.441	1.036	6
A	12	---	1455.769	489.303	2.820	5
L	13	---	1568.853	418.266	0.391	4
A	14	---	1639.890	305.182	1.597	3
S	15	---	1726.922	234.145	1.579	2
K	16	---	---	147.113	1.477	1

+2

Seq	#	b: Δ Error	b	y	y: Δ Error	+1
E	1	---	65.529	---	---	16
L	2	---	122.071	872.496	-1.642	15
S	3	---	165.587	815.954	0.013	14
E	4	---	230.108	772.438	-9.122	13
K	5	-27.394	294.155	707.917	5.904	12
L	6	---	350.697	643.869	---	11
E	7	---	415.219	587.327	---	10
L	8	---	471.761	522.806	-1.986	9
A	9	---	507.279	466.264	-6.134	8
E	10	---	571.801	430.745	-2.242	7
K#	11	---	692.870	366.224	-4.233	6
A	12	---	728.388	245.155	---	5
L	13	---	784.930	209.637	---	4
A	14	---	820.449	153.095	---	3
S	15	---	863.965	117.576	---	2
K	16	---	---	74.060	---	1

OPTN K453

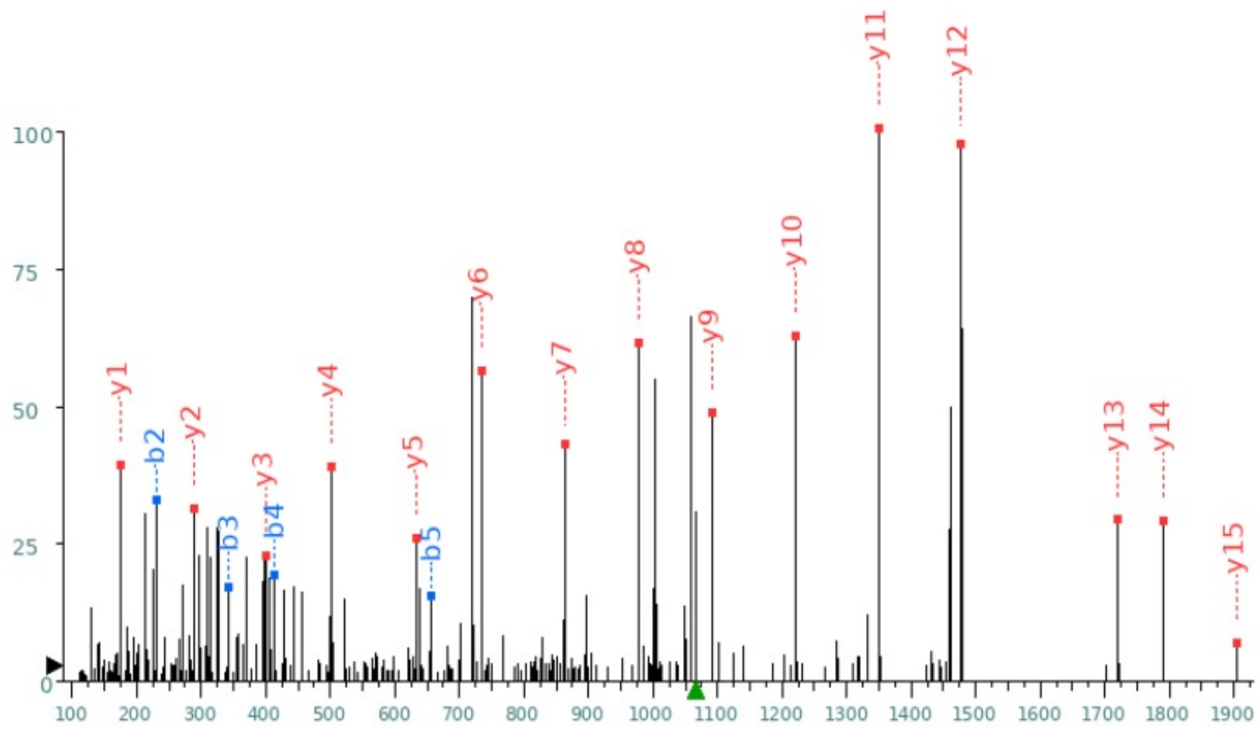

Sequence

QTIAK#QEEDLETMTILR

Predicted Fragmentation Pattern

Seq	#	b: Δ Error	b	y	y: Δ Error	+1
Q	1	---	129.066	---	---	17
T	2	2.160	230.114	2005.027	---	16
I	3	4.373	343.198	1903.979	-3.582	15
A	4	1.476	414.235	1790.895	-0.765	14
K#	5	5.888	656.373	1719.858	5.381	13
Q	6	---	784.431	1477.720	3.256	12
E	7	---	913.474	1349.662	2.830	11
E	8	---	1042.516	1220.619	3.621	10
D	9	---	1157.543	1091.577	2.810	9
L	10	---	1270.627	976.550	2.731	8
E	11	---	1399.670	863.466	2.121	7
T	12	---	1500.718	734.423	1.566	6
M	13	---	1631.758	633.375	2.795	5
T	14	---	1732.806	502.335	3.379	4
I	15	---	1845.890	401.287	1.745	3
L	16	---	1958.974	288.203	2.177	2
R	17	---	---	175.119	2.643	1

OPTN K501

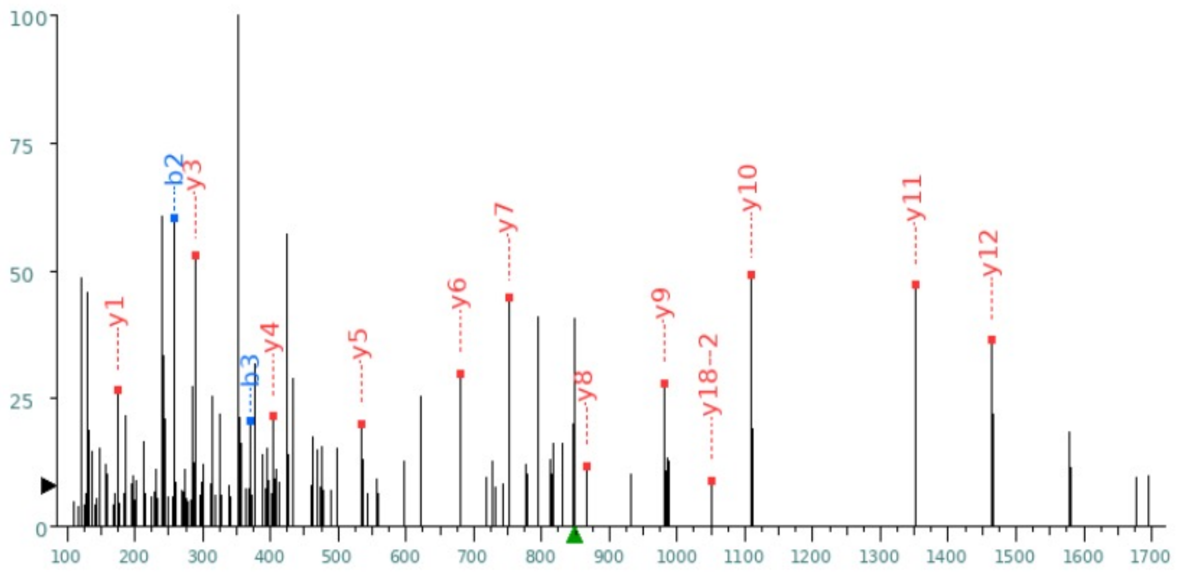

Sequence

EQLALQLAVLLK#ENDAFEDGGR

Predicted Fragmentation Pattern

+1

Seq	#	b: Δ Error	b	y	y: Δ Error	+1
E	1	---	130.050	---	---	22
Q	2	-0.305	**258.108**	2414.267	---	21
L	3	5.246	**371.193**	2286.209	---	20
A	4	---	442.230	2173.125	---	19
L	5	---	555.314	2102.088	---	18
Q	6	---	683.372	1989.004	---	17
L	7	---	796.456	1860.945	---	16
A	8	---	867.493	1747.861	---	15
V	9	---	966.562	1676.824	---	14
L	10	---	1079.646	1577.755	---	13
L	11	---	1192.730	**1464.671**	7.434	12
K#	12	---	1434.868	**1351.587**	1.161	11
E	13	---	1563.910	**1109.449**	-3.251	10
N	14	---	1677.953	**980.407**	-5.992	9
D	15	---	1792.980	**866.364**	0.146	8
A	16	---	1864.017	**751.337**	1.098	7
F	17	---	2011.086	**680.300**	-5.690	6
E	18	---	2140.128	**533.231**	1.772	5
D	19	---	2255.155	**404.189**	5.410	4
G	20	---	2312.177	**289.162**	-0.051	3
G	21	---	2369.198	232.140	---	2
R	22	---	---	**175.119**	3.601	1

+2

Seq	#	b: Δ Error	b	y	y: Δ Error	+1
E	1	---	65.529	---	---	22
Q	2	---	129.558	1207.637	---	21
L	3	---	186.100	1143.608	---	20
A	4	---	221.618	1087.066	---	19
L	5	---	278.160	**1051.547**	8.143	18
Q	6	---	342.190	995.005	---	17
L	7	---	398.732	930.976	---	16
A	8	---	434.250	874.434	---	15
V	9	---	483.785	838.916	---	14
L	10	---	540.327	789.381	---	13
L	11	---	596.869	732.839	---	12
K#	12	---	717.938	676.297	---	11
E	13	---	782.459	555.228	---	10
N	14	---	839.480	490.707	---	9
D	15	---	896.994	433.686	---	8
A	16	---	932.512	376.172	---	7
F	17	---	1006.047	340.654	---	6
E	18	---	1070.568	267.119	---	5
D	19	---	1128.081	202.598	---	4
G	20	---	1156.592	145.085	---	3
G	21	---	1185.103	116.574	---	2
R	22	---	---	88.063	---	1

OPTN-Ub K63

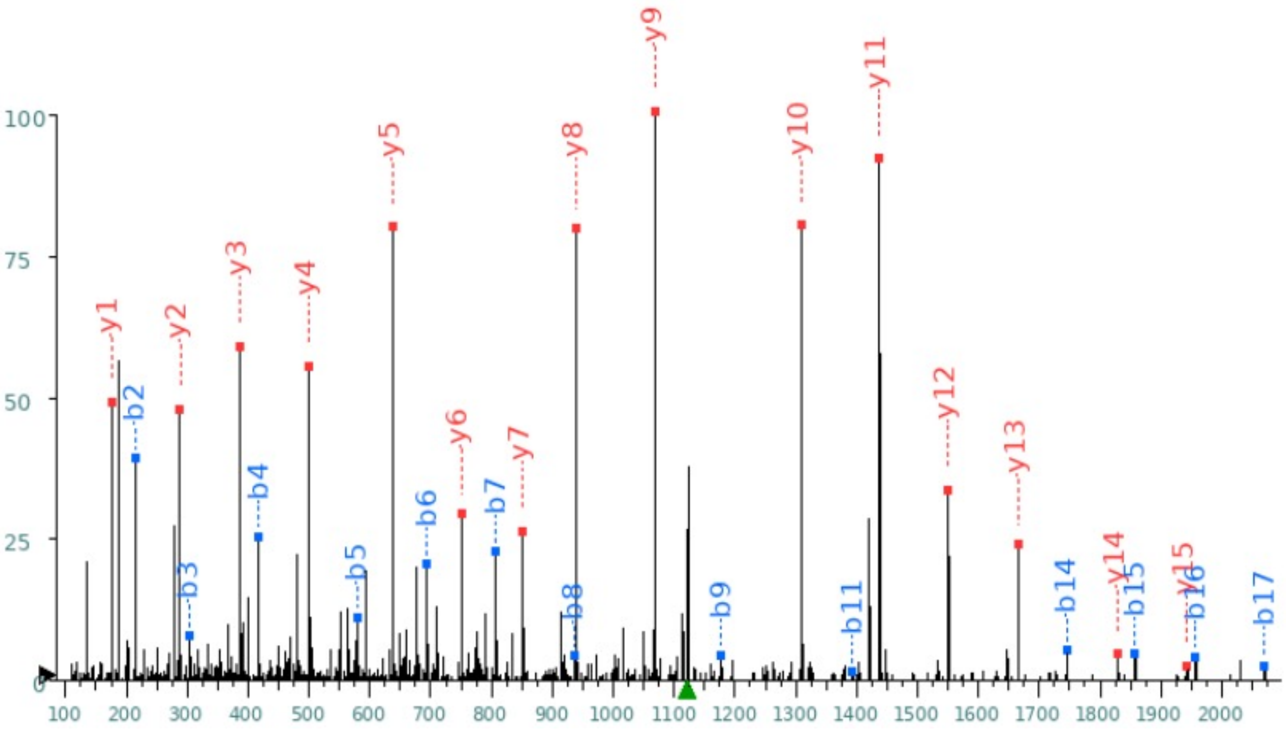

Sequence

TLSDYNIQK#ESTLHLVLR

Predicted Fragmentation Pattern

Seq	#	b: Δ Error	b	y	y: Δ Error	+1
T	1	---	102.055	---	---	18
L	2	-1.682	215.139	2143.151	---	17
S	3	-3.117	302.171	2030.067	---	16
D	4	-3.053	417.198	1943.035	-6.134	15
Y	5	-1.940	580.261	1828.008	3.411	14
N	6	-2.441	694.304	1664.944	-0.963	13
I	7	-2.349	807.388	1550.901	-1.375	12
Q	8	-1.618	935.447	1437.817	-0.833	11
K#	9	-4.406	1177.585	1309.759	-2.139	10
E	10	---	1306.627	1067.621	-1.298	9
S	11	-1.822	1393.659	938.578	-0.706	8
T	12	---	1494.707	851.546	-2.086	7
L	13	---	1607.791	750.498	-1.784	6
H	14	3.873	1744.850	637.414	-2.071	5
L	15	-4.815	1857.934	500.355	-1.637	4
V	16	1.201	1957.003	387.271	-2.382	3
L	17	6.640	2070.087	288.203	-2.271	2
R	18	---	---	175.119	-1.714	1

OPTN-Ub K11

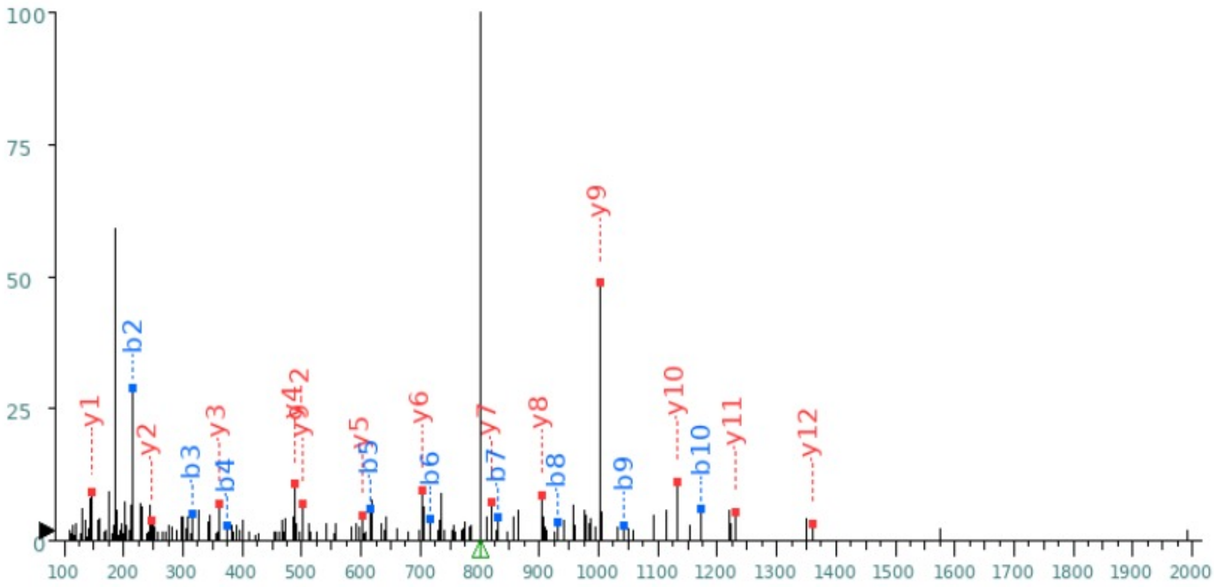

Sequence

TLTGK#TITLEVEPSDTIENVK

Predicted Fragmentation Pattern

+1

Seq	#	b: Δ Error	b	y	y: Δ Error	+1
T	1	---	102.055	---	---	21
L	2	3.212	215.139	2301.218	---	20
T	3	-1.224	316.187	2188.134	---	19
G	4	22.568	373.208	2087.087	---	18
K#	5	-1.061	615.346	2030.065	---	17
T	6	10.917	716.394	1787.927	---	16
I	7	-8.401	829.478	1686.880	---	15
T	8	-5.794	930.525	1573.796	---	14
L	9	-0.154	1043.610	1472.748	---	13
E	10	-5.022	1172.652	1359.664	-5.924	12
V	11	---	1271.721	1230.621	0.986	11
E	12	---	1400.763	1131.553	4.141	10
P	13	---	1497.816	1002.510	3.020	9
S	14	---	1584.848	905.457	1.691	8
D	15	---	1699.875	818.425	-0.384	7
T	16	---	1800.923	703.398	-2.319	6
I	17	---	1914.007	602.351	-3.805	5
E	18	---	2043.049	489.267	4.210	4
N	19	---	2157.092	360.224	1.626	3
V	20	---	2256.161	246.181	2.459	2
K	21	---	---	147.113	2.515	1

+2

Seq	#	b: Δ Error	b	y	y: Δ Error	+1
T	1	---	51.531	---	---	21
L	2	---	108.073	1151.113	---	20
T	3	---	158.597	1094.571	---	19
G	4	---	187.108	1044.047	---	18
K#	5	---	308.177	1015.536	---	17
T	6	---	358.701	894.467	---	16
I	7	---	415.243	843.943	---	15
T	8	---	465.766	787.401	---	14
L	9	---	522.308	736.878	---	13
E	10	---	586.830	680.336	---	12
V	11	---	636.364	615.814	---	11
E	12	---	700.885	566.280	---	10
P	13	---	749.412	501.759	3.978	9
S	14	---	792.928	453.232	---	8
D	15	---	850.441	409.716	---	7
T	16	---	900.965	352.203	---	6
I	17	---	957.507	301.679	---	5
E	18	---	1022.028	245.137	---	4
N	19	---	1079.050	180.616	---	3
V	20	---	1128.584	123.594	---	2
K	21	---	---	74.060	---	1

SQSTM1 K295

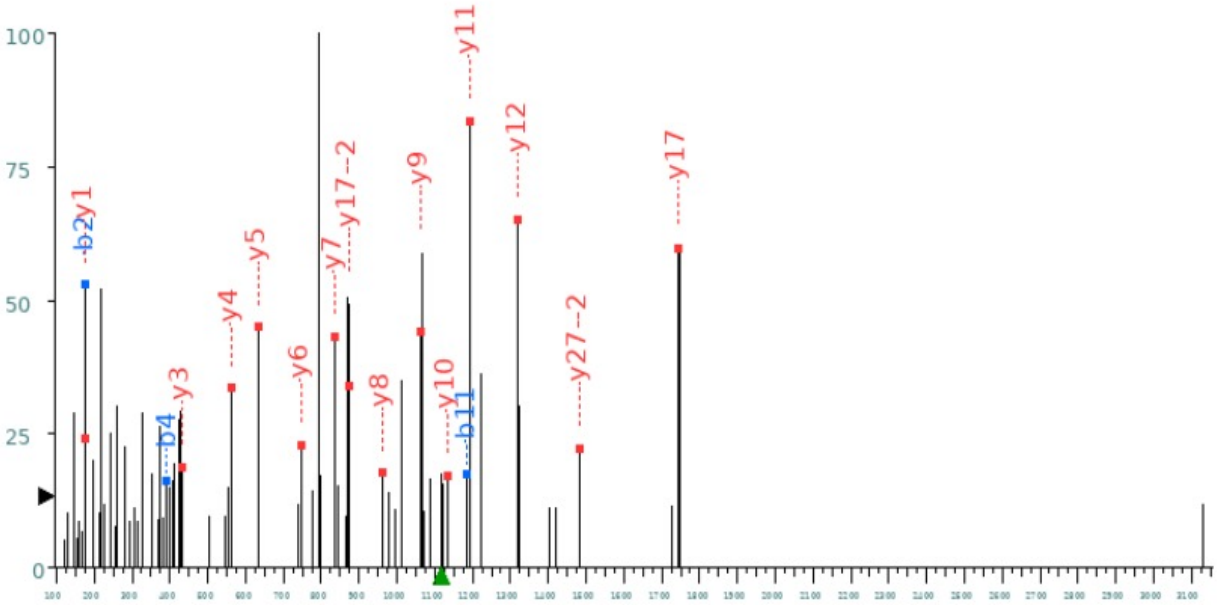

Sequence

SSSQPSSCCSDPSK$^{\#}$PGGNVEGATQSLAEQMR

Predicted Fragmentation Pattern

+1

Seq	#	b: Δ Error	b	y	y: Δ Error	+1
S	1	---	88.039	---	---	31
S	2	4.282	**175.071**	3266.411	---	30
S	3	---	262.103	3179.379	---	29
Q	4	-1.440	**390.162**	3092.347	---	28
P	5	---	487.215	2964.288	---	27
S	6	---	574.247	2867.235	---	26
S	7	---	661.279	2780.203	---	25
C	8	---	821.309	2693.171	---	24
C	9	---	981.340	2533.141	---	23
S	10	---	1068.372	2373.110	---	22
D	11	-8.751	**1183.399**	2286.078	---	21
P	12	---	1280.452	2171.051	---	20
S	13	---	1367.484	2073.998	---	19
K$^{\#}$	14	---	1609.622	1986.966	---	18
P	15	---	1706.674	**1744.828**	-6.731	17
G	16	---	1763.696	1647.776	---	16
G	17	---	1820.717	1590.754	---	15
N	18	---	1934.760	1533.733	---	14
V	19	---	2033.829	1419.690	---	13
E	20	---	2162.871	**1320.621**	1.830	12
G	21	---	2219.893	**1191.579**	2.532	11
A	22	---	2290.930	**1134.557**	9.850	10
T	23	---	2391.978	**1063.520**	2.019	9
Q	24	---	2520.036	**962.472**	13.021	8
S	25	---	2607.068	**834.414**	11.636	7
L	26	---	2720.152	**747.382**	7.254	6
A	27	---	2791.189	**634.298**	0.975	5
E	28	---	2920.232	**563.261**	4.681	4
Q	29	---	3048.291	**434.218**	6.613	3
M	30	---	3179.331	306.159	---	2
R	31	---	---	**175.119**	3.950	1

+2

Seq	#	b: Δ Error	b	y	y: Δ Error	+1
S	1	---	44.523	---	---	31
S	2	---	88.039	1633.709	---	30
S	3	---	131.555	1590.193	---	29
Q	4	---	195.585	1546.677	---	28
P	5	---	244.111	**1482.648**	-4.827	27
S	6	---	287.627	1434.121	---	26
S	7	---	331.143	1390.605	---	25
C	8	---	411.158	1347.089	---	24
C	9	---	491.174	1267.074	---	23
S	10	---	534.690	1187.059	---	22
D	11	---	592.203	1143.543	---	21
P	12	---	640.730	1086.029	---	20
S	13	---	684.246	1037.503	---	19
K$^{\#}$	14	---	805.314	993.987	---	18
P	15	---	853.841	**872.918**	1.901	17
G	16	---	882.352	824.391	---	16
G	17	---	910.862	795.881	---	15
N	18	---	967.884	767.370	---	14
V	19	---	1017.418	710.348	---	13
E	20	---	1081.939	660.814	---	12
G	21	---	1110.450	596.293	---	11
A	22	---	1145.969	567.782	---	10
T	23	---	1196.492	532.264	---	9
Q	24	---	1260.522	481.740	---	8
S	25	---	1304.038	417.711	---	7
L	26	---	1360.580	374.195	---	6
A	27	---	1396.098	317.652	---	5
E	28	---	1460.620	282.134	---	4
Q	29	---	1524.649	217.613	---	3
M	30	---	1590.169	153.583	---	2
R	31	---	---	88.063	---	1

SQSTM1 K378

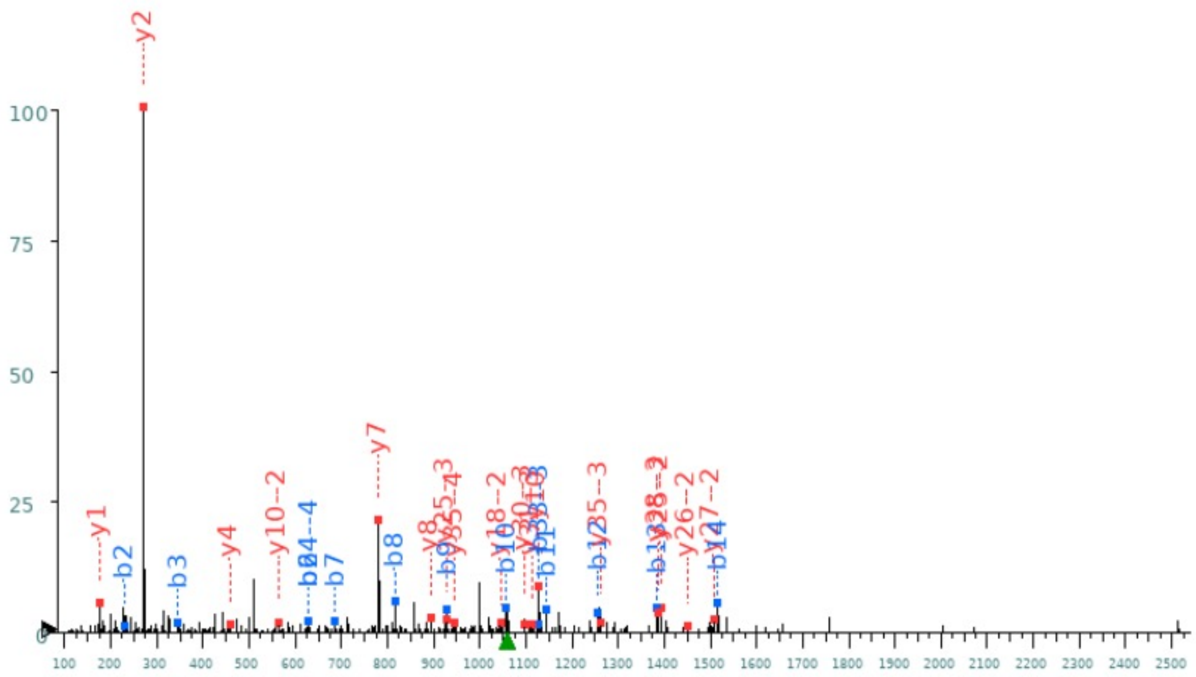

Sequence

EVDPSTGELQSLQMPESEGPSSLDPSQEGPTGLK#EAALYPHLPPEADPR

Predicted Fragmentation Pattern

+1

Seq	#	b: Δ Error	b	y	y: Δ Error	+1
E	1	---	130.050	---	---	49
V	2	-1.651	229.118	5168.464	---	48
D	3	0.287	344.145	5069.396	---	47
P	4	---	441.198	4954.369	---	46
S	5	---	528.230	4857.316	---	45
T	6	11.272	629.278	4770.284	---	44
G	7	5.474	686.299	4669.236	---	43
E	8	2.374	815.342	4612.215	---	42
L	9	2.525	928.426	4483.172	---	41
Q	10	8.069	1056.484	4370.088	---	40
S	11	7.201	1143.516	4242.030	---	39
L	12	-3.516	1256.600	4154.998	---	38
Q	13	8.899	1384.659	4041.913	---	37
M	14	6.788	1515.700	3913.855	---	36
P	15	---	1612.752	3782.814	---	35
E	16	---	1741.795	3685.762	---	34
S	17	---	1828.827	3556.719	---	33
E	18	---	1957.870	3469.687	---	32
G	19	---	2014.891	3340.644	---	31
P	20	---	2111.944	3283.623	---	30
S	21	---	2198.976	3186.570	---	29
S	22	---	2286.008	3099.538	---	28
L	23	---	2399.092	3012.506	---	27
D	24	---	2514.119	2899.422	---	26
P	25	---	2611.172	2784.395	---	25
S	26	---	2698.204	2687.342	---	24
Q	27	---	2826.262	2600.310	---	23
E	28	---	2955.305	2472.252	---	22
G	29	---	3012.326	2343.209	---	21
P	30	---	3109.379	2286.188	---	20
T	31	---	3210.427	2189.135	---	19
G	32	---	3267.448	2088.087	---	18
L	33	---	3380.532	2031.066	---	17
K#	34	---	3622.670	1917.982	---	16
E	35	---	3751.713	1675.844	---	15
A	36	---	3822.750	1546.801	---	14
A	37	---	3893.787	1475.764	---	13
L	38	---	4006.871	1404.727	---	12
Y	39	---	4169.934	1291.643	---	11
P	40	---	4266.987	1128.580	5.261	10
H	41	---	4404.046	1031.527	---	9
L	42	---	4517.130	894.468	7.163	8
P	43	---	4614.183	781.384	4.006	7
P	44	---	4711.236	684.331	---	6
E	45	---	4840.278	587.278	---	5
A	46	---	4911.315	458.236	12.200	4
D	47	---	5026.342	387.199	---	3
P	48	---	5123.395	272.172	3.386	2
R	49	---	---	175.119	3.950	1

+2

Seq	#	b: Δ Error	b	y	y: Δ Error	+1
E	1	---	65.529	---	---	49
V	2	---	115.063	2584.736	---	48
D	3	---	172.576	2535.201	---	47
P	4	---	221.103	2477.688	---	46
S	5	---	264.619	2429.162	---	45
T	6	---	315.142	2385.646	---	44
G	7	---	343.653	2335.122	---	43
E	8	---	408.175	2306.611	---	42
L	9	---	464.717	2242.090	---	41
Q	10	---	528.746	2185.548	---	40
S	11	---	572.262	2121.518	---	39
L	12	---	628.804	2078.002	---	38
Q	13	---	692.833	2021.460	---	37
M	14	---	758.353	1957.431	---	36
P	15	---	806.880	1891.911	---	35
E	16	---	871.401	1843.384	---	34
S	17	---	914.917	1778.863	---	33
E	18	---	979.438	1735.347	---	32
G	19	---	1007.949	1670.826	---	31
P	20	---	1056.476	1642.315	---	30
S	21	---	1099.992	1593.789	---	29
S	22	---	1143.508	1550.273	---	28
L	23	---	1200.050	1506.757	-2.589	27
D	24	---	1257.563	1450.215	8.196	26
P	25	---	1306.089	1392.701	2.693	25
S	26	---	1349.605	1344.175	---	24
Q	27	---	1413.635	1300.659	---	23
E	28	---	1478.156	1236.630	---	22
G	29	---	1506.667	1172.108	---	21
P	30	---	1555.193	1143.597	---	20
T	31	---	1605.717	1095.071	---	19
G	32	---	1634.228	1044.547	-4.703	18
L	33	---	1690.770	1016.037	---	17
K#	34	---	1811.839	959.495	---	16
E	35	---	1876.360	838.426	---	15
A	36	---	1911.879	773.904	---	14
A	37	---	1947.397	738.386	---	13
L	38	---	2003.939	702.867	---	12
Y	39	---	2085.471	646.325	---	11
P	40	---	2133.997	564.793	-0.645	10
H	41	---	2202.527	516.267	---	9
L	42	---	2259.069	447.738	---	8
P	43	---	2307.595	391.196	---	7
P	44	---	2356.121	342.669	---	6
E	45	---	2420.643	294.143	---	5
A	46	---	2456.161	229.622	---	4
D	47	---	2513.675	194.103	---	3
P	48	---	2562.201	136.589	---	2
R	49	---	---	88.063	---	1

+3

Seq	#	b: Δ Error	b	y	y: Δ Error	+1
E	1	---	44.021	---	---	49
V	2	---	77.044	1723.493	---	48
D	3	---	115.387	1690.470	---	47
P	4	---	147.738	1652.128	---	46
S	5	---	176.748	1619.777	---	45
T	6	---	210.431	1590.766	---	44
G	7	---	229.438	1557.084	---	43
E	8	---	272.452	1538.076	---	42
L	9	---	310.147	1495.062	---	41
Q	10	---	352.833	1457.368	---	40
S	11	---	381.844	1414.681	---	39
L	12	---	419.538	1385.671	-2.936	38
Q	13	---	462.225	1347.976	---	37
M	14	---	505.905	1305.290	---	36
P	15	---	538.256	1261.610	2.586	35
E	16	---	581.270	1229.259	---	34
S	17	---	610.280	1186.245	---	33
E	18	---	653.295	1157.234	---	32
G	19	---	672.302	1114.220	2.252	31
P	20	---	704.653	1095.213	5.257	30
S	21	---	733.663	1062.862	---	29
S	22	---	762.674	1033.851	---	28
L	23	---	800.369	1004.840	---	27
D	24	---	838.711	967.146	---	26
P	25	---	871.062	928.803	13.793	25
S	26	---	900.073	896.452	---	24
Q	27	---	942.759	867.442	---	23
E	28	---	985.773	824.755	---	22
G	29	---	1004.780	781.741	---	21
P	30	---	1037.131	762.734	---	20
T	31	---	1070.814	730.383	---	19
G	32	---	1089.821	696.701	---	18
L	33	5.445	1127.516	677.693	---	17
K#	34	---	1208.228	639.999	---	16
E	35	---	1251.242	559.286	---	15
A	36	---	1274.921	516.272	---	14
A	37	---	1298.600	492.593	---	13
L	38	---	1336.295	468.914	---	12
Y	39	---	1390.650	431.219	---	11
P	40	---	1423.001	376.865	---	10
H	41	---	1468.687	344.514	---	9
L	42	---	1506.382	298.828	---	8
P	43	---	1538.732	261.133	---	7
P	44	---	1571.083	228.782	---	6
E	45	---	1614.098	196.431	---	5
A	46	---	1637.777	153.417	---	4
D	47	---	1676.119	129.738	---	3
P	48	---	1708.470	91.395	---	2
R	49	---	---	59.045	---	1

+4

Seq	#	b: Δ Error	b	y	y: Δ Error	+1
E	1	---	33.268	---	---	49
V	2	---	58.035	1292.871	---	48
D	3	---	86.792	1268.104	---	47
P	4	---	111.055	1239.348	---	46
S	5	---	132.813	1215.084	---	45
T	6	---	158.075	1193.326	---	44
G	7	---	172.330	1168.065	---	43
E	8	---	204.591	1153.809	---	42
L	9	---	232.862	1121.549	---	41
Q	10	---	264.877	1093.277	---	40
S	11	---	286.635	1061.263	---	39
L	12	---	314.906	1039.505	---	38
Q	13	---	346.920	1011.234	---	37
M	14	---	379.680	979.219	---	36
P	15	---	403.944	946.459	4.372	35
E	16	---	436.204	922.196	---	34
S	17	---	457.962	889.935	---	33
E	18	---	490.223	868.177	---	32
G	19	---	504.478	835.917	---	31
P	20	---	528.741	821.661	---	30
S	21	---	550.499	797.398	---	29
S	22	---	572.257	775.640	---	28
L	23	---	600.528	753.882	---	27
D	24	-0.591	629.285	725.611	---	26
P	25	---	653.548	696.854	---	25
S	26	---	675.306	672.591	---	24
Q	27	---	707.321	650.833	---	23
E	28	---	739.582	618.818	---	22
G	29	---	753.837	586.558	---	21
P	30	---	778.100	572.302	---	20
T	31	---	803.362	548.039	---	19
G	32	---	817.617	522.777	---	18
L	33	---	845.889	508.522	---	17
K#	34	---	906.423	480.251	---	16
E	35	---	938.684	419.716	---	15
A	36	---	956.443	387.456	---	14
A	37	---	974.202	369.696	---	13
L	38	---	1002.473	351.937	---	12
Y	39	---	1043.239	323.666	---	11
P	40	---	1067.502	282.900	---	10
H	41	---	1101.767	258.637	---	9
L	42	---	1130.038	224.372	---	8
P	43	---	1154.301	196.101	---	7
P	44	---	1178.564	171.838	---	6
E	45	---	1210.825	147.575	---	5
A	46	---	1228.584	115.314	---	4
D	47	---	1257.341	97.555	---	3
P	48	---	1281.604	68.798	---	2
R	49	---	---	44.535	---	1

SQSTM1 K420

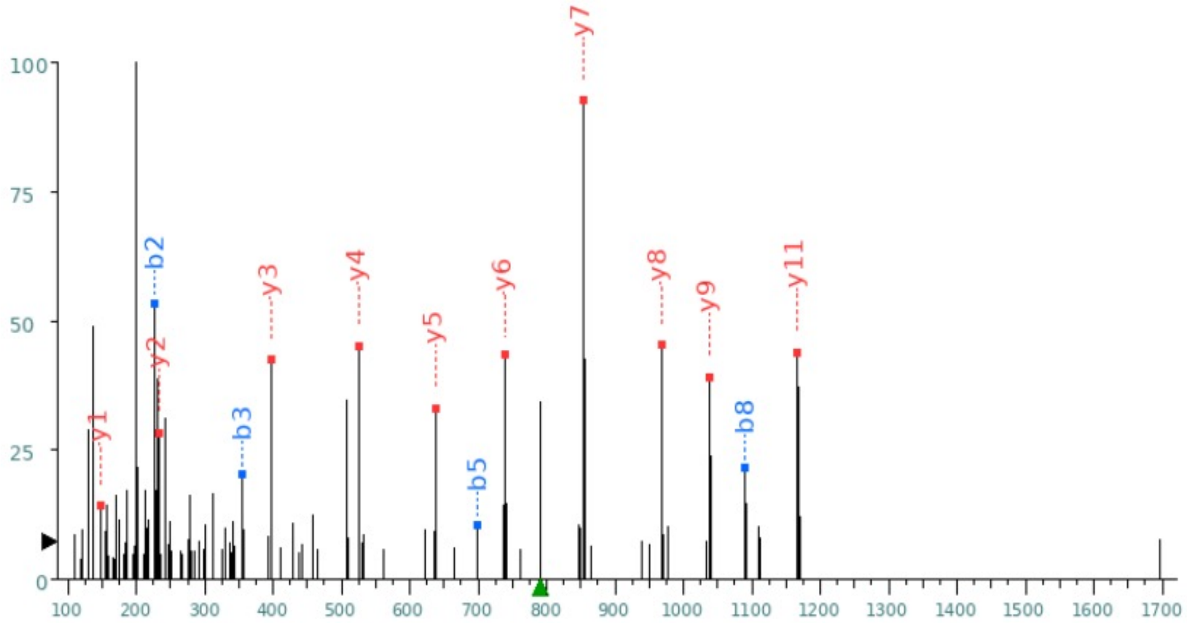

Sequence

LLQTK#NYDIGAALDTIQYSK

Predicted Fragmentation Pattern

+1

Seq	#	b: Δ Error	b	y	y: Δ Error	+1
L	1	---	114.091	---	---	20
L	2	2.870	227.175	2256.151	---	19
Q	3	2.139	355.234	2143.067	---	18
T	4	---	456.282	2015.008	---	17
K#	5	6.737	698.420	1913.960	---	16
N	6	---	812.462	1671.822	---	15
Y	7	---	975.526	1557.780	---	14
D	8	6.366	1090.553	1394.716	---	13
I	9	---	1203.637	1279.689	---	12
G	10	---	1260.658	1166.605	7.782	11
A	11	---	1331.695	1109.584	---	10
A	12	---	1402.733	1038.547	1.791	9
L	13	---	1515.817	967.509	3.819	8
D	14	---	1630.844	854.425	6.132	7
T	15	---	1731.891	739.398	0.353	6
I	16	---	1844.975	638.351	-1.105	5
Q	17	---	1973.034	525.267	2.527	4
Y	18	---	2136.097	397.208	1.534	3
S	19	---	2223.129	234.145	5.294	2
K	20	---	---	147.113	1.892	1

+2

Seq	#	b: Δ Error	b	y	y: Δ Error	+1
L	1	---	57.549	---	---	20
L	2	---	114.091	1128.579	---	19
Q	3	---	178.121	1072.037	---	18
T	4	---	228.644	1008.008	---	17
K#	5	---	349.713	957.484	---	16
N	6	---	406.735	836.415	---	15
Y	7	---	488.267	779.393	---	14
D	8	---	545.780	697.862	---	13
I	9	---	602.322	640.348	---	12
G	10	---	630.833	583.806	---	11
A	11	---	666.351	555.295	---	10
A	12	---	701.870	519.777	---	9
L	13	---	758.412	484.258	---	8
D	14	---	815.925	427.716	---	7
T	15	---	866.449	370.203	---	6
I	16	---	922.991	319.679	---	5
Q	17	---	987.021	263.137	---	4
Y	18	---	1068.552	199.108	---	3
S	19	---	1112.068	117.576	---	2
K	20	---	---	74.060	---	1

SQSTM1 K435

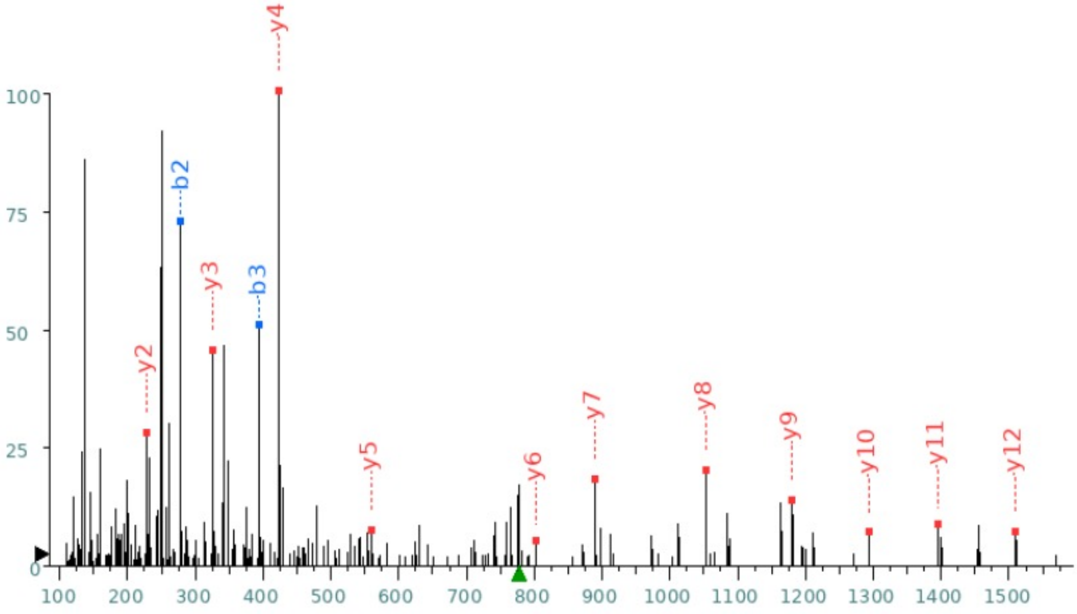

Sequence

NYDIGAALDTIQYSK#HPPPL

Predicted Fragmentation Pattern

+1

Seq	#	b: Δ Error	b	y	y: Δ Error	+1
N	1	---	115.050	---	---	20
Y	2	0.964	**278.114**	2213.124	---	19
D	3	-0.395	**393.140**	2050.060	---	18
I	4	---	506.225	1935.033	---	17
G	5	---	563.246	1821.949	---	16
A	6	---	634.283	1764.928	---	15
A	7	---	705.320	1693.891	---	14
L	8	---	818.404	1622.854	---	13
D	9	---	933.431	**1509.770**	8.108	12
T	10	---	1034.479	**1394.743**	9.189	11
I	11	---	1147.563	**1293.695**	3.357	10
Q	12	---	1275.622	**1180.611**	1.471	9
Y	13	---	1438.685	**1052.552**	2.331	8
S	14	---	1525.717	**889.489**	1.905	7
K#	15	---	1767.855	**802.457**	-1.481	6
H	16	---	1904.914	**560.319**	5.198	5
P	17	---	2001.966	**423.260**	0.927	4
P	18	---	2099.019	**326.207**	0.826	3
P	19	---	2196.072	**229.155**	1.238	2
L	20	---	---	132.102	---	1

+2

Seq	#	b: Δ Error	b	y	y: Δ Error	+1
N	1	---	58.029	---	---	20
Y	2	---	139.560	1107.065	---	19
D	3	---	197.074	1025.534	---	18
I	4	---	253.616	968.020	---	17
G	5	---	282.127	911.478	---	16
A	6	---	317.645	882.968	---	15
A	7	---	353.164	847.449	---	14
L	8	---	409.706	811.930	---	13
D	9	---	467.219	755.388	---	12
T	10	---	517.743	697.875	---	11
I	11	---	574.285	647.351	---	10
Q	12	---	638.314	590.809	---	9
Y	13	---	719.846	526.780	---	8
S	14	---	763.362	445.248	---	7
K#	15	---	884.431	401.732	---	6
H	16	---	952.960	280.663	---	5
P	17	---	1001.487	212.134	---	4
P	18	---	1050.013	163.607	---	3
P	19	---	1098.540	115.081	---	2
L	20	---	---	66.555	---	1

SQSTM1-Ub K63

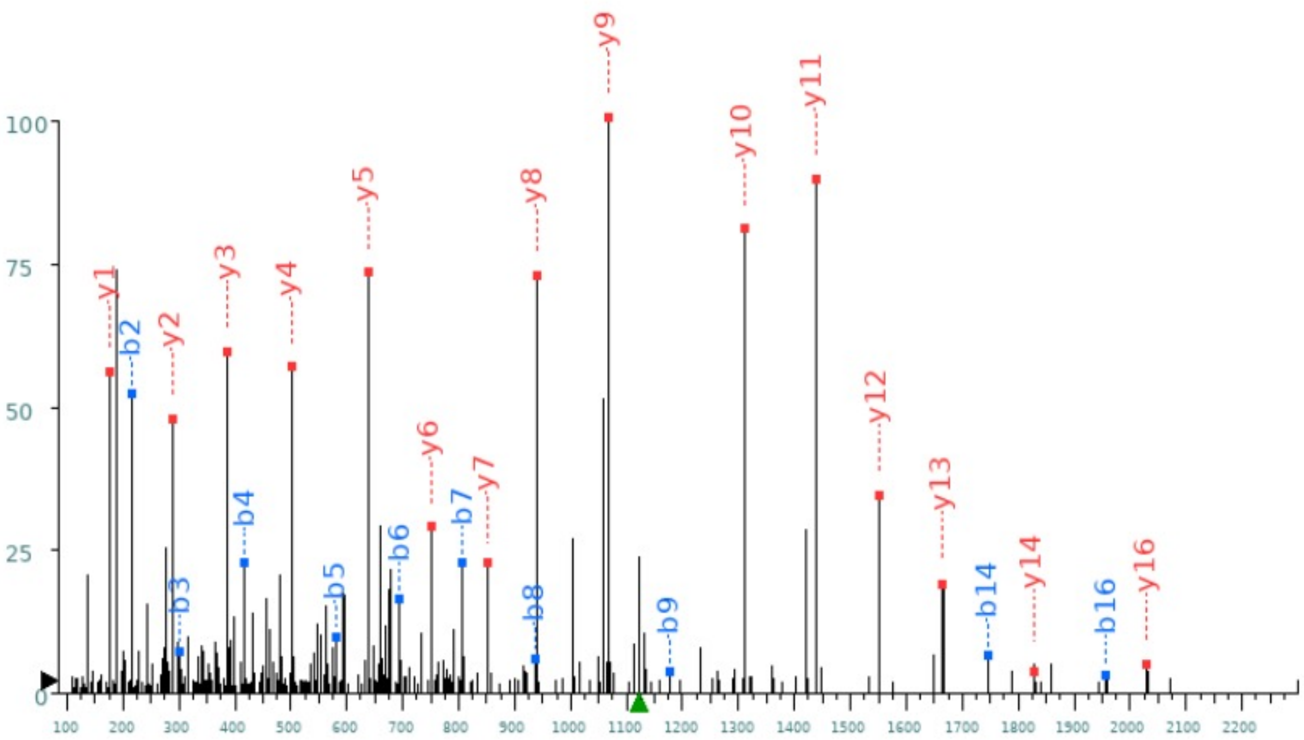

Sequence

TLSDYNIQK#ESTLHLVLR

Predicted Fragmentation Pattern

Seq	#	b: Δ Error	b	y	y: Δ Error	+1
T	1	---	102.055	---	---	18
L	2	2.077	215.139	2143.151	---	17
S	3	-0.996	302.171	2030.067	-0.662	16
D	4	-0.566	417.198	1943.035	---	15
Y	5	-2.045	580.261	1828.008	-1.130	14
N	6	3.801	694.304	1664.944	2.190	13
I	7	0.675	807.388	1550.901	4.607	12
Q	8	11.300	935.447	1437.817	3.327	11
K#	9	-1.607	1177.585	1309.759	2.055	10
E	10	---	1306.627	1067.621	1.904	9
S	11	---	1393.659	938.578	2.611	8
T	12	---	1494.707	851.546	-0.294	7
L	13	---	1607.791	750.498	1.713	6
H	14	1.005	1744.850	637.414	1.376	5
L	15	---	1857.934	500.355	1.596	4
V	16	5.505	1957.003	387.271	1.243	3
L	17	---	2070.087	288.203	1.329	2
R	18	---	---	175.119	1.858	1

SQSTM1-Ub K11

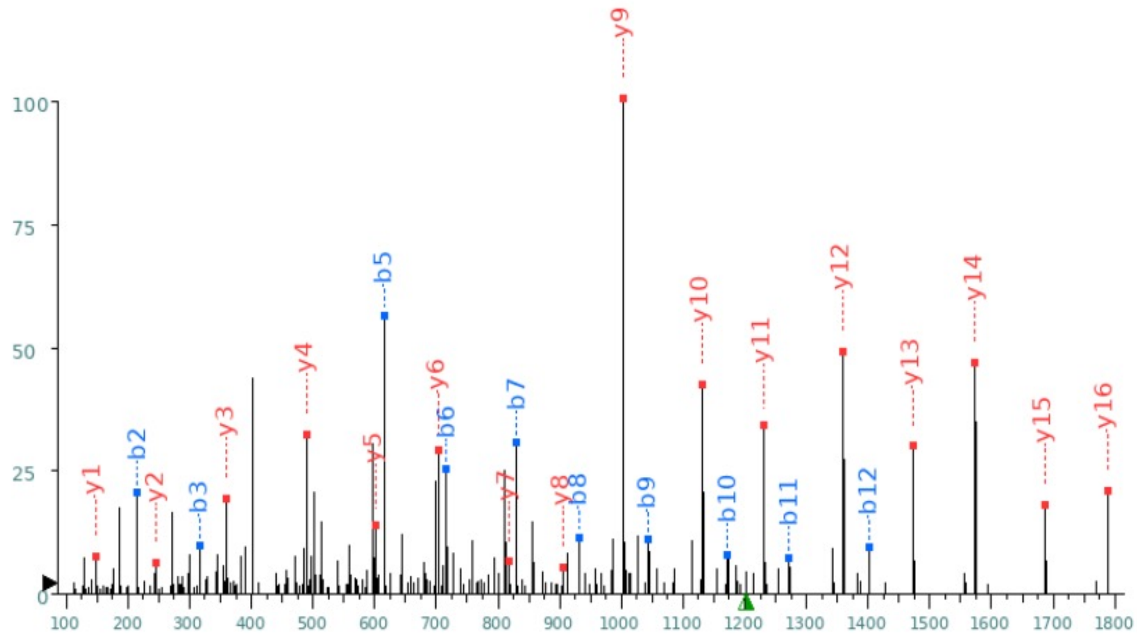

Sequence

TLTGK#TITLEVEPSDTIENVK

Predicted Fragmentation Pattern

Seq	#	b: Δ Error	b	y	y: Δ Error	+1
T	1	---	102.055	---	---	21
L	2	2.219	215.139	2301.218	---	20
T	3	0.416	316.187	2188.134	---	19
G	4	---	373.208	2087.087	---	18
K#	5	1.021	615.346	2030.065	---	17
T	6	1.715	716.394	1787.927	-3.718	16
I	7	4.255	829.478	1686.880	-0.136	15
T	8	7.849	930.525	1573.796	1.999	14
L	9	4.174	1043.610	1472.748	1.439	13
E	10	7.053	1172.652	1359.664	2.874	12
V	11	2.718	1271.721	1230.621	2.077	11
E	12	-0.140	1400.763	1131.553	3.493	10
P	13	---	1497.816	1002.510	2.472	9
S	14	---	1584.848	905.457	10.320	8
D	15	---	1699.875	818.425	4.612	7
T	16	---	1800.923	703.398	1.326	6
I	17	---	1914.007	602.351	0.856	5
E	18	---	2043.049	489.267	0.343	4
N	19	---	2157.092	360.224	1.541	3
V	20	---	2256.161	246.181	3.760	2
K	21	---	---	147.113	0.751	1
