# Supplementary material for: Enzymatic analysis of WWP2 E3 ubiquitin ligase using protein microarrays identifies autophagy-related substrates
Source: J Biol Chem. 2022 Mar 21;298(5):101854. doi: 10.1016/j.jbc.2022.101854 (PMC9034101; doi:10.1016/j.jbc.2022.101854)
Supplement: Supplementary Tables S1–S3 [file mmc3.pdf]

Table S1. Microarray negative control hits (ubiqutination signal from auto or pre-ubiquitiantion)	
**Protein Name**	**Protein Name**
SMAD3	GGA3
SMAD1	WBP1L
PRRG1	LAPTM4A
PRRG2	LDLRAD3
ARRDC4	HACE1
GRINA	RNF11
UBE2O	RNF111_frag
BIRC3	SYVN1
WDSUB1	GOSR2
RNF220	TMEM207
RNF181	DZIP3
RNF8	TMEM92
BIRC7	ARRDC1
NEDD4L	NDFIP1
RNF126	TRIM21
RNF115	PMEPA1
C9orf61	DGCR2
SPSB2	LAPTM5
FAM175B	RECQL
RFWD3	SPG20
CDH3	ZCCHC7
TMEM252	FAM189A2
RNF38	C11orf49
ANKRD13A	LDLRAD4
ANKRD13D	CBL
STUB1	RHBDD1
DNAJB2	MUC21
SMAD2	EPN1
ZFYVE28	BEAN1

Table S1. List of proteins that showed ubiquitination signals in negative control microarrays. These signals might be due to auto-ubiquitination or these proteins were ubiquitinated during yeast protein preparation in yeast. These hits have been removed from either WWP2 Y369E or WT microarrays.

Table S2. WT WWP2 Protein microarray hits		
**Protein Name**	**Ub Signal (Z value)**	**Previous reports**
WASF2	4.9	No
KHDRBS3	4.3	No
IST1	3.5	No

Table S2. List of identified WWP2 E3 ligase hits using HuProt protein microarrays using wild-type ligase. The hits are ranked by ubiquitination signal intensity/Z value.

Table S3. WWP2 substrate hits identified by protein microarray		
**Protein Name**	**Previous reports**	**Reference**
USP5	High-throughput mass spect hit	(1)
OPTN	No	
CALCOCO2	No	
PSMD4	No; Reported NEDD4 substrate	(2,3)
STAM	No; Reported ITCH substrate	(4)
SQSTM1	No; Reported NEDD4 substrate	(5,6)
UBAP1	No	
RAD23A	No	
FUT3	No	
UBQLN4	No	
FAF1	No	
HGS	No; Reported NEDD4 substrate	(7)
ATXN3	No	
TOM1	No; Reported NEDD4 substrate by HT	(8)
TOM1L2	No; Reported NEDD4 substrate by HT	(8)
UBE2D4	No	
CDC34	Identified binding partner	(9)
RAD23B	No	
SFMBT1	No	
Ubl7	No	
TRIP10	No	
UBQLN1	No	
FAM131C	No	
MED4	No	
CFAP36	No	
RABEP2	No	
PTEN	Well identified Substrate	(10-14)
UBE2D3	Identified binding partner	(15,16)
UBAC1	No	
C3orf70	No	
FAM131B	No	
THBS1	No	

Table S3. List of identified WWP2 E3 ligase hits using HuProt protein microarrays using linker phospho-mimetic form of WWP2. The hits are annotated with their previous reported correlation with WWP2 and the corresponding literature.

1 Caron, P., Pankotai, T., Wiegant, W. W., Tollenaere, M. A., Furst, A., Bonhomme, C., Helfricht, A., de Groot, A., Pastink, A., and Vertegaal, A. C. (2019) WWP2 ubiquitylates RNA polymerase II for DNA-PK-dependent transcription arrest and repair at DNA breaks. *Genes & development* **33**, 684-704

2 Puig-Sàrries, P., Bijlmakers, M.-J., Zuin, A., Bichmann, A., Pons, M., and Crosas, B. (2015) An intrinsically disordered region of RPN10 plays a key role in restricting ubiquitin chain elongation in RPN10 monoubiquitination. *Biochemical Journal* **469**, 455-467

3 Todaro, D. R., Augustus-Wallace, A. C., Klein, J. M., and Haas, A. L. (2018) Oligomerization of the HECT ubiquitin ligase NEDD4-2/NEDD4L is essential for polyubiquitin chain assembly. *Journal of Biological Chemistry* **293**, 18192-18206

4 Malik, R., Soh, U. J., Trejo, J., and Marchese, A. (2012) Novel roles for the E3 ubiquitin ligase atrophin-interacting protein 4 and signal transduction adaptor molecule 1 in G protein-coupled receptor signaling. *Journal of Biological Chemistry* **287**, 9013-9027

5 Lin, Q., Dai, Q., Meng, H., Sun, A., Wei, J., Peng, K., Childress, C., Chen, M., Shao, G., and Yang, W. (2017) The HECT E3 ubiquitin ligase NEDD4 interacts with and ubiquitylates SQSTM1 for inclusion body autophagy. *Journal of cell science* **130**, 3839-3850

6 Sun, A., Wei, J., Childress, C., Shaw IV, J. H., Peng, K., Shao, G., Yang, W., and Lin, Q. (2017) The E3 ubiquitin ligase NEDD4 is an LC3-interactive protein and regulates autophagy. *Autophagy* **13**, 522-537

7 Katz, M., Shtiegman, K., Tal-Or, P., Yakir, L., Mosesson, Y., Harari, D., Machluf, Y., Asao, H., Jovin, T., and Sugamura, K. (2002) Ligand-independent degradation of epidermal growth factor receptor involves receptor ubiquitylation and Hgs, an adaptor whose ubiquitin-interacting motif targets ubiquitylation by Nedd4. *Traffic* **3**, 740-751

8 Persaud, A., Alberts, P., Amsen, E. M., Xiong, X., Wasmuth, J., Saadon, Z., Fladd, C., Parkinson, J., and Rotin, D. (2009) Comparison of substrate specificity of the ubiquitin ligases Nedd4 and Nedd4-2 using proteome arrays. *Molecular systems biology* **5**, 333

9 Sheng, Y., Hong, J. H., Doherty, R., Srikumar, T., Shloush, J., Avvakumov, G. V., Walker, J. R., Xue, S., Neculai, D., and Wan, J. W. (2012) A human ubiquitin conjugating enzyme (E2)-HECT E3 ligase structure-function screen. *Molecular & Cellular Proteomics* **11**, 329-341

10 Maddika, S., Kavela, S., Rani, N., Palicharla, V. R., Pokorny, J. L., Sarkaria, J. N., and Chen, J. (2011) WWP2 is an E3 ubiquitin ligase for PTEN. *Nature cell biology* **13**, 728-733

11 Chen, Z., Thomas, S. N., Bolduc, D. M., Jiang, X., Zhang, X., Wolberger, C., and Cole, P. A. (2016) Enzymatic analysis of PTEN ubiquitylation by WWP2 and NEDD4-1 E3 ligases. *Biochemistry* **55**, 3658-3666

12 Chen, Z., Jiang, H., Xu, W., Li, X., Dempsey, D. R., Zhang, X., Devreotes, P., Wolberger, C., Amzel, L. M., and Gabelli, S. B. (2017) A tunable brake for HECT ubiquitin ligases. *Molecular cell* **66**, 345-357. e346

13 Bawa-Khalfe, T., Yang, F.-M., Ritho, J., Lin, H.-K., Cheng, J., and Yeh, E. T. (2017) SENP1 regulates PTEN stability to dictate prostate cancer development. *Oncotarget* **8**, 17651

14 Zhang, H., Wei, P., Lv, W., Han, X., Yang, J., and Qin, S. (2019) Long noncoding RNA lnc-DILC stabilizes PTEN and suppresses clear cell renal cell carcinoma progression. *Cell & bioscience* **9**, 1-13

15 Maspero, E., Valentini, E., Mari, S., Cecatiello, V., Soffientini, P., Pasqualato, S., and Polo, S. (2013) Structure of a ubiquitin-loaded HECT ligase reveals the molecular basis for catalytic priming. *Nature structural & molecular biology* **20**, 696-701

16 Kristariyanto, Y. A., Choi, S.-Y., Rehman, S. A. A., Ritorto, M. S., Campbell, D. G., Morrice, N. A., Toth, R., and Kulathu, Y. (2015) Assembly and structure of Lys33-linked polyubiquitin reveals distinct conformations. *Biochemical Journal* **467**, 345
